# Epilepsy, Antiepileptic Drugs, and Aggression: An Evidence-Based Review[Fn FN2]

**DOI:** 10.1124/pr.115.012021

**Published:** 2016-07

**Authors:** Martin J. Brodie, Frank Besag, Alan B. Ettinger, Marco Mula, Gabriella Gobbi, Stefano Comai, Albert P. Aldenkamp, Bernhard J. Steinhoff

**Affiliations:** Epilepsy Unit, West Glasgow Ambulatory Care Hospital–Yorkhill, Glasgow, Scotland (M.J.B.); East London National Health Service Foundation Trust, Bedford, United Kingdom (F.B.); University College London School of Pharmacy, London, United Kingdom (F.B.); Winthrop University Hospital, Mineola, New York (A.B.E.); Epilepsy Group, Atkinson Morley Regional Neuroscience Centre, St. George’s University Hospitals National Health Service Foundation Trust, London, United Kingdom (M.M.); Institute of Medical and Biomedical Sciences, St. George’s, University of London, London, United Kingdom (M.M.); Neurobiological Psychiatry Unit, Department of Psychiatry, McGill University, Montreal, Quebec, Canada (G.G., S.C.); McGill University Health Center, McGill University, Montreal, Quebec, Canada (G.G., S.C.); Division of Neuroscience, San Raffaele Scientific Institute and Vita-Salute University, Milan, Italy (S.C.); Epilepsy Centre Kempenhaeghe, Heeze, The Netherlands (A.P.A.); Maastricht University Medical Centre, Maastricht, The Netherlands (A.P.A.); and Kork Epilepsy Centre, Kehl-Kork, Germany (B.J.S.)

## Abstract

Antiepileptic drugs (AEDs) have many benefits but also many side effects, including aggression, agitation, and irritability, in some patients with epilepsy. This article offers a comprehensive summary of current understanding of aggressive behaviors in patients with epilepsy, including an evidence-based review of aggression during AED treatment. Aggression is seen in a minority of people with epilepsy. It is rarely seizure related but is interictal, sometimes occurring as part of complex psychiatric and behavioral comorbidities, and it is sometimes associated with AED treatment. We review the common neurotransmitter systems and brain regions implicated in both epilepsy and aggression, including the GABA, glutamate, serotonin, dopamine, and noradrenaline systems and the hippocampus, amygdala, prefrontal cortex, anterior cingulate cortex, and temporal lobes. Few controlled clinical studies have used behavioral measures to specifically examine aggression with AEDs, and most evidence comes from adverse event reporting from clinical and observational studies. A systematic approach was used to identify relevant publications, and we present a comprehensive, evidence-based summary of available data surrounding aggression-related behaviors with each of the currently available AEDs in both adults and in children/adolescents with epilepsy. A psychiatric history and history of a propensity toward aggression/anger should routinely be sought from patients, family members, and carers; its presence does not preclude the use of any specific AEDs, but those most likely to be implicated in these behaviors should be used with caution in such cases.

## I. Introduction

The past 20 years have seen the introduction of >15 antiepileptic drugs (AEDs), many with unique mechanisms of action (Löscher et al., 2013). Nevertheless, >30% of adolescent and adult patients with the common epilepsies continue to have seizures, despite receiving treatment with many of these drugs used either singly or in combination (Brodie et al., 2012). Outcomes in childhood epilepsies, excluding the genetic encephalopathies of infancy, are equally disappointing (Geerts et al., 2010). In parallel with these pharmacological developments has come an increasing awareness that people with epilepsy, possibly as many as 30% of the newly diagnosed population and up to 50% of patients with pharmacoresistant epilepsy, have complex psychiatric, behavioral, cognitive, and social problems (Lin et al., 2012). Indeed, these problems often precede the onset of epilepsy (Hesdorffer et al., 2012). The presence of psychiatric comorbidities contributes to the likelihood that seizures will prove resistant to both AEDs and epilepsy surgery (Hitiris et al., 2007; Kanner et al., 2009; Petrovski et al., 2010). The situation is further complicated by the beneficial psychotropic effects of some AEDs and the adverse properties of others (Piedad et al., 2012). Behavioral side effects that have been associated with AEDs include depression, aberrant behaviors, and the development or worsening of irritability, impulsivity, anger, hostility, and aggression. Although prior reviews have focused on the associations between AEDs and depression or aberrant behaviors, the specific topic of aggression in response to AEDs has been largely neglected. We have endeavored in this evidence-based review to explore the neurobiology, epidemiology, presentation, clinical relevance, and management of issues relating to aggression in children, adolescents, and adults with newly diagnosed and chronic epilepsy exposed to a range of established and modern AEDs.

## II. Aggressive Behavior in Epilepsy: Definitions

Aggressive behavior in epilepsy has been the subject of many misconceptions and controversies (Schachter, 2007). In the context of seizures, aggressive behaviors have been observed in the preictal, ictal, and postictal states (before, during, and after the seizure, respectively), although directed and purposeful ictal aggression has only rarely been observed (Delgado-Escueta et al., 1981). Interictal aggressive behaviors (during periods between seizures) have sometimes been attributed to the irritability described in what some have termed an “interictal dysphoric disorder” of epilepsy (Blumer, 1997).

Medications, including some AEDs, have been associated with the induction or exacerbation of adverse psychotropic effects, including aggression (Ettinger, 2006). Our knowledge of aggression and related effects such as irritability is based on reviews of predominantly spontaneous reporting of psychiatric symptoms in clinical case experience or in premarketing drug trials. One challenge in determining the rate and nature of AED-induced aggression is the fact that most studies are focused on the antiseizure efficacy of AEDs, as well as on the capture of more traditional potential adverse events (AEs) such as fatigue or rash, and do not rigorously assess psychiatric symptoms. Furthermore, the terminology for aggression and related terms is not well defined and is not universally accepted even among experts in the field of psychiatry.

Some measures have been developed to detect or rate aggression-related behaviors, but these are rarely used in the context of premarketing trials of AEDs. Instead, psychiatric symptoms are typically reported by patients using informal terms that do not adhere to strict diagnostic criteria. These informal terms are usually standardized and categorized using the *Medical Dictionary for Regulatory Activities* (MedDRA), an internationally endorsed dictionary and thesaurus for medical terminology. For example, when a patient reports “feeling queasy” and this is entered into the study database, this is categorized into a more specific “preferred term” (nausea) to which other related symptom descriptors are also linked. Related preferred terms are grouped into high-level terms (nausea and vomiting), which are in turn grouped into high-level group terms and ultimately system organ classes (gastrointestinal disorders). Standardized MedDRA queries (SMQs) have been developed (and extensively reviewed and tested) that can look across these groupings for terms that are related to the condition of interest. SMQs can be narrow (terms that are highly likely to represent the condition of interest) and thus have high specificity but low sensitivity; or they can be broad, with higher sensitivity at the expense of more false positives (i.e., will select some AEs that will, on inspection, not be related to the event in question). Thus, the narrow SMQ for hostility/aggression identifies AE terms that are very likely to be related (e.g., “anger” and “physical assault”). The broad SMQ also includes broadly related terms, such as “skin laceration,” that will often not be related to hostility or aggression but will be a direct result in some cases.

We considered agitation, anger, hostility, impulsivity, and irritability as the most important terms that are related to and encompass aggression and related behaviors. We therefore used these terms to direct our searches for evidence of aggression associated with AEDs, and we review the terms here in the way in which they are traditionally used in the psychiatric literature. None of these terms themselves are individual diagnoses in the fifth edition of the *Diagnostic and Statistical Manual of Mental Disorders* (DSM), but some (particularly irritability, impulsivity, and aggression) are used to describe important components and symptoms of psychiatric diagnoses (American Psychiatric Association, 2013).

### A. Aggression

Even in the fields of psychology or psychiatry, terms such as aggression, anger, hostility, or irritability have been used loosely and interchangeably. Proposed definitions have gone through numerous iterations. Dollard et al. (1939) defined aggression as “the affectively driven attack on another with the intent to do harm.” Feshbach (1964) proposed a distinction between anger-motivated hostile aggression and instrumental aggression (in which anger and intent to harm could be absent but harm does result). DiGiuseppe and Tafrate (2007) returned to the concept of intent to harm, defining aggression as “overt motor behaviour enacted with the intent to do harm or injury to a person or object, with the expectation that harm will occur.” Associated affective states may include irritation, frustration, fear, and even pleasure (Campbell, 2009).

Aggression may appear as “hostile, threatening, and violent behaviors” (Onyike and Lyketsos, 2011). It has also been noted that “behaviors range from assertiveness to coercion (including the use of force) and from hostile attitudes and verbal abuse to threats, belligerence, and violence” (Campbell, 2009). There may be no, trivial, or minimal provocation (Onyike and Lyketsos, 2011).

Aggression may be a symptom of diverse psychiatric conditions, including “delusional psychoses, dementia, agitated delirium, intoxication, conduct disorder in children or personality disorders (particularly antisocial, borderline, paranoid, and narcissistic types) in adults, and even adjustment disorder” (Onyike and Lyketsos, 2011). Aggression may also complicate nonpsychiatric illnesses because it can develop when patients feel disregarded or “angered by perceived unfairness or mistreatment, or as a ‘primary’ symptom of the illness” (Onyike and Lyketsos, 2011). Aggressive behaviors have also been described as an adverse psychotropic effect of medications such as AEDs (Dinkelacker et al., 2003). Indeed, Gollan et al. (2005) distinguished medically related aggression from premeditated or impulsive types.

From an evolutionary perspective, aggression may have developed in humans for self-preservation (e.g., protecting offspring) but also for “retaliation, material advantage, and power” (Onyike and Lyketsos, 2011). It can serve as a behavior designed to attain a goal or defend against threats (Miczek et al., 2002; Takahashi et al., 2011). Aggression is a manifestation of “appetitive drives” in some situations, whereas it is a defensive behavior in others (Onyike and Lyketsos, 2011).

### B. Agitation

Agitation is “a state of pathologically intense emotional arousal and motor restlessness” (Onyike and Lyketsos, 2011), typically associated with hyperactive behaviors such as handwringing or aimlessly pacing (Campbell, 2009) or “cursing, screaming, biting, and fighting, and it may evolve through a verbally or physically aggressive behavior” (Comai et al., 2012a,b). It is characterized by “inappropriate verbal, vocal, or motor activity that is not explained by apparent needs or confusion per se” (Comai et al., 2012a).

### C. Anger

Anger was defined by Spielberger (1996) as “an emotional state, varying in intensity from mild annoyance to rage, that is accompanied by arousal of the autonomic nervous system.” Spielberger distinguished the anger state (the experience of these emotions) from trait (a tendency toward recurrently experiencing these emotions). DiGiuseppe and Tafrate (2007) proposed a more multidimensional construct defining anger as “a constellation of specific uncomfortable subjective experiences and associated cognitions (e.g., thoughts, beliefs, images) that have variously associated verbal, facial, bodily, and automatic reactions.” They further emphasize that anger is experienced in “people’s conscious awareness and is communicated through verbalizations and bodily reactions” (DiGiuseppe and Tafrate, 2007).

### D. Hostility

An older literature describes hostility in the context of personality traits: “an attitude of resentment, suspiciousness, and bitterness coupled with the desire to get revenge or to have destructive goals for one’s anger” (Endler and Hunt, 1968). Spielberger (1988) defined it as “the disposition to perceive a wide range of situations as annoying or frustrating, and the tendency to respond to such situations with more frequent elevations in state anger.” Both Spielberger (1988) and Barefoot (1992) divide hostility into cognitive components (e.g., negative beliefs, cynicism, mistrust), an affective or emotional component (i.e., varying degrees of anger), and the behavioral component (verbal or physical assault with intent to cause harm), whereas others contend that the term “hostility” should be reserved for the cognitive component (Vollrath, 2006).

### E. Impulsivity

Impulsivity may be described as “acting without control or premeditation” (Comai et al., 2012a) or “behaving recklessly without regard to consequences” (Hollander et al., 2000). In less formal terms, it may be conceptualized as “acting without thinking” (Barratt, 2000) or without self-restraint, with a tendency toward “hair-trigger” actions (Onyike and Lyketsos, 2011).

*Campbell’s Psychiatric Dictionary* defines impulsivity as “a predisposition toward rapid, unplanned reactions to internal or external stimuli with diminished regard to the negative consequences of these reactions to the impulsive individual or others.” Campbell (2009) further notes it to be “a pattern of behavior consisting of rapid, unplanned actions which occur unexpectedly, without reflection or conscious judgment, and without regard for possible consequences.”

### F. Irritability

Irritability is a term that is commonly used in DSM-5 (American Psychiatric Association, 2013) as a component of psychiatric diagnoses such as major depressive disorder or generalized anxiety disorder (DiGiuseppe and Tafrate, 2007), but the term itself is not defined there. Irritability also accompanies many neurologic disorders such as dementia and is a component of the controversial interictal dysphoric disorder of epilepsy (Blumer et al., 2004; Amiri and Hansen, 2015).

Work by Born and Steiner (1999), as summarized by DiGiuseppe and Tafrate (2007), described five components of irritability: “1) a heightened or excessive sensitivity to external stimuli, 2) a negative affective state, 3) a state of physical and psychological tension that may suddenly and rapidly escalate, 4) reduced control over temper, proneness to anger, annoyance, or impatience, and 5) irascible verbal behavior outbursts, or even explosive behavior.”

Piazzini emphasized the extreme sensitivity to stimulation of any kind and excessive response to “environmental, situational and emotional stimuli” (Caprara et al., 1992; Piazzini et al., 2011). Coccaro et al. (1991, 2000) define irritability succinctly as “a tendency to respond with negative affect in reaction to aversive stimuli or with hypersensitivity to aversive stimuli.”

To summarize, although there are diverse terms that relate to the concept of aggressive behaviors, this review uses the phrases “aggression” and “aggressive behaviors” to express the broad range of associated concepts including agitation, anger, hostility, impulsivity, and irritability.

## III. Aggressive Behavior in Epilepsy: Clinical Aspects

There is clear evidence that psychiatric disorders are more frequently encountered in patients with epilepsy than in the general population, with prevalence rates in the range of 20%–30% for mood and anxiety disorders and 2%–7% for psychoses (Lin et al., 2012).

In general terms, the wide range of manifestations of aggression described earlier have been reported by many different authors in people with epilepsy (Bach-y-Rita et al., 1971; Maletzky, 1973; Maletzky and Klotter, 1974; Ratner and Shapiro, 1979; Leicester, 1982; Elliott, 1984, 1990; Stone et al., 1986; van Elst et al., 2000). However, in several cases, it was a general impression of aggression, not supported by scientific evidence, and was possibly influenced by old-fashioned prejudices about epilepsy (de Boer et al., 2008; Monaco and Mula, 2011). In fact, studies on the true prevalence of aggressive behaviors in people with epilepsy are scarce.

As stated earlier, psychiatric symptoms in epilepsy have been historically classified according to their temporal relation with seizures as peri-ictal, ictal, and interictal. Ictal symptoms are the clinical expression of an epileptic seizure. Peri-ictal refers to symptoms before (preictal) or after (postictal) the seizure, whereas interictal symptoms are those that occur in no clear time relationship to the seizures (Table 1).

**TABLE 1:** Classification of aggressive symptoms according to their temporal relationship with seizures

Timing relative to seizure	Aggressive symptoms reported
Peri-ictal	
Preictal	Not reported
Ictal	Aggressive conduct in 1 of 1000 seizures recorded in monitoring units (Delgado-Escueta et al., 1981)
Postictal	Postictal delirium/confusion
	Postictal psychoses (22.8% present with aggressive behaviors)
Paraictal	
Forced normalization	Rarely reported
Interictal	Up to 7% in unselected groups but due to the underlying psychiatric comorbidity

Peri-ictal aggression is often associated with confusion or psychosis. Although preictal (prodromal) aggressive behavior has been described (Hughes et al., 1993; Devinsky, 2003), very little detail has been published. Aggressive behavior has been reported in 22.8% of cases of postictal psychoses (Kanemoto et al., 2010). Aggressive behavior as an ictal phenomenon is extremely rare. A large survey of several thousand seizures documented on video electroencephalograms (EEGs) reported an incidence rate of 1 in 1000 for aggressive conduct during seizure (Delgado-Escueta et al., 1981). However, in all of these cases, violent motor automatisms during seizures were misinterpreted as threatening or assaultive. In fact, although the aggressive act may appear orchestrated, it is poorly directed and does not involve intricate skills or purposeful and detailed behaviors (Marsh and Krauss, 2000). The aggressive conduct is directed toward nearby objects or persons, involving mainly pushing and shoving. Typical epileptic phenomena, such as staring or oral and motor automatisms, may be present. The patient is usually amnestic for these episodes, expressing subsequent profound remorse (Devinsky and Bear, 1984; Herzberg and Fenwick, 1988; Fenwick, 1989). In the few cases reported from a series in monitoring units, aggressive automatisms were shown to be related to epileptic activity rising from the amygdala and spreading through the diencephalic regions (Lee et al., 1998). No clear lateralizing features were described, although associated symptoms point to the nondominant hemisphere (Marsh and Krauss, 2000). The attribution of violent behaviors to an ictal event is not always simple, and video-EEG monitoring always elucidates whether or not behaviors are associated with a seizure. Treiman (1986) recommended five criteria to determine whether a specific violent act was the result of an epileptic seizure: 1) the patient should have an established diagnosis of epilepsy, 2) there should be video-EEG documentation of epileptic automatisms, 3) there should be video-EEG documentation of the aggressive behavior, 4) the aggressive act should be characteristic of the patient’s habitual seizures, and 5) a clinical judgment should be made by the neurologist as to the possibility that the violent act was part of a seizure (Marsh and Krauss, 2000). These video-EEG studies also revealed that aggressive behavior tended to occur as “resistive” violence, typically when an attendant went to assist the patient during ictal or postictal confusional states; the implication is that the patient may have misinterpreted the attendant's actions in the patient’s confusional state and may consequently have resisted aggressively (Treiman, 1986).

Therefore, the available data clearly indicate that aggressive behavior in epilepsy is most commonly unrelated to seizures themselves. A recent article focusing on homicide identified 30 articles and 176 cases (published up to 2013) involving alleged perpetrators with epilepsy (Pandya et al., 2013). In 78% of cases, there was no temporal relationship between the homicide and the seizures. In the remaining 22%, the violent episode occurred as a postictal event in the majority of cases (82%). Patients were usually young male individuals, with low average intelligence and a history of behavioral problems starting during childhood. Alcohol abuse and stressful situations were precipitating factors. This is of interest when we consider psychiatric disorders associated with aggressive behavior. DSM-5 states that aggressive behavior can occur in association with disruptive, impulse control, or conduct disorders or antisocial personality disorder (American Psychiatric Association, 2013). These disorders are all characterized by problems in emotional and behavioral self-control and often start during childhood. Data from the Epidemiology Catchment Area survey reported a 1-year prevalence of violent behaviors of 2.05% (2.7% and 1.1% for male and female individuals, respectively) among respondents without any psychiatric disorder (Swanson et al., 1990). In the large DSM-5 chapter on impulse control disorders, intermittent explosive disorder is the most pertinent in this discussion. It is characterized by aggressive outbursts that should be impulse and/or anger based in nature and must cause marked distress, must cause impairment in occupational or interpersonal functioning, or must be associated with negative financial or legal consequences. According to the DSM criteria (American Psychiatric Association, 2013), antisocial personality disorder is defined by a pervasive pattern of disregard for the rights of other people that often manifests as hostility and/or aggression. It also starts during childhood. Conduct disorder is often considered the precursor of the antisocial personality disorder. As for impulse control disorders, patients with antisocial personality disorder frequently act on impulsive urges without considering the consequences. This difficulty with impulse control can result in loss of employment, accidents, legal difficulties, and incarceration. Remorse is quite common after behaviors that are the result of poor impulse control. In contrast, a typical and distinguishing feature for patients with antisocial personality disorder is the lack of genuine remorse for the harm they cause others, although these patients can become adept at feigning remorse when it is in their best interest to do so.

The lack of data about the prevalence of these disorders in patients with epilepsy is quite striking. Despite the huge volume of publications on the controversial issue of personality changes in epilepsy, we could find no studies that have investigated antisocial personality disorder or impulse control disorders in adults with epilepsy; however, it is of interest to note that in childhood epilepsy, the prevalence of attention deficit hyperactivity disorder (ADHD; in which impulsivity is a core feature) is common, at around 20%–30% (Hermann et al., 2007).

Studies investigating interictal aggressive symptoms are also limited. In the 1970s, Rodin (1973) reported the prevalence of aggressive behavior as 4.3% in unselected samples of patients with epilepsy, whereas Currie et al. (1971) reported prevalence of up to 7%. More recently, a multicenter study using a newly developed questionnaire suggested that people with epilepsy have slightly less aggressive behaviors than the general population and that cognitive impairment and polytherapy are the major associated variables (Piazzini et al., 2011). However, significantly more aggressive behavior was present among patients without comorbid psychiatric disorders than patients with psychiatric comorbidities. Prevalence rates for aggression were not reported, because aggressive symptoms were reported as a dimension. One research group has investigated the neuroanatomical correlates of aggressive behavior in temporal lobe epilepsy (TLE), showing a reduction in neocortical gray matter in the frontal areas and amygdala atrophy but no association with hippocampal pathology (van Elst et al., 2000; Woermann et al., 2000).

## IV. Neurobiology and Psychopharmacology of Epilepsy and Aggression

### A. Epilepsy and Aggression: Neurobiological and Neuropharmacological Correlates

Aggressive behavior is one of the psychiatric disorders that has long been described in people with epilepsy (Kligman and Goldberg, 1975; van Elst et al., 2000; Sumer et al., 2007). Both the epilepsy itself (Marsh and Krauss, 2000) and AED use have been suggested as factors that can increase the risk of aggressive behavior in patients with epilepsy. In this section, we examine this topic from a neurobiological and psychopharmacological perspective.

The underpinnings of aggression (Siever, 2008; Comai et al., 2012a) and epilepsy (Engel et al., 2007; Scharfman, 2007; Moshé et al., 2015) are clearly multifaceted, and some forms of pathologic aggression associated with epilepsy have an underlying neurobiology that we are only beginning to understand. Although well characterized and approved pharmacological strategies are currently available for the treatment of the different forms of epilepsy (Kwan et al., 2001; Moshé et al., 2015), national and international guidelines for the pharmacotherapy of aggression are still controversial and regulatory drug agencies do not presently consider aggressive behavior as a distinct disease (Comai et al., 2012b). Consequently, no medication is specifically approved for the treatment of aggression. One of the main reasons behind this lack of guidelines is that the complex neurobiological basis of aggressive behavior has not yet been elucidated by fundamental and clinical research.

### B. Neuropharmacology of Epilepsy

Seizures, at a basic level, originate from an imbalance between excitatory and inhibitory inputs to cells (i.e., increased excitation or decreased inhibition). The result is an abnormal synchronization of electrical activity in a group of active neurons and, depending on the site of origin and the subsequent brain structures and networks affected, seizures may produce a variety of clinical features and symptoms and may remain localized or generalize across the entire brain. Epilepsy is a network disorder in which the normal physiologic connections between cortical and subcortical pathways/regions are interrupted or disturbed.

The terminology and classification of seizures in epilepsy is currently under revision. Seizures are broadly defined as either focal, which originate within networks in one hemisphere, or generalized, which originate in and rapidly engage bilaterally distributed networks (Berg et al., 2010). The older term “partial onset” is no longer preferred, and the distinctions between primary and secondarily generalized (i.e., affecting both hemispheres from the outset, or progressing to both hemispheres after a focal onset) are not preserved in the new classification but are still in widespread use. Seizures can then be described in terms of their type (e.g., generalized tonic–clonic, absence, myoclonic, clonic, tonic, or atonic) and by their underlying cause (genetic versus structural/metabolic versus unknown). Many syndromes are also defined, such as juvenile myoclonic epilepsy and Lennox–Gastaut syndrome, which are characterized by complex and specific clinical features, signs, and symptoms that frequently cluster together (Berg et al., 2010).

At a cellular level, the electrical activity of neurons is under the control of ion transporters, pumps, and ion channels, which allow amounts of positively or negatively charged ions to flow in and out of cells. In turn, these pumps and ions channels are regulated by factors such as voltage or the binding of ligands either directly or via G protein–coupled receptors (Kandel et al., 2000; Dascal, 2001; Bean, 2007). The pivotal channels in these processes are Na^+^, K^+^, Ca^2+^, and Cl^−^ channels, which are also the target of many of the current available AEDs (Table 2).

**TABLE 2:** AED targets and effect on aggression in patients without epilepsy Missing data do not necessarily imply that there is no action on a specific target. In particular, research on the effects of novel and recent AEDs on epigenetic regulation and/or gene expression is still ongoing. Unless otherwise specified, mechanism-of-action details may be found in the text of this review, in review articles (Rogawski and Löscher, 2004; Brodie, 2010; Comai et al., 2012b), and/or in the summary of product characteristics for each individual AED.

AED	Main System Targeted	Other Possible Systems Targeted	Epigenetic Mechanisms/ Gene Expression	Antiaggressive in Psychiatric Patients*^f^*
Carbamazepine	Na^+^ channels		X	X
Eslicarbazepine acetate	Na^+^ channels		X*^b^*	
Lacosamide	Na^+^ channels			
Lamotrigine	Na^+^ channels	Glutamate (AMPA receptors)	X	X
Oxcarbazepine	Na^+^ channels			X
Phenytoin	Na^+^ channels		X*^c^*	X
Rufinamide	Na^+^ channels			
Zonisamide	Na^+^ and Ca^2+^ channels	GABA	X*^d^*	
Ethosuximide	T-type Ca^2+^ channels			
Gabapentin	Ca^2+^ channels	GABA, glutamate (NMDA receptors)		X
Pregabalin	Ca^2+^ channels	GABA		
Retigabine	K^+^ channels	GABA*^a^*		
Felbamate	Mixed	Ca^2+^ channels, NMDA receptors, GABA receptors		
Topiramate	Mixed	GABA_A_ receptors, AMPA/kainate receptors, Na^+^/Ca^2+^ channels	X	X
Phenobarbital	GABA			
Clobazam/clonazepam	GABA (GABA_A_ receptor)			X
Vigabatrin	GABA		X*^e^*	
Tiagabine	GABA			X
Stiripentol	GABA			
Levetiracetam	Neurotransmitter release (via SV2A)		X	X
Brivaracetam	Neurotransmitter release (via SV2A)			
Perampanel	Glutamate (AMPA receptors)			
Valproic acid	Mixed	GABA, glutamate (NMDA, AMPA, and kainate receptors), Na^+^ channels	X	X

^a^ Treven et al. (2015).

^b^ Dezsi et al. (2013).

^c^ Hassel et al. (2010).

^d^ Ueda et al. (2012).

^e^ Tran et al. (1999).

^f^ Comai et al. (2012b), Gallagher and Herrmann (2014), and Huband et al. (2010).

The major excitatory and inhibitory neurotransmitters are glutamate and GABA, respectively (Kandel et al., 2000), and these neurotransmitter systems are also targeted by many other AEDs (Table 2). Monoamines including serotonin (5-HT), dopamine (DA), and noradrenaline (NA) represent another group of neuroactive compounds that regulate neural activity and thus could influence the initiation and spread of seizure activity (Starr, 1996; Giorgi et al., 2004; Stefulj et al., 2010); however, these are not primarily targeted by any current AEDs.

### C. Neurobiology and Neuropharmacology of Aggression

**Fig. 1.**
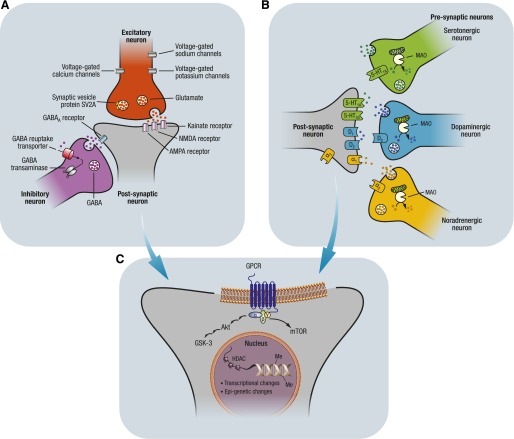
Schematic representation of brain targets common to the neurobiology and pharmacology of epilepsy and aggression. All of the targets illustrated in the figure are described in the main text. (A) Example of a prototypical excitatory (glutamate)/inhibitory (GABA) synapse modulating the activity of a forebrain postsynaptic neuron. Postsynaptic targets include glutamate (NMDA, AMPA, and kainite receptors) and GABA_A_ receptors. SV2A is a membrane glycoprotein that regulates neurotransmitter release from secretory vesicles. Voltage-gated K^+^, Na^+^, and Ca^2+^ channels modulate the action potential and resting membrane potential thus controlling neuronal firing activity. GABA transaminase catabolizes GABA into succinic semialdehyde. (B) Schematic representation of synaptic interactions between 5-HT, norepinephrine, and DA neurons with their projections to the forebrain postsynaptic neurons. On the presynaptic level, the autoreceptors mediating the inhibitory action of these three neurotransmitters are indicated. Notably, autoreceptors are targets for aggression treatment. The *α*_2_-adrenoceptor agonist clonidine is used to manage aggression, the D_2_ autoreceptor antagonist haloperidol is an antipsychotic with antiaggressive properties, and the 5-HT_1A_ autoreceptor agonist eltoprazine has demonstrated potent antiaggressive properties in preclinical studies. The enzyme MAO, which catabolizes the monoamines DA, norepinephrine, and 5-HT, is located on the outer membrane of mitochondria, and MAO inhibitors are widely used in mood disorders. On the postsynaptic level, the monoaminergic receptor subtypes implicated in the pathophysiology and pharmacology of epilepsy and aggression are indicated. (C) A schematic simplification of the Akt/GSK3 and mTOR signaling pathways is shown; these pathways are regulated by G protein–coupled receptors, which may be involved in the neurobiology and in the treatment of both aggression and seizures. Furthermore, processes in the nucleus have also been implicated in aggression and epilepsy: DNA transcription, and epigenetic modifications of DNA such as methylation of the C-5 position of the cytosine ring, and histone modification (deacetylation of histones by histone deacetylases). GSK3, glycogen synthase kinase 3; HDAC, histone deacetylase; Me, methylation; mTOR, mammalian target of rapamycin.

Aggression is a complex behavior governed by several cortical and subcortical brain networks that are modulated by neurotransmitter systems, including monoamines, glutamate, and GABA, and by ion channels (Gedye, 1989; Sumer et al., 2007). The neurobiological impairments observed in aggression occur at genomic and transcriptional levels as well as at the level of the synthesis and the metabolism of various neurotransmitters and their receptors. The main receptors and enzymes involved in the neurobiology of aggression, which are also targeted by antiaggressive medications, include those involved in monoamine neurotransmission–serotonin 5-HT_1A_ and 5-HT_2A_ receptors, 5-HT transporters, DA D_1_ and D_2_ receptors, DA transporters, *α*_1-_ and *α*_2_-adrenoceptors, the enzyme monoamine oxidase (MAO)-A, the GABA system (GABA_A_ and GABA_B_ receptors and GABA transaminase), the glutamate NMDA and AMPA receptors, and voltage-gated Na^+^ and Ca^2+^ channels (Siever, 2008; Comai et al., 2012a) (Fig. 1). Genetic and epigenetic regulation of these neurotransmitters, channels, and enzymes as well as the intracellular events after their cell activation/deactivation is also involved in aggressive behavior (Comai et al., 2012a). For example, polymorphic variation in the gene for MAO-A (Manuck et al., 2000) and MAO-A gene promoter hypermethylation, which causes a downregulation of MAO-A activity (Checknita et al., 2015), have been associated with aggressive behavior.

At a structural level, the study of brain lesions, brain injury, and imaging techniques has identified several regions as important in the pathophysiology of aggression, particularly the amygdala, hippocampus, and frontal lobes (Bannon et al., 2015). At a functional level, positron emission tomography and single-photon emission computed tomography imaging studies suggest that the ventromedial cortex, limbic system, amygdala, and thalamus are involved in impulsivity and aggression; in particular, reduced activity in the medial temporal subcortical lobe has been demonstrated (reviewed by Soyka, 2014). In the search for possible imaging biomarkers of aggression, a reduced gray matter volume at the level of the orbitofrontal cortex and low amygdala volume have both been linked with predisposition to violence (Matthies et al., 2012; Pardini et al., 2014; Bannon et al., 2015). Collectively, although brain imaging studies have revealed important information about the neuroanatomy underlying aggressive and impulsive behavior, this knowledge cannot yet be used to predict aggression in humans (Razafsha et al., 2015). In the future, imaging studies in patients with epilepsy in whom aggression develops or worsens after AED treatment may help identify brain abnormalities associated with epilepsy and aggression and may identify prognostic markers of aggression in patients with epilepsy.

### D. Networks and Neurotransmitters Common to Epilepsy and Aggression

EEG changes during seizures and their clinical manifestation reflect the localization of the seizure activity (Engel et al., 2007); subcortical structures (including the thalamus and brain stem) may play a crucial role in the propagation and behavioral manifestations of epileptic seizures rather than just being a site of seizure origin (Norden and Blumenfeld, 2002).

#### 1. Temporal Lobes and Hippocampus

One of the most common and studied forms of focal epilepsy is TLE. Seizures originate in the temporal lobes (either the inner surface/structures or more rarely the neocortex) and can be associated with psychiatric symptoms, including mood changes and aggressivity (Zhao et al., 2014). The temporal lobe is also important in aggression; in milestone research by Klüver and Bucy (1937), bilateral temporal lobectomy in rhesus monkeys reduced aggression. The temporal lobe is part of the limbic system that controls emotions and memory, and subjects with histories of extremely violent behavior have shown metabolic abnormalities in the temporal lobes (Seidenwurm et al., 1997); functional and/or structural abnormalities in neural networks regulating emotions have been related to an increased susceptibility for impulsive aggression and violence (Davidson et al., 2000). TLE is often associated with an extensive loss of dentate hilar neurons and hippocampal pyramidal cells—the so-called “hippocampal sclerosis” (Sloviter, 1994)—and interestingly, hippocampal pathology has been identified in pathologic aggression (as discussed later).

#### 2. Amygdala

The amygdala has been implicated in epilepsy, particularly in TLE and epileptogenesis, since the landmark studies by Feindel and Penfield (1954) and later by Goddard et al. (1969). Since this early work, many studies have shown that different forms of epilepsy are associated with damage to the amygdaloid complex (23 distinct subnuclei in humans) and to its connectivity to the surrounding brain regions and to the cortex (Pitkänen et al., 1998; Tebartz Van Elst et al., 2002; Takaya et al., 2014). The amygdala is also implicated in aggressive behavior: amygdalectomy in both animals and humans can stop aggressive behavior (Terzian and Ore, 1955), and stereotactic amygdalotomy of specific amygdaloid nuclei (e.g., the lateral or the anteromedial group) has been shown to control behavior in highly aggressive, treatment-refractory individuals (Mpakopoulou et al., 2008).

#### 3. Frontal Lobes

Frontal lobe epilepsy, the second most common type of focal epilepsy, is also associated with aggressive behavior during and after seizures (Gedye, 1989; Sumer et al., 2007). Damage to the frontal lobes is implicated in violent and aggressive behavior: patients with frontal ventromedial lesions consistently demonstrated higher rates of aggression/violence (especially verbal confrontations) than patients with lesions in other brain areas (Grafman et al., 1996). Motor agitation and aggressive behavior have also been shown in patients with orbitofrontal seizures (Tharp, 1972), further supporting the role of dysfunction in this brain region in aggression (Giancola, 1995).

#### 4. Hypothalamus

**Fig. 2.**
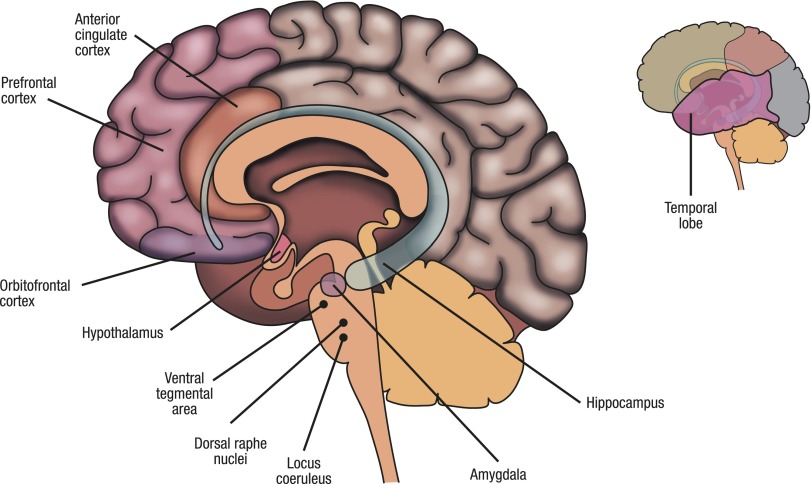
Brain regions that are important in both epilepsy and aggression. Schematic rendering of brain regions and nuclei involved in the neurobiology of both epilepsy and aggression. The role of each highlighted brain region/nuclei in epilepsy and aggression is discussed in the main text.

Other brain areas that are implicated in the pathophysiology of aggressive behavior and are components of emotional regulation circuits include the anterior cingulate cortex, amygdala, hypothalamus, septal nuclei, and periaqueductal gray matter of the midbrain (Fig. 2). The hypothalamus is an important part of the diencephalon that is implicated in both epilepsy and aggression. Studies in cats have shown that the hypothalamus, particularly the anterior medial hypothalamus, is involved in the modulation of defensive rage behavior (Gregg and Siegel, 2001). A positron emission tomography study in humans found low hypothalamic activity in male perpetrators of domestic violence and decreased correlations between cortical and subcortical brain structures (George et al., 2004). Anterior hippocampal asymmetries have been demonstrated in antisocial and violent subjects (Raine et al., 2004), and an inverse correlation was shown between hippocampal gray matter volume and lifetime aggression in borderline personality disorder (Zetzsche et al., 2007).

Taken together, it is clear that neurobiological networks that are important in epilepsy are also common neural substrates implicated in aggressive behavior. A summary of the networks and brain regions that are important in both epilepsy and aggression is shown in Fig. 2.

Within these brain regions and networks, the receptors and ion channels that are implicated in both epilepsy and aggression are also the targets of AEDs and mood stabilizers. In addition, alterations at the level of intracellular signaling cascades, gene sequence, or gene expression have been shown in both epilepsy and aggression in these areas.

#### 5. GABA

Clinical and preclinical studies have shown that seizures are liable to occur with an increase in glutamate and/or decrease in GABA neurotransmission in the brain. However, the changes in glutamate and/or GABA levels are different in different types of epilepsy, animal models, and brain regions (Engel et al., 2007). GABA_A_ receptors are ligand-gated chloride ion channels and are the major inhibitory receptors in the central nervous system (Olsen and Tobin, 1990). Houser et al. (2012) have extensively reviewed GABA_A_ receptor expression, subunit composition, and function in epilepsy. Changes in GABA_A_ receptor expression and function have been reported in animal models of TLE (Peng et al., 2004), and the composition and function of GABA_A_ receptors changes not only in epilepsy but also after prolonged exposure to GABA_A_ allosteric modulators such as the AEDs diazepam and phenobarbital (Raol et al., 2005).

GABA neurotransmission has been also been investigated in the pharmacology and pathophysiology of aggression (Comai et al., 2012a). The situation is complex, and there is no clear agreement on whether GABA levels are decreased or increased in aggression, or whether enhancing GABA is anti- or proaggressive. For example, Bjork et al. (2001) found a negative correlation between plasma GABA levels and aggressiveness in psychiatrically healthy people with a family history of depression, whereas Lee et al. (2009) reported a positive correlation between GABA levels in the cerebrospinal fluid and impulsivity (but not aggression) in both nonmedicated normal controls and subjects with personality disorder. Allosteric modulators of GABA_A_ receptors, including barbiturates and benzodiazepines, have been shown to influence aggression levels in rodents with an inverted U-shaped dose-response curve: moderate doses induce aggression, whereas low or high doses reduce aggressive behavior (Miczek et al., 2002).

#### 6. Glutamate

Glutamate is the principal excitatory neurotransmitter in the brain. It acts through ionotropic (NMDA, AMPA, and kainite) and metabotropic receptors and plays a significant role in the initiation, spread, and maintenance of epileptic activity. Research has demonstrated that epilepsy is linked to dysfunction of the glutamate system at different levels: genetic, neurotransmitter release, and receptor expression (Bradford, 1995; Barker-Haliski and White, 2015). Studies have reported increased plasma levels of glutamate in epilepsy (van Gelder et al., 1980; Janjua et al., 1992) and sustained increases in extracellular glutamate levels during seizures in the epileptogenic hippocampus (During and Spencer, 1993). Focal brain cooling, which can suppress epileptic seizures in refractory epilepsy, has been shown to significantly decrease glutamate levels in patients who have elevated glutamate in the cortex and/or hippocampus during seizures (Nomura et al., 2014). Glutamate is cleared from synapses by the membrane glutamate transporters and is loaded into synaptic vesicles by the vesicular glutamate transporters VGLUT1, VGLUT2, and VGLUT3 (El Mestikawy et al., 2011). A recent postmortem study comparing VGLUT expression in the hippocampus found significantly decreased VGLUT2 expression and significantly increased VGLUT3 expression in patients with TLE with hippocampal sclerosis, compared with autopsy controls (Van Liefferinge et al., 2015). Glutamate receptors, both ionotropic and metabotropic, have been extensively studied for their role in epilepsy in both human and animal studies (Moldrich et al., 2003; Ghasemi and Schachter, 2011; Szczurowska and Mareš, 2013). Glutamate receptors are targeted selectively and nonselectively by several AEDs (Table 2), and novel glutamate receptor ligands are also under development as potential AEDs (Löscher et al., 2013; Nicoletti et al., 2015).

Attention was initially directed toward antagonists of NMDA receptors, but they failed in several clinical trials (Rogawski, 2011). Interestingly, the NMDA antagonists phencyclidine (PCP) and ketamine have proconvulsant action at very high doses and anticonvulsant properties at low doses (Leccese et al., 1986). Research then moved to AMPA receptors (Rogawski, 2011), and 2012 saw the approval of the first selective AMPA receptor antagonist, perampanel, for the treatment of focal seizures. The role of kainate receptors in the pathophysiology of epilepsy is becoming better understood, particularly in TLE, and this was recently reviewed (Crépel and Mulle, 2015). Kainate receptors are located either presynaptically at both GABAergic and glutamatergic synapses, where they control neurotransmitter release and are involved in presynaptic plasticity (Schmitz et al., 2001), or postsynaptically in several regions deeply involved in epilepsy, including the cortex, hippocampus, and amygdala (Crépel and Mulle, 2015).

Few studies thus far have investigated the role of glutamate in aggression (Comai et al., 2012a); however, there is strong evidence of the involvement of glutamate in the pathophysiology and treatment of mood disorders such as depression (Kugaya and Sanacora, 2005; Niciu et al., 2014) that often coexist with aggressive behavior. Support for the glutamate hypothesis of aggression includes the demonstration of a positive relationship between cerebrospinal fluid glutamate levels and measures of impulsive aggression in both healthy human subjects and subjects with personality disorders (Coccaro et al., 2013). Studies in mice with genetic modifications at the levels of the genes encoding for the NMDA and AMPA receptors have shown altered levels of aggression in the resident intruder test (Brodkin et al., 2002; Duncan et al., 2004; Vekovischeva et al., 2004), an animal paradigm of aggressive behavior, suggesting a role for glutamate ionotropic receptors in aggression in rodents. In mice, the NMDA receptor channel blocker PCP produces a nonsignificant trend toward increased aggressiveness at low doses, whereas it seems to reduce aggression at high (near ataxic) doses (Belozertseva and Bespalov, 1999). The fact that medications that can decrease aggression (e.g., valproic acid and topiramate) have inhibitory effects at NMDA and AMPA receptors (Comai et al., 2012b) provides further supportive evidence for the involvement of glutamate in aggression; however, the association of these same AEDs with aggression in some patients with epilepsy shows that the mechanisms are complex.

#### 7. Serotonin

Bonnycastle et al. (1957) were the first to demonstrate a link between anticonvulsant activity and 5-HT, showing that several AEDs, including phenytoin, significantly increased 5-HT levels. Pharmacological agents that elevate 5-HT levels (e.g., the selective serotonin reuptake inhibitor fluoxetine and 5-hydroxytryptophan) have anticonvulsant effects in several animal models of epilepsy (Pasini et al., 1992), whereas agents that reduce and/or deplete 5-HT (e.g., parachlorophenylalanine, a selective and irreversible inhibitor of tryptophan 5-hydroxylase) increase susceptibility to sound-induced seizures (Schlesinger et al., 1968). 5-HT receptors are expressed in almost all networks involved in epilepsy. With the exception of 5-HT_5_ receptors, for which there is no evidence yet of involvement in epilepsy, Gharedaghi et al. (2014) recently reviewed and commented on the role of each 5-HT receptor subtype in epilepsy and seizure susceptibility.

Activation of 5-HT_1A_ receptors is anticonvulsant in various experimental seizure models. A study administering a 5-HT_1A_ agonist in lithium-pilocarpine–induced status epilepticus in mice showed that hippocampal 5-HT_1A_ receptors are involved in reducing seizure severity, whereas those located in extrahippocampal areas contribute to delayed seizure propagation (Yang et al., 2014). In patients with TLE, decreased 5-HT_1A_ receptor binding has been observed in the midbrain raphe, the ipsilateral thalamus, and the inferior region of the epileptogenic temporal lobe (Toczek et al., 2003). Genetic inactivation of 5-HT_2C_ receptors in mice produces rare spontaneous seizures that are occasionally fatal (Heisler et al., 1998).

5-HT has a central role in aggression and hundreds of articles have examined this topic since the early 1960s. It was believed that 5-HT generally inhibits aggression but recent findings challenge this hypothesis, showing that this may be limited to certain types of aggression, such as impulsive aggression, or perhaps instead to factors such as impulse control and emotion regulation (Krakowski, 2003). For example, the main metabolite of 5-HT, 5-hydroxyindoleacetic acid, is reduced in the cerebrospinal fluid of people with demonstrated auto- or heteroaggressive behavior compared with people who have never shown such behavior. These observations were made in suicidal individuals (Asberg et al., 1976), in nondepressed men with a history with aggressive behavior (Brown et al., 1979), violent men in prison (Linnoila et al., 1983), in impulsive arsonists (Virkkunen et al., 1987), and in patients with a personality disorder (Coccaro et al., 1989). The 5-HT_1A/1B_ agonist eltoprazine is considered a potent antiaggressive agent (Comai et al., 2012a).

#### 8. Dopamine

The role of DA in epilepsy is still debated, although there is evidence of dopaminergic system involvement in certain animal models of epilepsy and in various forms of epilepsy in humans (Starr, 1996). In particular, alterations of subcortical dopaminergic pathways may be specifically related to the motor manifestations of certain types of seizures (Norden and Blumenfeld, 2002).

In general, DA seems to exert an antiepileptic action, as demonstrated by the fact that the nonselective D_1_/D_2_ agonist apomorphine, with certain limitations, has anticonvulsant properties, whereas neuroleptic drugs that act as D_1_ and/or D_2_ antagonists have predominantly proconvulsant actions. This is important, because some medications used to control aggression are D_1_ and/or D_2_ antagonists (Comai et al., 2012b). Looking at the role of specific DA receptors in epilepsy, evidence from both animal (Turski et al., 1988; al-Tajir et al., 1990) and human (Ring et al., 1994) studies has implicated D_2_ receptors.

The dopaminergic system is mainly implicated in behavioral activation, motivated behavior, and reward processing (Ikemoto and Panksepp, 1999), but evidence suggests that it also modulates aggressive behavior. Cerebrospinal fluid levels of homovanillic acid, the final metabolite of DA, are lower in impulsively aggressive violent offenders with antisocial personality disorder than in nonimpulsively aggressive offenders with paranoid or passive-aggressive personality disorder (Linnoila et al., 1983). DA receptor antagonists (particularly conventional antipsychotics that target D_2_ receptors, such as haloperidol) have been used effectively for decades to treat aggression in psychotic patients. Even though DA has been hypothesized to be necessary for aggressive behavior to occur, reflecting the motivational aspect of violence (Nelson and Chiavegatto, 2001; de Almeida et al., 2005), the exact role of DA in aggression is still unclear (Comai et al., 2012a).

#### 9. Noradrenaline

Endogenous NA seems to be generally antiepileptic: NA levels are decreased after seizures, depending on the seizure type and brain region (Giorgi et al., 2004), and AEDs such as valproic acid increase NA levels in the rat (Baf et al., 1994). The antiepileptic actions of NA are very likely mediated by both *α*_1_- and *α*_2_-adrenoceptors; indeed, *α*_2_-adrenoreceptor agonists have been shown to suppress, and *α*_2_-adrenoreceptor antagonists to promote, seizures in kittens (Shouse et al., 2007).

In aggression, NA seems to play a permissive role, helping to determine whether an individual elects to fight or flee in response to a challenge (Miczek and Fish, 2005). Studies have explored the effects of an experimental depletion or increase of NA levels, as well as activation or inhibition of NA receptors, on aggression. However, research is still limited and no clear positive or negative correlation has yet been demonstrated. Some studies report reduced fighting tendency after depletion of brain NA in male mice with isolation-induced fighting as well as after intraventricular injection of 6-hydroxydopamine (Crawley and Contrera, 1976). Some studies, in contrast, report increased aggression (shock-induced fighting) after NA depletion in rats (Thoa et al., 1972). The role of *α*_2_-adrenoceptors is important in mediating the effects of NA manipulations on aggression. For example, increasing NA levels with desipramine (a NA reuptake blocker) increases isolation-induced aggression in mice in a dose-dependent manner, and the *α*_2_-adrenoceptor blocker yohimbine dose-dependently counters this increase (Matsumoto et al., 1991). In addition, the *α*_2_-adrenoceptor agonist clonidine, which decreases NA neuronal activity by activating NA autoreceptors, has been shown to decrease pathologic aggression in humans and is used in clinical settings in irritable autistic children, children with conduct disorder (Fava, 1997), and also in adults with aggression (Comai et al., 2012b). Clonidine is also of value in treating ADHD in children, particularly those with conduct or oppositional defiant disorder (Connor et al., 1999, 2000).

#### 10. Intracellular Signaling Cascades, Genes, and Epigenetic Gene Regulation in Epilepsy and Aggression

The role of intracellular events (e.g., intracellular proteins and signaling cascades, genes and epigenetic modifications) is another important framework in which to consider in the neurobiology of epilepsy and AED development (Löscher et al., 2013).

The study of intracellular cascade events after receptor activation has been actively researched in epilepsy. In the pilocarpine-induced status epilepticus model, for example, increased phosphorylation of the mitogen-activated protein kinases (MAPKs) extracellular signal-regulated kinase (ERK)-1, ERK2, and p38^MAPK^ has been demonstrated in the acute period (up to 12 hours after status epilepticus) and protein kinase B in the latent period (5 days after pilocarpine-induced status epilepticus) (Lopes et al., 2012). These biochemical changes in the serine-threonine kinases, which are implicated in neuronal survival and differentiation as well as neuroplasticity, may in turn alter gene expression and produce long-lasting neuronal changes. However, ERK signaling has multiple roles during epilepsy-related processes; its activation can contribute to acute seizure activity and might be necessary for epileptiform synchronization (Houser et al., 2008). The mammalian target of rapamycin is a regulator of mRNA translation that is itself regulated by ERK and protein kinase B (Li et al., 2010). As reviewed by Cho (2011), there is evidence to implicate abnormal activity of signaling molecules in the mammalian target of rapamycin pathway in epilepsy.

There has been less research on the intracellular signaling cascades underlying aggressive behavior. However, because these cascades (and the G protein–coupled receptors that activate them) are targeted by medications used to treat aggression (Beaulieu, 2012), it is reasonable to hypothesize their active involvement in the pathophysiology of aggression (Comai et al., 2012a). Aggressive behaviors are common among abusers of methamphetamine, and repeated injections of methamphetamine in mice have also been shown to increase aggressive behavior (Sokolov and Cadet, 2006). In these animals, there were significant alterations in the expression of proteins involved in MAPK-related pathways in the striatum and frontal cortex (Sokolov and Cadet, 2006).

Regarding the specific involvement of genes in both epilepsy/seizure susceptibility and aggressive behavior, studies have been very limited in number. Two genes that have been implicated in both seizures and aggression are the gene encoding the MAO-A enzyme and that encoding the 5HT_1B_ receptor. Complex changes in seizure susceptibility are seen in MAO-A knockout mice, which have high levels of 5-HT and NA. After pentylenetetrazol kindling, the latency to seizure development is shorter in knockout mice than in wild-type mice; however, the knockout mice then have fewer seizures per day and shorter-duration seizures compared with wild-type mice, suggesting some protective effect despite the increased susceptibility to kindling (Teskey et al., 2004). Regarding aggressive behaviors, MAO-A knockout mice are more aggressive than wild-type mice, displaying decreased latency to attack in the resident intruder test (Cases et al., 1995). The only study in humans that explored links between epilepsy and polymorphisms in the gene encoding MAO-A was negative, but the study was underpowered to detect minor differences (Stefulj et al., 2010). The same study instead showed a modest association between the G861C polymorphism in the 5-HT_1B_ receptor gene and TLE. Notably, a 5-HT_1B_ polymorphism has been associated with suicide history and personality disorder in humans (New et al., 2001) and 5-HT_1B_ knockout mice display increased levels of aggression in the resident intruder test (Bouwknecht et al., 2001).

Epigenetic factors, including seizures themselves and AEDs, can alter neural network dynamics by interfering with signaling pathways and enzyme and receptor expression. Epilepsy can be influenced not only by alterations in genetic and environmental factors but also by a spectrum of dysfunctional epigenetic factors and processes (Qureshi and Mehler, 2010; Kobow and Blümcke, 2014). Indeed, hundreds of misregulated genes have been identified in human and experimental models of epilepsy after epigenetic chromatin modifications and DNA methylation. Modifications, such as DNA methylation, may accumulate over time after an initial injury, and a methylation hypothesis of epilepsy has been proposed (Kobow and Blümcke, 2012); such epigenetic changes may explain interindividual differences in the emergence of epilepsy, such as after brain trauma (Machnes et al., 2013). Interestingly, the AED valproic acid has been shown to inhibit histone deacetylases and thereby normalize expression of histone deacetylase–dependent genes within the epileptic dentate area in rats with kainic acid–induced seizures (Jessberger et al., 2007).

The epigenetic modifications involved in aggressive behavior have not been well studied. Evidence thus far demonstrates a relationship between epigenetic modifications in the 5-HT system and aggression. In a recent study, we found decreased MAO-A activity in antisocial offenders, resulting from epigenetic modifications in MAO-A gene methylation levels (Checknita et al., 2015). Methylation levels of the 5-HT transporter gene promoter (SLC6A4) were also shown to be altered in individuals displaying physical aggression during childhood (Wang et al., 2012).

### E. Pharmacological Targets of Antiepileptic Drugs for Epilepsy and Aggression

The pathophysiology of epilepsy is multifaceted, involving several neurotransmitter systems and many receptors, ion channels, intracellular signaling cascades, genes, and epigenetic modifications (Engel et al., 2007; Scharfman, 2007; Moshé et al., 2015). Several of these possible mechanisms are targeted by current AEDs and are being targeted by new drug development. As shown in Table 2, the majority of current AEDs target primarily either the classic voltage-gated ion channels (Na^+^, K^+^, and Ca^2+^) or the GABA system, whereas many AEDs have mixed mechanisms and others selectively inhibit other targets (e.g., neurotransmitter release by levetiracetam, AMPA receptors by perampanel) (Rogawski and Löscher, 2004; Brodie, 2010; Comai et al., 2012b).

Modulation of genes and epigenetic mechanisms by AEDs has also been reported in recent years (Comai et al., 2012a,b). Levetiracetam has a clear and distinct mechanism of action; it acts on the SV2A integral membrane glycoprotein present on all synaptic vesicles (Lynch et al., 2004). Recent work by Vogl et al. (2015) in superior cervical ganglion neurons demonstrated that SV2A maintained normal neurotransmission by regulating readily releasable pool size, had a facilitatory role in recovery from synaptic depression, and impaired presynaptic voltage-dependent Ca^2+^ channel current density. Specific details on the mechanisms of action of AEDs at the level of receptors and ion channels have been extensively reviewed (Kwan et al., 2001; Löscher et al., 2013; Moshé et al., 2015).

Many AEDs are also used to treat aggression in psychiatric populations, such as patients with schizophrenia, schizoaffective disorders, bipolar disorders, and autism spectrum disorders (Comai et al., 2012b). AEDs such as valproic acid, topiramate, gabapentin, and lamotrigine are frequently used, as are those targeting ion channels, including carbamazepine, oxcarbazepine, lamotrigine, and phenytoin (Comai et al., 2012b) (Table 2).

The atypical antipsychotics clozapine, olanzapine, quetiapine, and risperidone as well as the conventional antipsychotic haloperidol, which are high-affinity antagonists at 5-HT_2A_ and/or 5-HT_1A_ receptors and D_2_ receptor antagonists, are also widely used “off-label” to attenuate aggressive behavior (Gobbi et al., 1999; Comai et al., 2012b). The rationale derives from the abnormalities of 5-HT and DA systems in the pathophysiology of aggressive behavior (Comai et al., 2012a). Clozapine has been demonstrated to be superior to olanzapine and haloperidol in the control of aggression (Volavka et al., 2004). In more resistant cases, the combination of clozapine and an AED such as valproic acid remains the most effective treatment (Gobbi and Debonnel, 2003).

### F. Conclusions: Why Might Antiepileptic Drugs Induce Aggressive Behavior in Epilepsy?

There is a complex comorbidity between aggression and epilepsy and as we have shown in this section, aggression and epilepsy also have complex and overlapping pathophysiology. In the following sections, the evidence for a link between aggressive behavior and AEDs in people with epilepsy is reviewed. A critical question is, therefore, how can an AED apparently precipitate aggression in some people with epilepsy but improve aggressive behaviors in other people? Here, we discuss some speculations and hypotheses on this topic, based on common neurobiological correlates between epilepsy and aggression.

#### 1. Paradoxical Proaggressive Effects of Enhancing GABA Neurotransmission

The GABA system has the potential for both inhibitory and excitatory actions. In the neonatal brain, GABA is excitatory and not inhibitory, due to the high intracellular concentration of chloride (Ben-Ari and Holmes, 2006). In the mature brain, rhythmic afterdischarge of pyramidal CA1 cells after cessation of the stimulus relies on a powerful GABA_A_-mediated excitation mechanism (Fujiwara-Tsukamoto et al., 2003). In adults with TLE, the morphology, hippocampal expression, and subcellular distribution of GABA_A_ receptor subunits is markedly altered (Loup et al., 2000) and, in mature neurons, recurrent and prolonged seizures may trigger a pathologic re-emergence of immature excitatory features of GABA_A_ receptors (Galanopoulou, 2008). Moreover, TLE is often characterized by hippocampal sclerosis and thus depletion of GABAergic interneurons and glutamatergic hilar mossy cells, by mossy fiber (with colocalization of GABA and glutamate receptors) sprouting and synaptic reorganization in the dentate gyrus (Jinde et al., 2013). We hypothesize that in the epileptic brain, many modifications in GABA receptors and GABA-ergic neurons can occur, such that agents enhancing GABA neurotransmission (which decrease aggressive behaviors in subjects without epilepsy) can, depending on the brain region, have the opposite effect of triggering, rather than decreasing, aggression.

#### 2. Dose-Dependent and Opposite Effects of NMDA Receptor Antagonists on Epilepsy and Aggression

The glutamatergic system, like the GABA system, may have dose-dependent opposite effects. Depending on the dose, NMDA receptor blockers such as PCP appear to have both pro- and antiepileptic as well as pro- and antiaggressive properties (Leccese et al., 1986; Belozertseva and Bespalov, 1999). In particular, PCP at low doses is antiepileptic but proaggressive, whereas the opposite is observed at high doses. Increased aggression might consequently occur when AEDs that have an NMDA receptor–inhibitory component to their action are used at doses that have minimal NMDA receptor antagonism. Some subtypes of epilepsy, such as TLE, can be associated with a reshaping of glutamatergic neurons and receptors in the hippocampus; consequently, drugs blocking glutamatergic receptors may have a different or opposing effect in the epileptic brain than in nonepileptic brains.

#### 3. Genetic Predisposition and Decreased Dopaminergic Activity

A recent association study set out to explore whether there was a genetic basis that predisposes some patients to develop behavioral AEs during levetiracetam treatment. Helmstaedter et al. (2013) identified several polymorphisms, all of which were associated with reduced dopaminergic activity (variations in DA-*β*-hydroxylase, catechol-*O*-methyltransferase, and an intracellular D_2_-receptor binding protein), which seem to predispose patients with epilepsy treated with levetiracetam to develop aggression. It is not yet known whether this result is specific to levetiracetam, or whether reduced dopaminergic activity will predispose patients with epilepsy to behavioral AEs with other AEDs

#### 4. Forced Normalization

Finally, the sometimes controversial forced normalization theory is an attempt to explain the occasional observation of a paradoxical inverse relationship between epileptiform abnormality in EEG and psychiatric symptoms. It was first described in 1953 by Heinrich Landolt, who observed that EEGs paradoxically normalized and seizure activity was inhibited during psychotic episodes (Landolt, 1953). The forced normalization phenomenon is similar to but distinct from the concept of the “alternative psychosis” or “reciprocal psychosis” (that psychosis is better when seizures are worse and psychosis is worse when seizures are better/controlled, as discussed later). This hypothesis was supported by epidemiologic studies that found lower seizure frequency in patients with epilepsy and psychosis, and studies reporting relatively few cases involving comorbid schizophrenia and epilepsy (Schmitz and Trimble, 1992). This observation can be extended to psychiatric phenomena other than psychosis, such as hypomania/mania, aggression, depression, and anxiety (Trimble and Schmitz, 1998). Therefore, the psychiatric adverse effects seen during AED treatment may not be a direct adverse psychotropic effect of the AED, but rather a consequence of the suppression of seizures. This inverse link between seizure control and psychiatric symptoms (Flor-Henry, 1983) deserves further research, because it has far-reaching implications. Studies are needed to determine whether psychiatric symptoms seen with AEDs in patients with epilepsy are side effects with no prognostic or clinical value, whether they are a necessary consequence of seizure control, or whether they could be a stage of a complex and progressive phenomenon leading to a chronic psychiatric disorder (Mula et al., 2007). This is an important distinction, and future research should focus on clarifying the complex relationships between seizures and aggressive behavior.

## V. Antiepileptic Drugs and Aggression in Adult Patients with Epilepsy

### A. Introduction

We screened the literature for articles containing references to AED treatment and aggression-related behavior (aggression, agitation, anger, assault, homicide/homicidality, hostility, impulsivity, irritability) and selected relevant reports in epilepsy populations (see the Supplemental Material for the search strategy and evaluation). Supplemental Table 1 provides the full listing of relevant studies retrieved for each AED and a brief data summary. Relevant information from previous systematic reviews of individual drugs or of AEDs as a class has been included where relevant. We also reviewed product labels for information about aggression-related behaviors, as an up-to-date source of relevant adverse reactions in both clinical studies and postmarketing experience (Table 3).

**TABLE 3:** Summary of aggression-related behavior incidence and warnings, from AED labels AEDs are listed in alphabetical order.

AED	Aggression-Related AEs (and Incidence)	Other Related Text, Warnings, or Precautions	Epilepsy Specific?	Source
Carbamazepine	Aggression, agitation (rare: 0.01%–0.1%)	“The possibility of activation of a latent psychosis and, in elderly patients, of confusion and agitation should be borne in mind”	No	Tegretol (2014a,b)
				
Clobazam		“Aggressive behaviour towards self and others may be precipitated”	No	Frisium (2014), Onfi (2014)
	Irritability (7% with clobazam versus 5% with placebo)		Yes (LGS)	Onfi (2014)
	Aggression (8% with clobazam versus 5%)			
	Aggression (1 of the 6 AEs leading to clobazam discontinuation)			
				
Clonazepam	No incidence reported for aggression-related AEs	“Clonazepam generally has a beneficial effect on behaviour disturbances in epileptic patients. In certain cases, paradoxical effects such as aggressiveness, excitability, nervousness, hostility, anxiety, sleep disturbances, nightmares, vivid dreams, irritability, agitation, psychotic disorders and activation of new types of seizures may be precipitated”	Yes	Clonazepam (2014)
	No incidence reported for aggression-related AEs	“Behaviour problems have been noted in approximately 25% of patients”	Yes	Klonopin (2013)
				
Eslicarbazepine	Agitation, irritability (uncommon: 0.1%–1%)		Yes	Zebinix (2014)
				
Ethosuximide	Aggression, irritability (uncommon: 0.1%–1%), psychiatric AEs are seen “particularly in patients who have previously exhibited psychological abnormalities”		Yes	Zarontin (2012, 2014)
				
Felbamate	Aggressive behavior, agitation (common: ≥1%)		Yes	Felbatol (2012)
				
Gabapentin	Hostility (common: 1%–10%)		Yes	Neurontin (2013a,b)
	In children, aggressive behavior is also common (1%–10%)			
				
Lacosamide	Irritability (common: 1%–10%)		Yes	Vimpat (2014a,b)
	Aggression, agitation (uncommon: 0.1%–1%)			
				
Lamotrigine	Aggression, irritability (common: 1%–10%)		No	Lamictal (2014a)
	Irritability (3% versus 2% with placebo, adjunctive use)		Yes	Lamictal (2014b)
	Irritability (2%–5%, monotherapy)			
				
Levetiracetam	Hostility/aggression, nervousness/irritability (common: 1%–10%)		Yes	Keppra (2010)
	Anger, agitation (uncommon: 0.1%–1%)			
	Behavioral AEs were more common in children/adolescents than adults: agitation (3.4%), aggression (8.2%), irritability (11.7%, children aged <4 yr)			
	Nonpsychotic behavioral symptoms (levetiracetam versus placebo):	Warnings and precautions: “Behavioral abnormalities including psychotic symptoms, suicidal ideation, irritability, and aggressive behavior have been observed. Monitor patients for psychiatric signs and symptoms”	Yes	Keppra (2014)
	Adults (13.3% versus 6.2%)			
	Pediatric (37.6% versus 18.6%)			
Oxcarbazepine	Agitation (1%–2% versus 1% with placebo)		Yes	Trileptal (2013, 2014)
				
Perampanel	Adjunctive perampanel, occurrence in epilepsy clinical trials: aggression, anger, irritability (common: 1%–10%)	“Aggressive and hostile behaviour has been reported in patients receiving perampanel therapy. In perampanel-treated patients in clinical trials, aggression, anger and irritability were reported more frequently at higher doses. Most of the reported events were either mild or moderate and patients recovered either spontaneously or with dose adjustment. However, thoughts of harming others, physical assault or threatening behaviour were observed in some patients (<1% in perampanel clinical studies)”	Yes	Fycompa (2015)
	Adjunctive perampanel, occurrence with 12 mg versus placebo in epilepsy clinical trials:	“Serious or life-threatening psychiatric and behavioral adverse reactions including aggression, hostility, irritability, anger, and homicidal ideation and threats have been reported in patients taking FYCOMPA. These reactions occurred in patients with and without prior psychiatric history, prior aggressive behaviour, or concomitant use of medications associated with hostility and aggression”	Yes	Fycompa (2014)
	Irritability: 12% versus 3%			
	Aggression: 3% versus 1%			
	Anger: 3% versus <1%			
	Total incidence of hostility- and aggression-related adverse reactions: 20% with 12 mg, 12% with 8 mg, 6% with placebo			
				
Phenobarbital	AE rates are not given. The listing of undesirable effects includes: “paradoxical reaction (unusual excitement)” and “behavioural disturbances in children”		Yes	Phenobarbital (2013)
				
Phenytoin	No mention of aggression-related behavior in the SPC or USPI			
				
Pregabalin	Irritability (common: 1%–10%)		No	Lyrica (2014, 2015)
	Agitation, aggression, hostility (uncommon: 0.1%–1%)			
				
Retigabine	Aggression-related behaviors reported only in overdose		Yes	Potiga (2013), Trobalt (2014)
				
Rufinamide	No aggression-related behaviors or adverse reactions are listed in the SPC		Yes	Inovelon (2013)
	Aggression (3% versus 2% with placebo)		Yes	Banzel (2015)
				
Stiripentol	Aggressiveness, irritability (common: 1%–10%)		Yes (children)	Diacomit (2014)
				
Tiagabine	Hostility (2%–5% versus 1%–2% with placebo)		Yes	Gabitril (2010, 2014)
	Agitation (1% versus 0%)			
				
Topiramate	Irritability (>5%)	Aggression was more common in children than in adults	Mostly*^a^*	Topamax (2013)
	Aggression, agitation, anger, abnormal behavior, irritability (common: 1%–10%)			
	Aggression, agitation (3% versus 2% with placebo, clinical study in adults)		Yes (individual epilepsy clinical trial data)	Topamax (2014)
	Aggression (2% versus 0% with placebo, clinical study in adults)			
	Aggression (9% versus 4%, pediatric trials)			
				
Valproic acid	Aggression, agitation (common: 1%–10%), predominantly in children		Yes	Epilim (2013)
	No terms of interest are listed in the epilepsy clinical study tables in the USPI		Yes	Depakene (2015)
	Aggression, hostility, and irritability are listed as adverse reactions reported in postmarketing experience (no incidence given)			
				
Vigabatrin	Pediatric: Agitation (very common: ≥10%)	“Vigabatrin should be used with caution in patients with a history of psychosis, depression or behavioural problems. Psychiatric events (e.g., agitation, depression, abnormal thinking, paranoid reactions) have been reported during vigabatrin treatment. These events occurred in patients with and without a psychiatric history, and were usually reversible when vigabatrin doses were reduced or gradually discontinued”	Yes	(Sabril, 2014)
	Adults: aggression, agitation, and irritability (common: 1%–10%)			
	Adults: irritability (23% with 6000 mg versus 7% with placebo)		Yes	Sabril (2013)
	Adolescents: aggression (5% versus 0% with placebo)			
	Infantile spasm: irritability (16%–23%)			
				
Zonisamide	Adjunctive use:		Yes	Zonegran (2014)
	Agitation, irritability (very common: >10%)			
	Anger, aggression (uncommon, 0.1%–1%)			
	Monotherapy:			
	Agitation (common: 1%–10%); aggression (uncommon)			
	Adjunctive use:		Yes	Zonegran (2012)
	Agitation/irritability (9% versus 4% with placebo)			

LGS, Lennox–Gastaut syndrome; SPC, summary of product characteristics; USPI, US prescribing information.

^a^ Predominantly epilepsy studies, but a minority of studies in migraine prophylaxis.

Our literature search and analysis captures only formally reported events of aggressive behavior and aims to identify large-scale, consistent patterns. As clinicians, we all have anecdotal experience of aberrant behaviors that appear in an individual patient after the addition of an AED and resolve on its removal. We have experienced this with all AEDs, including those that have no formal reports of aggression. Every patient is an individual, and each subtype of epilepsy is different; although we have identified some broad trends, we acknowledge that aberrant aggressive behavior may develop in the occasional individual with an AED that is not usually associated with such behavior. Additional confounding factors that may result in incorrect attribution of behavioral disturbance to a specific AED are discussed elsewhere in this article and in previous publications (Besag, 2001).

### B. Review of Data for Each Antiepileptic Drug

The majority of studies we retrieved focused on one AED. Those that included a number of AEDs are discussed separately under each AED heading. One study included sufficient patients and AEDs to allow some comparisons of psychiatric side effects between the AEDs and merits discussion here. A retrospective chart review was conducted of adult outpatients seen between 2000 and 2005 at a US epilepsy center and included 1394 patients who had taken one of the following newer AEDs (at the time): gabapentin, lamotrigine, levetiracetam, topiramate, tiagabine, vigabatrin, and zonisamide (Weintraub et al., 2007). Overall, an average of 8.4% of patients experienced AED-related psychiatric/behavioral side effects. The highest rates were seen with levetiracetam (16% incidence and 8% discontinuations, significantly higher than average, *P* < 0.001) and tiagabine (not significant, probably due to low patient numbers); intermediate rates were seen with topiramate and zonisamide and low rates were seen with gabapentin (0.6% incidence, significantly lower than average, *P* < 0.001), lamotrigine (4.8% incidence, *P* < 0.001), and vigabatrin, felbamate, and oxcarbazepine (not significant, probably due to low patient numbers). A past psychiatric history was the most significant predictor of AED-related behavioral/psychiatric side effects (Weintraub et al., 2007).

#### 1. Brivaracetam

Brivaracetam is included in this article because it is currently under review for approval as an adjunctive treatment of focal epilepsy with or without secondary generalization. Our searches retrieved no published studies with brivaracetam that included any of our key search terms. Manual searches for brivaracetam randomized controlled trials (RCTs) identified three phase III studies (Biton et al., 2014a; Kwan et al., 2014; Ryvlin et al., 2014) and two phase IIb studies (French et al., 2010; Van Paesschen et al., 2013) that reported AEs (Supplemental Table 1). Irritability was seen in approximately 5% of patients with 50 mg/d (versus 2%–4% with placebo) in three of the four studies (Van Paesschen et al., 2013; Biton et al., 2014a; Ryvlin et al., 2014). Discontinuation of brivaracetam was most often prompted by psychiatric AEs (including aggression and irritability) in two studies (Biton et al., 2014a; Ryvlin et al., 2014). A post hoc meta-analysis across these phase II/III studies reported that 6.8% of 1214 brivaracetam-treated patients had nonpsychotic behavioral AEs, compared with 4.2% in the placebo group (n = 425). This incidence is lower than across the phase III studies with levetiracetam [10.9% (n = 672) versus 4.8% with placebo (n = 351)] (D’Souza et al., 2012).

#### 2. Carbamazepine

One RCT with behavior-specific measures was found. This study used a withdrawal design to show that compared with the group that continued AEDs, there was a small improvement in depression and in “brooding” after withdrawal of AED in seizure-free patients with epilepsy treated with AEDs (both overall and in the carbamazepine subgroup) (Hessen et al., 2007), suggesting that carbamazepine may negatively affect mood. No measures directly related to aggression (e.g., irritability, anger) were reported. However, anecdotal experience of this review’s authors suggests that carbamazepine may have mood-stabilizing properties in some patients with epilepsy; we have observed deterioration in mood after withdrawal of carbamazepine.

No carbamazepine RCTs that reported aggression-related behavioral AEs were retrieved by our searches.

Four observational studies were retrieved: two showed slight evidence of aggression-related behaviors with carbamazepine in patients with epilepsy (Shehata et al., 2009; Wieshmann et al., 2011), one showed positive behavioral effects (Pulliainen and Jokelainen, 1994), and one showed no behavioral worsening (Friedman et al., 1992) (Supplemental Table 1). Using the Liverpool Adverse Event Profile to assess patient-reported AEs, one study reported that 16% of the 36 patients taking carbamazepine had feelings of anger (compared with 33% for levetiracetam, 19% for valproic acid, and 15% for lamotrigine) (Wieshmann et al., 2011). One study showed that patients with epilepsy performed worse on several behavioral-specific measures, compared with healthy controls, and that behavior worsened in relation to dose and duration of AED treatment (with carbamazepine or valproic acid) (Shehata et al., 2009). A partially blinded, prospective, observational study that focused mainly on cognitive outcomes showed a slight improvement from baseline in negative mood after 6 months of phenytoin or carbamazepine (Pulliainen and Jokelainen, 1994). A small observational study in patients with developmental delay showed no increase in behavioral problems in patients receiving carbamazepine as an AED only, but worsening behavior was reported in patients receiving carbamazepine for either comorbid seizures and behavioral/psychiatric disorder or for psychiatric disorders alone (Friedman et al., 1992).

#### 3. Clobazam

No relevant studies in adult populations were retrieved.

#### 4. Clonazepam

Our searches identified no RCTs for clonazepam in adults, either using behavior-specific endpoints or AE reporting.

One observational study (published in 1979) with clonazepam in 40 patients aged 8–60 years with generalized or focal seizures reported irritability as an AE in 7 patients (17%) (Lander et al., 1979). Edwards (1974) reported “irritability or aggressiveness” in 5%–20% of patients in previous studies and “frank aggressiveness” requiring dose reduction in 20% of patients in that author’s personal experience.

#### 5. Eslicarbazepine Acetate

Our searches (including abstracts at key epilepsy congresses; see the Supplemental Material) identified no RCTs or observational studies that reported aggression-related data with eslicarbazepine acetate in patients with epilepsy. One conference abstract on the incidence of psychiatric AEs with eslicarbazepine acetate did not mention any aggression-related AEs (Biton et al., 2014b).

#### 6. Ethosuximide

Our searches found no studies reporting aggression-related behaviors in adults with epilepsy treated with ethosuximide; however, psychosis has been reported. For example, Fischer et al. (1965) reported five episodes of psychosis in three adults treated with ethosuximide; although no aggression was reported in any of these cases, the patients were described as being “tense.” The psychosis occurred in clear time relation to the ethosuximide treatment; the authors suggested that this might have been a manifestation of forced normalization.

#### 7. Felbamate

Our searches identified no RCTs with behavior-specific endpoints. Rates of psychiatric/behavioral AEs were low with felbamate in the large observational study discussed in section V.B (Weintraub et al., 2007). One observational study of headache in 60 patients with epilepsy taking felbamate reported adverse effects of “agitation or restlessness” in 14 patients (23%), which caused discontinuation in 6 patients (12%) (Ettinger et al., 1996). A case series of seven patients with epilepsy with behavioral changes with add-on felbamate reported that the changes included marked agitation/irritability in four patients and that the changes resolved on discontinuation in all seven patients (McConnell et al., 1996) (Supplemental Table 1).

#### 8. Gabapentin

Our searches identified no relevant studies with gabapentin in adults with epilepsy, other than the large observational study mentioned earlier (section V.B); rates of psychiatric/behavioral AEs were significantly lower with gabapentin (0.6% of 160 patients) than the average (*P* < 0.001) (Weintraub et al., 2007).

#### 9. Lacosamide

Our searches identified no relevant studies with lacosamide in adults with epilepsy. A manual search of lacosamide RCTs found no mention of aggression-related behaviors as AEs in individual epilepsy trials, in which the threshold for reporting AEs ranged from ≥5% to ≥10%, in a pooled analysis of lacosamide epilepsy trials (Sake et al., 2010) or in a meta-analysis of AE data across 10 RCTs in epilepsy and other indications (Zaccara et al., 2013).

#### 10. Lamotrigine

Our searches identified one RCT with behavior-specific endpoints. In adults with focal seizures treated with adjunctive lamotrigine or levetiracetam, anger and hostility improved (i.e., lessened) from baseline with lamotrigine, with a significant difference versus levetiracetam, based on anger-hostility subscale scores of the Profile of Mood States (POMS) scale (Labiner et al., 2009).

Two observational studies designed to look at behavior present good evidence that lamotrigine is not typically associated with behavioral/aggressive side effects. The large observational study mentioned earlier (section V.B) reported that rates of psychiatric/behavioral AEs were significantly lower with lamotrigine (4.8% of 547 patients, *P* < 0.001) than average (Weintraub et al., 2007). Kato et al. (2011) used the Buss–Perry Aggression Questionnaire and showed significant improvement from baseline in aggression overall (and the anger subscale) after 10 weeks of adjunctive lamotrigine in patients with TLE. Other observational studies and case series are detailed in Supplemental Table 1, and the results differ among the different populations and comorbidities. In general, lamotrigine has no detrimental effects with respect to aggressive behavior, although there are some reports of aggressive behaviors in patients with epilepsy with intellectual disability (Ettinger et al., 1998). In this population, the possibility of the “release phenomenon” must be considered: releasing the individual from the additional handicap of ongoing seizures can lead to difficult behavior as his or her abilities improve (Besag, 2001). Lamotrigine may also have an alerting effect that could lead individuals with intellectual disabilities to become much more reactive to their environment. In both eventualities, appropriate behavioral guidance can lead to longer-term positive outcomes (Besag, 2001).

#### 11. Levetiracetam

##### a. Randomized Controlled Trials with Behavior-Specific Endpoints

We identified only one RCT that used a behavior-specific endpoint (the anger-hostility subscale of the POMS) to explore aggression with levetiracetam (Supplemental Table 1). This prospective study by Labiner et al. (2009) showed that anger-hostility subscale scores were significantly worse with levetiracetam relative to lamotrigine, both after 20 weeks of add-on treatment and throughout the study. Scores were worse than baseline with levetiracetam at 14 of the 20 weeks, whereas scores were improved at every week with lamotrigine (significant difference versus levetiracetam at 12 of these weeks and overall). One RCT of levetiracetam versus placebo with a behavior-specific endpoint was conducted in adolescents and is reported in section VI.B.10 (de la Loge et al., 2010).

##### b. Observational Studies with Behavior-Specific Endpoints or Designed to Explore Aggression/Behavior

Six observational studies explored behavioral effects of levetiracetam using behavior-specific measures, and three additional observational studies used nonbehavior-specific endpoints (e.g., the Liverpool Adverse Event Profile) with the defined intent to explore adverse behavior or aggression (Supplemental Table 1).

In a prospective open-label study in 71 patients with epilepsy, Lee et al. (2011b) saw significant improvement from baseline in the Beck Anxiety Inventory after adding levetiracetam and no significant changes in most other measures and subscales (including a hostility subscale). However, five patients (6.5%) discontinued due to psychiatric symptoms (nervousness, irritability, anxiety, hostility, depression, and suicidal ideation or attempt) (Lee et al., 2011b).

In the large retrospective chart review already mentioned (Weintraub et al., 2007), levetiracetam had the highest incidence of psychiatric/behavioral AEs (16%), leading to discontinuations in 8% of 521 patients with epilepsy taking levetiracetam. These rates were significantly higher than average (*P* < 0.001), and rates of individual AEs (irritability, 9%; behavioral change, 3.5%) were also significantly higher than average (*P* < 0.001).

In an interview-based study, which included carers as well as patients, negative behavior change was reported in 37% of 288 levetiracetam-treated patients and positive change was reported in 22%, whereas any behavioral change was reported in only 9% of patients not taking levetiracetam. Patient reports agreed with carer reports of behavior change in most cases (85%). Aggression was the most prominent negative feature of the symptom complex and clustered with increased energy and improved concentration (Helmstaedter et al., 2008). In a gene-association study based on these patients, polymorphisms in three genes related to DA activity and signaling were associated with negative psychotropic AEs with levetiracetam, suggesting that reduced DA activity may predispose patients with epilepsy to developing psychotropic AEs with levetiracetam (Helmstaedter et al., 2013). The mechanisms underlying this association are not yet understood (see also section IV).

In a very short-term study (1 week), no differences in patient performance on several neuropsychological tests and anxiety questionnaires were seen between levetiracetam (n = 10) and pregabalin (n = 10) treatment. Some improvements from baseline were seen with levetiracetam (e.g., improved anxiety), but no aggression-specific scales were used (Ciesielski et al., 2006).

In a prospective chart review of 517 patients with various epilepsies treated with adjunctive levetiracetam, 10% developed psychiatric AEs, most commonly aggressive behavior (3.5%). Risk factors were history of febrile convulsions, previous psychiatric history, and history of status epilepticus (Mula et al., 2003b). A related study looked just at patients with epilepsy and learning difficulties prescribed levetiracetam (*N* = 118); the investigators reported that 15 patients (12.7%) developed psychiatric AEs (most commonly aggression, 7.6%), and levetiracetam was discontinued in 7.6% (Mula et al., 2004). Again, previous psychiatric history was a risk factor for psychiatric AEs (Mula et al., 2004).

In a chart review of 108 patients with epilepsy treated with both levetiracetam and topiramate (at different times); overall, 13% and 30% had psychiatric AEs with levetiracetam and topiramate, respectively (Mula et al., 2007).

In a case-control study of 553 patients taking levetiracetam, 7% of patients discontinued levetiracetam due to behavioral abnormalities (most commonly depression and irritability) and 1.8% (*n* = 10) were considered a danger to themselves or others (White et al., 2003).

In a study of patient-reported side effects with data from the UK AED register, Wieshmann and Baker (2013) found that 49% of 158 patients taking levetiracetam reported anger as always or nearly always being a problem, compared with 39% of 260 patients treated with other AEDs and 7% of the 41 controls (people with infrequent seizures and not taking AEDs) (Wieshmann and Baker, 2013). This highlights the high rate of anger when patients with epilepsy are asked the question directly, as well as the higher rate with levetiracetam than with other AEDs.

An earlier, smaller study by the same group using the same registry reported that of 100 patients with epilepsy taking AEDs, 33% of the 12 patients taking levetiracetam said anger was always a problem, compared with 19% of the 21 patients taking valproic acid, 16% of the 36 patients taking carbamazepine, and 15% of the 20 patients taking lamotrigine (Wieshmann et al., 2011).

A more recent observational study in 163 patients taking levetiracetam reported aggressive behavior as “always” a problem in 9.8% of the patients. These patients also had a 7-fold increased risk of being depressed, as measured with the Neurologic Depression Disorders Inventory–Epilepsy (Mula et al., 2015).

##### c. Randomized Controlled Trials with Aggression Data Extracted from Overall Adverse Event Reporting

A recent meta-analysis of 10 RCTs of add-on levetiracetam provides some useful information, although it does not capture all AEs across these studies (Mbizvo et al., 2014). Each individual study reported only AEs occurring in ≥5% (≥10% in some studies) of patients, so any AEs in the individual studies occurring below these thresholds were excluded from the analysis entirely. Hence, agitation was reported as an AE in one study in children (6 of 101 versus 1 of 97 with placebo) and one study in adults (3 of 28; 11% versus 0% with placebo), and any agitation AEs below the reporting thresholds in the other studies were ignored to give an overall incidence of agitation of 9 of 1092 (0.82% versus 0.14% with placebo). Similarly, one study (in adults) reported irritability as an AE (5 of 77; 6.5% versus 0% with placebo), giving an overall incidence of irritability across the meta-analysis of 0.46% (versus 0% with placebo).

Two earlier systematic reviews are more useful, because they included all AE data from the levetiracetam database of adjunctive use in focal seizures (French et al., 2001; Cramer et al., 2003). The review by French et al. (2001) also includes healthy volunteers, elderly patients with cognitive decline, and patients with anxiety. French et al. (2001) reported a rate of behavioral AEs of 13% in 769 patients treated with levetiracetam (versus 6% with placebo) in placebo-controlled epilepsy trials, 6.0% versus 4.1% in elderly patients in cognitive studies, and 5.1% versus 5.5% in anxiety studies. Across all of the levetiracetam epilepsy studies analyzed by Cramer et al. (2003) (short-term placebo-controlled trials and long-term open-label extensions), affective-type behavioral AEs were seen in 25.4% of 1393 levetiracetam patients (versus 6.2% with placebo). These included agitation (1.6% versus 0.2%), emotional lability (3.0% versus 0.2%), hostility (3.3% versus 0.9%), or nervousness (7.3% versus 1.8%) (Cramer et al., 2003). Rates of behavioral AEs were lower in levetiracetam trials in nonepilepsy indications, suggesting that patients with epilepsy are biologically more vulnerable to behavioral/psychiatric AEs (French et al., 2001; Cramer et al., 2003).

The individual levetiracetam RCTs are not discussed here, and any RCTs we found that reported an aggression-related AE are listed in Supplemental Table 1.

##### d. Observational/Open-Label Studies with Aggression Data Extracted from Side-Effect Reporting

Our searches retrieved eight such studies, which are detailed in Supplemental Table 1. One large, long-term study was comparative (*N* = 828), reporting discontinuation due to behavioral adverse effects in 19% of the 196 patients taking levetiracetam, compared with 2%–7% with oxcarbazepine, lamotrigine, topiramate, and zonisamide (Chung et al., 2007). In the other studies we retrieved (see Supplemental Table 1), rates of individual behavioral AEs with levetiracetam ranged from 5% to 24%, with the highest rate being the incidence of irritability in a retrospective chart review of 568 patients treated with levetiracetam monotherapy/polytherapy at a tertiary epilepsy center (Kang et al., 2013). These studies generally approximated normal clinical use of levetiracetam, although some excluded patients with a history of serious psychiatric disorders and some expressly included patients with a psychiatric history or mental handicap. Discontinuation due to aggression-related adverse effects was reported in 4.1%–10% of the overall populations in these studies (4.1%–5.6%, if limited to studies with approximately 100 patients or more) and discontinuations due to behavioral adverse effects in general were reported in 19% (Kang et al., 2013).

A number of case reports were retrieved, which in general we have excluded from this review. However, we selected one case series and one case study for a brief mention, with all of the caveats that apply to interpreting case reports.

Dinkelacker et al. (2003) described a case series of 33 patients with epilepsy who experienced aggressive episodes during levetiracetam adjunctive therapy; the authors estimated that 3.5% of all of their patients with epilepsy experienced irritability or aggression attributable to levetiracetam. In 24 of the 33 patients they described, irritability was transient, none of the 24 patients had a history of unprovoked impulsive aggression, and 10 patients required dose reduction or discontinuation of levetiracetam. In the remaining nine patients (one woman, eight men), the aggressive symptoms were severe (including physical violence) and necessitated emergency psychiatric treatment in two cases. In four of these nine patients, there was a history of irritability or aggression, and one patient had experienced aggressiveness with gabapentin treatment (Dinkelacker et al., 2003).

In a case study of a homicide during postictal psychosis, in which the perpetrator had a complex psychiatric and seizure history, the authors stated that AED changes may have had a contributing role. Discontinuation of carbamazepine 9 months before the homicide may have “increased the propensity for postictal mood dysregulation,” and the addition of levetiracetam at the same time “may have increased impulsiveness” (Eisenschenk et al., 2014).

#### 12. Oxcarbazepine

Two relevant studies in adults were retrieved. In the study by Weintraub et al. (2007) mentioned previously, 5.6% of 162 oxcarbazepine-treated patients had behavioral/psychiatric AEs, slightly lower than the overall average, and no irritability was seen. In a retention study by Chung et al. (2007), only 2 of 97 patients discontinued oxcarbazepine due to behavioral AEs, the lowest rate of all of the AEDs that were included.

#### 13. Perampanel

Our searches retrieved one of the three perampanel phase III RCTs in epilepsy, the open-label extension, a meta-analysis of all perampanel double-blind RCTs (epilepsy and other indications), an observational study, and a case study. By searching reference lists (excluding duplicates, data that had previously been published, or extension studies that had been superseded by more recent follow-up) and recent conferences (and excluding conference abstracts that had since been published in full), we ultimately retrieved 27 relevant articles (see Supplemental Table 1 and below).

##### a. Randomized Controlled Trials with Behavior-Specific Endpoints

We found no studies in adults with behavior-specific endpoints (see section VI.B.12 for a perampanel study in adolescents with behavior-specific assessments). Post hoc analyses specifically designed to explore aggression in perampanel RCTs are reported later in this section.

##### b. Observational Studies with Behavior-Specific Endpoints or Designed to Explore Aggression/Behavior

We found no observational studies in adults with behavior-specific endpoints.

##### c. Randomized Controlled Trials with Aggression Data Extracted from Overall Adverse Event Reporting

Of the five perampanel RCTs in epilepsy (three phase III, two phase II), two reported aggression-related AEs (French et al., 2012, 2013), whereas one phase III study and the two phase II studies did not (Krauss et al., 2012). A pooled analysis of all data from the phase III trials showed that 30 of the 225 patients taking 12 mg perampanel (11.8%) reported irritability as an AE (versus 2.9% with placebo and 3.9%–6.7% with 2–8 mg perampanel), and aggression as an AE in 3% of patients taking 12 mg perampanel (versus 1% with placebo, 1% with 4 mg, and 2% with 8 mg). No other aggression-related AEs occurred in ≥5% of patients in any treatment group (Steinhoff et al., 2013). Serious psychiatric AEs were seen in 12 patients (1.2%) taking 12 mg perampanel (versus 0.9% with placebo), with aggression being the most common (3 of 1038 patients taking perampanel or 0.29% versus none with placebo). Because of the interest in aggressive behavior with AEs, a further analysis was conducted in the pooled phase III clinical trial population, using both broad and narrow SMQs for events suggestive of hostility or aggression. The broad SMQ identified events broadly suggestive of hostility or aggression (e.g., including events like laceration, regardless of cause) in 6% of placebo-treated patients and in 5% (4 mg), 12% (8 mg), and 20% (12 mg) of perampanel-treated patients (Steinhoff et al., 2013). Homicidal ideation and/or threat were exhibited in 0.1% of 4368 perampanel-treated patients in controlled and open-label studies, including nonepilepsy studies (Steinhoff et al., 2014a). A population pharmacokinetic–pharmacodynamic study using the pooled phase III epilepsy population confirmed a significant association between perampanel plasma concentration and incidence of aggression (Gidal et al., 2013). A meta-analysis of nine RCTs including studies in Parkinson disease (Zaccara et al., 2013) reported on 3947 patients, 2627 of whom had been randomized to perampanel. In this analysis, similar terms were merged (e.g., irritability and aggression). No AEs were significantly associated with dosages of 2 or 4 mg/d, and the only behavioral AE significantly associated with any dosage of perampanel was irritability (which included aggression) with 12 mg/d perampanel.

Several post hoc analyses of the perampanel epilepsy and nonepilepsy clinical trial databases have been conducted; aggressive/irritable behavior was seen only in epilepsy populations (Ettinger et al., 2014; LoPresti et al., 2014), and the incidence was not significantly different in patients taking concomitant levetiracetam (versus no concomitant levetiracetam) (Fain et al., 2015) (see Supplemental Table 1 for details of these analyses). After our searches were completed, these conference abstracts were recently published as a full article (Ettinger et al., 2015).

The most recent analysis of the open-label extension study to the phase III trials, which followed patients for a median of 1.5 years, reported irritability in 11.5% and aggression in 5.1% of patients over the entire exposure period (*N* = 1216). Overall, 3.9% of patients had ≥1 psychiatric serious AE. Among these were 2 serious AEs of agitation (0.2%) and abnormal behavior (0.2%) and 12 serious AEs of aggression (1.0%). Aggression resolved in five patients while they continued perampanel, and seven patients discontinued (Krauss et al., 2014). Irritability and aggression led to the discontinuation of perampanel in 1.3% and 0.4% of patients, respectively.

The recently completed phase III study of perampanel in patients with primary generalized tonic–clonic seizures reported irritability as the only individual AE with an incidence of ≥5% (11.1% of 81 patients with perampanel versus 3.7% of 81 patients with placebo). The combined incidence of hostility- or aggression-related AEs (as per the broad and narrow SMQ terms) was 18.5% with perampanel versus 4.9% with placebo; using just narrow SMQ terms (i.e., AEs very likely to be related to aggression or hostility), the rates were 2.5% with perampanel and 0% with placebo (French et al., 2015).

##### d. Observational/Open-Label Studies with Aggression Data Extracted from Side-Effect Reporting

We identified two published observational studies. In one (a prospective, multicenter clinical audit of adjunctive perampanel in 281 patients with refractory epilepsy), low rates of aggression (2.8%) and irritability (2.1%) were reported (Steinhoff et al., 2014b). In the other (a single-center case series of 47 patients), the median dose taken by patients was 8 mg. The most common AEs requiring withdrawal of perampanel were behavioral: aggressive behavior in two cases (4.3%), suicidal ideation in two cases (4.3%), and aggressive behavior together with suicidal ideation in one further patient (2.1%) (Coyle et al., 2014). Another 11 observational studies (*N* = 16–111) were reported in conference abstracts, with populations ranging from institutionalized adults with highly refractory epilepsy to less severe patients in general neurology practice (see Supplemental Table 1). Aggression, irritability, and/or behavioral disturbance were reported as AEs in most (but not all) abstracts, ranging in incidence in those reports from 8% to 36% (Supplemental Table 1).

##### e. Case Studies

In a patient with moderate intellectual disability, a history of challenging behavior, and severely refractory epilepsy, the addition of perampanel 8 mg significantly improved seizure frequency and duration but worsened aggressive behavior and resulted in the patient being unable to live at home, despite reducing the perampanel dose and the addition of antipsychotics (Dolton and Choudry, 2014).

#### 14. Phenobarbital

Our searches retrieved only one relevant study with phenobarbital (Hanzel et al., 1992). The authors of this case report suggested that phenobarbital may cause behavioral AEs or exacerbate maladaptive behaviors, which may mask or suppress the effectiveness of neuroleptics (in this case, chlorpromazine) in treating aggressive behavior in patients with seizures and comorbidities (Hanzel et al., 1992). We also considered data in healthy patients. In 49 healthy adults who underwent a battery of neuropsychological tests, scores in the anger subscale of the POMS were significantly worse (increased anger) than baseline after 1 month taking 15 mg/d phenobarbital, but not with phenytoin (30–100 mg/d) or valproate (250 mg/d) (Meador et al., 1995).

#### 15. Phenytoin

Only one relevant study with phenytoin was retrieved. In this observational study in 43 newly diagnosed patients with epilepsy, a small decrease in irritability (assessed with the POMS scale) was seen after phenytoin treatment was commenced (Pulliainen and Jokelainen, 1994).

#### 16. Pregabalin

One study with pregabalin specifically investigated the neuropsychological and psychiatric effect of pregabalin and levetiracetam in 20 patients with epilepsy but only over the course of 1 week of treatment, so the results have very limited utility. No major neuropsychiatric effects were seen (Ciesielski et al., 2006). Two observational studies in refractory epilepsy, one in inpatients with intellectual disability and one in outpatients, showed infrequent behavior-related AEs (Huber et al., 2008; Valentin et al., 2009) (Supplemental Table 1).

#### 17. Retigabine

Our searches revealed no relevant retigabine studies in adults. Neither the pivotal trial publications nor a pooled analysis of these three trials reported any aggressive or behavioral AEs above the 5% (10% in one case) reporting thresholds (Porter et al., 2007, 2012; Brodie et al., 2010; French et al., 2011). Searches of epilepsy congress abstracts for retigabine revealed no further relevant data.

#### 18. Stiripentol

Only one relevant study was retrieved, which was an observational study in Japanese adults and children with Dravet syndrome (Inoue et al., 2009). Irritability was observed in three of the eight patients aged ≥13 years in the early and intermediate period but resolved, and no adults discontinued due to irritability.

#### 19. Tiagabine

We retrieved two observational studies that examined behavior with tiagabine. The largest was the observational study by Weintraub et al. (2007), which showed high rates of psychiatric/behavioral AEs (15.8% with tiagabine versus 8.4% average), and irritability (10.5%), depression (5.3%), anxiety (5.3%), and psychosis (5.3%) with tiagabine; however, the data are based on only 19 patients. A small controlled study used a mood and behavior rating scale and found no significant effect of tiagabine on these scores (Sveinbjornsdottir et al., 1994). Adverse event reporting from this study showed that 3 of the 19 patients withdrew early due to aggression; aggression/irritability was reported in 3 of 11 patients taking tiagabine in the double-blind portion of the study versus 0 of the 11 patients in the placebo period (Sveinbjornsdottir et al., 1994). The only other relevant publication was a report of two cases in which the addition of tiagabine controlled postencephalitic seizures and improved behavior (including aggressive outbursts) (Kaufman et al., 2002).

#### 20. Topiramate

We retrieved no RCTs and four observational studies that focused on behavior/aggression, as well as five observational studies from which behavior/aggression rates can be extracted from side-effect reporting (Supplemental Table 1). In their observational study, Weintraub et al. (2007) concluded that topiramate (*n* = 112) had intermediate rates (6.3% overall) of psychiatric/behavioral AEs compared with other AEDs. In a retrospective analysis, Mula et al. (2003a) reported psychiatric AEs in 24% of the 431 patients assessed, with aggressive behavior in 5.6%. In a chart review of 108 patients with epilepsy treated with both levetiracetam and topiramate (at different times), overall 13% and 30% had psychiatric AEs with levetiracetam and topiramate, respectively (Mula et al., 2007). A case series, which explored in detail 103 patients who developed psychiatric AEs with topiramate, found that nearly one-half of the sample had affective disorder, with aggressive behavior being the next most common (23%). The aggression resolved in the majority of patients with discontinuation (or reduction) of topiramate (Mula and Trimble, 2003). Aggressive behavior was associated with seizure worsening, suggesting that it was a direct expression of the epileptic state (ictal, preictal, or postictal; see earlier).

In a retention rate study by Chung et al. (2007), topiramate had the lowest retention rate (44.2%), but few of the discontinuations were due to behavioral AEs (5 of 156 patients). In the other observational studies we retrieved, irritability was reported in a wide range of patients, from <1% up to 20%, and discontinuation due to irritability/aggression/agitation was reported in 5%–12% (see Supplemental Table 1 for details) (Tartara et al., 1996; Stephen et al., 2000; Bootsma et al., 2004; Voronkova et al., 2007).

#### 21. Valproic Acid

We identified one prospective, controlled study in adults that used aggression-specific endpoints and we also identified one small observational study (Supplemental Table 1). In a prospective study using cognitive and behavioral endpoints, both treated (seizure-free) and untreated (newly diagnosed or never-treated) patients with epilepsy had worse aggression (total and verbal) compared with healthy controls, and total aggression scores were worse in patients treated with valproic acid compared with carbamazepine (Shehata et al., 2009). In a small observational study (*N* = 55) conducted in 1978, aggressive behavior was seen in one patient (Ruuskanen et al., 1979).

#### 22. Vigabatrin

Our searches identified one individual RCT with vigabatrin that reported behavior-related AEs (Loiseau et al., 1986), as well as one pooled analysis across all vigabatrin trials (randomized, double-blind, placebo-controlled studies of add-on vigabatrin) that aimed to explore psychiatric AEs (Levinson and Devinsky, 1999). The small (*n* = 23) crossover study reported irritability as an AE in two patients during the placebo phase and one patient during the vigabatrin phase (Loiseau et al., 1986), and the pooled analysis showed an odds ratio of > 1 for incidence of aggressive reaction and for agitation, but this was small and not significant (Levinson and Devinsky, 1999). We manually identified a Cochrane review of vigabatrin clinical trial data up to October 2012 (*N* = 747), and this meta-analysis did not report any behavioral events significantly associated with vigabatrin (Hemming et al., 2013). Therefore, we did not extend our manual searches any further to evaluate any individual vigabatrin clinical studies.

We identified one small observational study in adults with refractory epilepsy and severe learning difficulties. Of 22 patients treated with add-on vigabatrin, aggression and agitation were reported in 4 (18%) and 2 (9%), respectively (Armour et al., 1992).

Behavioral disturbances and aggression were reported with vigabatrin in a number of case reports soon after its approval (e.g., in 7 of 145 patients in one letter to the editor in the *Lancet*, and 9 of 119 patients in another letter) and the outbursts were occasionally extreme and violent (Robinson et al., 1990). A follow-up of 136 patients in whom behavioral problems were reported with vigabatrin to either the authors or the manufacturers found sufficient detail for 81 of these patients, depression in 22 patients, and psychosis in 28 patients. There was no mention of aggression-related behaviors in this report (Thomas et al., 1996). A study of cognitive and behavioral effects in healthy volunteers found no effects of vigabatrin on cognition or mood/behavior, although this was only with short-term use (Thomas and Trimble, 1996).

#### 23. Zonisamide

Our searches retrieved no studies that used aggression- or behavior-related endpoints, but one RCT and one meta-analysis reported aggression-related AEs (Sackellares et al., 2004; Carmichael et al., 2013), and two observational studies reported aggression-related AEs (Chung et al., 2007; Weintraub et al., 2007), one of which was specifically designed to explore psychiatric/behavioral effects of AEDs (Supplemental Table 1).

The recent meta-analysis of zonisamide studies (studies published up to February 2013) found a significant association between zonisamide and agitation/irritability (relative effect versus placebo 3.25; 95% confidence interval, 1.05–5.27). The individual study retrieved by our searches (Sackellares et al., 2004) was included in this meta-analysis, so it is not discussed separately here. The observational studies we retrieved have been discussed earlier in this review. Weintraub et al. (2007) concluded that zonisamide (*n* = 192) had an intermediate rate of psychiatric/behavioral AEs (9% versus 8.4% average), and Chung et al. (2007) reported few discontinuations due to behavioral AEs with zonisamide (9.8% of 128 patients versus 19% of 196 patients with levetiracetam).

### C. Conclusions

A previous review of the psychotropic effects of AEDs concluded that “There is a need for large-scale, prospective, randomised, double-blind, placebo-controlled studies that are properly designed to assess psychotropic effects of AEDs so as to control for confounding factors” (Piedad et al., 2012). Our review of the data, focusing just on aggression-related behaviors, must come to a similar conclusion: that few well-designed and reliable studies have been performed. Although it did not use behavior-specific assessments and does not include all currently available AEDs, the comparative, observational study by Weintraub et al. (2007) gives perhaps the most useful information. This study found levetiracetam to have the highest rates of behavioral AEs, and gabapentin to have the lowest (Weintraub et al., 2007).

Information from regulatory drug trials should be treated with caution, because the majority do not include behavior-specific endpoints and interpreting AE reporting across the spectrum of aggression-related behaviors is fraught with difficulties. More information is available on the newer AEDs because with each new approval, scrutiny of psychotropic side effects is increasing; however, equivalent scrutiny is often not possible for older AEDs because the questions were not asked of their regulatory trial data. Another gap in the literature is in regard to the relationship between AEs (e.g., aggression) and efficacy. Reports suggest that AEDs may have different side effects when the drug is effective (i.e., patients enter remission) and when treatment fails to deliver seizure freedom (Shih and Ochoa, 2009), but none of the studies we reviewed included any information on the relationship between antiseizure effects and aggressive behaviors. This relationship is further complicated by the fact that uncontrolled epilepsy may impair behavior in similar ways to the side effects of AEDs (Baker and Marson, 2001).

Based on available data, levetiracetam, perampanel, and topiramate seem to be associated with increased rates of irritability, hostility, and/or aggression, particularly in patients with a previous history of psychiatric symptoms. For such patients, these drugs should be used with caution. Product labels are generally consistent with this conclusion and provide a useful benchmark. From US labels, 13% of adults taking levetiracetam (pooled, all doses) have nonpsychotic behavioral symptoms, 12% of patients taking 8 mg perampanel have hostility/aggression-related AEs (20% with 12 mg), and 2%–10% of adults taking topiramate have aggression-related AEs (Table 3).

There are mixed data for vigabatrin, valproic acid, and zonisamide. Early vigabatrin case reports of sometimes violent aggressive behavior are not supported by evidence of increased rates in pooled analyses, although very high rates of irritability (23%) are reported in the vigabatrin US label (Sabril, 2013). One controlled study with valproic acid showed significantly worse aggression than with carbamazepine, but we found no other data supporting a risk of aggressive effects. Data from zonisamide clinical studies show an increased rate of agitation/irritability versus placebo but low rates in observational studies and no evidence for aggression in studies using behavior-specific measures.

There are reasonable data supporting no specific risk of aggression-related behavior with carbamazepine, eslicarbazepine acetate, gabapentin, lamotrigine, oxcarbazepine, and retigabine.

There are insufficient data for any conclusions for clobazam, clonazepam (although the clonazepam US label reports behavior problems in approximately 25% of patients with epilepsy; Klonopin, 2013), ethosuximide, felbamate, gabapentin, phenobarbital, phenytoin, pregabalin, and tiagabine. Because the developmental AED brivaracetam has only been used in clinical trial populations to date, there is insufficient available evidence, although the incidence of nonpsychotic behavioral AEs appears to be approximately 7% (D’Souza et al., 2012).

## VI. Aggression with Antiepileptic Drugs in Children and Teenagers with Epilepsy

### A. Introduction

The most striking aspect of the literature search in this area is, once again, the lack of high-quality data. There are almost no blinded, controlled studies using accepted measures of behavioral disturbance or providing adequate information either on the nature of the aggressive behavior itself or on factors that would allow a reasonable evaluation of causation. As stated earlier, a more comprehensive discussion of the confounding factors that could lead to false attribution of behavioral disturbance to a specific AED has been published elsewhere (Besag, 2001). The lack of data is surprising, considering the size of the problem. A questionnaire survey by Brown (1994) showed that 60% of the 896 children and teenagers surveyed felt that their antiepileptic medication caused tiredness and >50% viewed it as being responsible for poor concentration. Approximately 50% reported that that they were “cross/irritable” and about 30% reported being “angry” as a result of the medication (Brown, 1994). Although this survey was conducted in a highly selected population (namely, members of the British Epilepsy Association) and lacked a control group, the results are striking and highlight the need for attention to aggressive adverse effects of AEDs.

### B. Review of Data for Each Antiepileptic Drug

The evidence for aggression associated with each of the AEDs (in alphabetical order) is presented. In general, data in very young infants (e.g., those with infantile spasms) are not included, because AEs of irritability in these babies cannot be interpreted as aggressive behaviors; however, the data are reported (with caveats) where infants formed part of a larger population that cannot be separated.

#### 1. Acetazolamide

No evidence was found.

#### 2. Carbamazepine

No evidence was found.

#### 3. Clobazam

We identified one double-blind, randomized trial with clobazam that included behavior-specific measures (Paolicchi et al., 2015). Using data from a trial of clobazam to treat Lennox–Gastaut syndrome, Paolicchi et al. (2015) carried out a post hoc analysis of all randomized pediatric patients (aged ≤18 years) treated with at least one dose of study drug or placebo. Of the 146 clobazam-treated patients, aggression-related AEs were seen in 23 (15.8%), compared with 8.3% of placebo patients. In patients taking high- and medium-dose clobazam, most of the aggression-related AEs occurred during the 3-week titration period, whereas they were evenly distributed in the low-dose and placebo groups during the 15-week study. The aggression-related AEs included the following MedDRA-preferred terms: aggression, irritability, abnormal behavior, perseveration, and negativism (although no patients fell into the perseveration category and only one fell into the negativism category). Three patients discontinued clobazam because of aggression-related AEs. There was no significant difference between clobazam and placebo in the behavior item scores on the Achenbach Child Behavior Checklist (CBCL), although there was a trend toward worsening scores in the aggression domain with clobazam in patients with a history of aggressive behavior (Supplemental Table 2). The authors concluded that the overall rate of aggression was low with clobazam, was dose dependent, resolved by study end, and was independent of the history of aggression/behavioral problems (Paolicchi et al., 2015). The other clobazam studies we retrieved did not use behavior-specific measures but reported behavior-related AEs in varying degrees of detail (Supplemental Table 2). In a small, randomized, blinded (double dummy) study, Bawden et al. (1999) compared 24 patients taking clobazam with 17 receiving standard monotherapy (9 received carbamazepine and 8 received phenytoin). Three of the patients taking clobazam exhibited externalizing behavioral AEs, compared with three who received standard monotherapy; three taking clobazam exhibited internalizing behavioral AEs, compared with two taking standard monotherapy (Bawden et al., 1999). The rate of behavioral AEs was very similar between the two groups but it is difficult to draw any conclusions from such small numbers. Sheth et al. (1994, 1995) reported that 7 of 63 children (11%) treated with clobazam for refractory epilepsy developed severe behavioral disturbance. “Aggressive agitation” was reported in the seven children (mean age 6.4 years). The aggression was described by parents as being “animal-like” and included biting, kicking, head-banging, tantrums, and hyperactivity, all of which were said to be out of character (Sheth et al., 1994, 1995).

Jan and Shaabat (2000) reported a small, open, uncontrolled study of 31 children with refractory epilepsy, aged 2 months to 15 years, in whom clobazam was added to the existing antiepileptic medication in dosages of up to 2 mg/kg per day. Seven of the 31 children had adverse effects, including behavioral change in two (Jan and Shaabat, 2000). The clobazam had to be withdrawn in three children because of repeated vomiting or behavioral changes but it was not clear, from the report, whether it had to be withdrawn in both of the children with the behavioral change.

Klehm et al. (2014) reported a large retrospective chart review in 300 children (mean age 9.1 years) with refractory epilepsy who were prescribed clobazam. The children had a variety of seizure types (many had multiple seizure types), and the majority of patients (97%) were taking at least one other AED. The median starting dosage was 0.2 mg/kg per day and the average dosage at last follow-up was 0.73 mg/kg per day (range, 0.05–3.3 mg/kg per day). The median seizure reduction was 80%; the 50% responder rate was 67.7% and 84 patients (28%) were seizure free at the last follow-up. Twenty-three patients (7.7%) were reported as having AEs related to mood or behavior change (Klehm et al., 2014).

#### 4. Clonazepam

None of the clonazepam studies used behavior-specific measures; two observational studies and two reviews of previous studies were included (Supplemental Table 2). Mikkelsen et al. (1976) carried out a single-blind, placebo-controlled trial of clonazepam in 10 patients with simple absence seizures and 10 patients with myoclonic-atonic seizures. Most of the patients (17 of 20) were aged <18 years. Clonazepam had to be withdrawn from two of the patients because of severe irritability, dysphoria, and aggressiveness in one case and somnolence, behavioral disturbance, and lack of efficacy in the second case. Further details were sparse in this report.

Lander et al. (1979) reported that 22 of 40 patients treated with clonazepam for refractory epilepsy had “undesirable effects” attributable to the clonazepam, the commonest of which were drowsiness, loss of concentration, irritability, and aggression.

Kalachnik et al. (2002) reviewed behavioral adverse effects of benzodiazepines, including clonazepam, diazepam, and lorazepam, commenting that these are easily overlooked and under-recognized. They stated that behavioral adverse effects occurred in 13% of 446 individuals with mental retardation prescribed these AEDs for behavioral, psychiatric, or medical conditions. The rate of behavioral disturbance in the 208 individuals who had epilepsy was 15.4%.

In a review of clinical trials of clonazepam, Browne (1976) commented that behavioral disturbance occurred in a minority of patients, usually children. This represented an exacerbation of the previous disorder in some cases, but not in others. The behaviors were variously described as irritable, aggressive, excitable, irrational, antisocial, temperamental, violent, disobedient, noisy, and hard to discipline. In 10 studies, the percentage of behavioral disturbance ranged from 2% to 50% (median 17%). The behavioral disturbance sometimes resolved with a reduction in dosage but required discontinuation of the clonazepam in other cases.

#### 5. Eslicarbazepine Acetate

In a prospective, open-label study of 29 children aged between 2 and 17 years, aggressive behavior was reported in one child and one other child had “aggression aggravated” (Almeida et al., 2008).

#### 6. Ethosuximide

The only evidence retrieved for ethosuximide was two case studies. Yamamoto et al. (2001) reported that an 11-year-old boy with refractory myoclonic epilepsy and severe psychomotor delay had complete control of myoclonic seizures with ethosuximide but had “behavioural changes, more of the manic type.” This was attributed to the forced normalization phenomenon because the EEG was said to be almost normal during the episode (Yamamoto et al., 2001).

A case study by Chien (2011) described a 10-year-old boy who developed acute mania with psychotic symptoms and suicidal ideation with ethosuximide.

#### 7. Gabapentin

Two observational studies that reported aggression from AE data and two case studies were identified (Supplemental Table 2). Khurana et al. (1996) carried out a chart review of 32 children treated with gabapentin. Behavioral AEs occurred in 15 patients. Physician intervention was required in four children: one became more withdrawn and the other three became more hyperactive and more aggressive with violent outbursts and mood swings (Khurana et al., 1996). In three of the four children, the behaviors returned to baseline after the gabapentin was stopped.

Lee et al. (1996) reported on seven children who developed behavioral adverse effects in association with gabapentin. It is not clear how these children were selected from the larger pool of 55 children treated at this center (Lee et al., 1996). In some cases, the behaviors were present before treatment but were exacerbated when the child was treated with gabapentin. New behaviors included oppositional defiant disorder (58%) and conduct disorder (33%). Again, because of the small numbers, it is difficult to draw any firm conclusions.

The results of studies carried out by one of the current authors and his colleagues on 14 teenagers, using a standard instrument for monitoring behavior (the Rutter Behavioral Scales) before and during treatment with gabapentin, did not confirm these results. There were no significant behavioral changes with gabapentin: two subjects moved from the “nondisturbed” to the “disturbed” behavioral range and one moved from the disturbed to the nondisturbed range. In a further behavioral study on the same pool of patients, 6 female and 10 male subjects were matched for sex as well as for age and other parameters (as closely as possible). Examination of the changes in the Rutter scale scores revealed no significant differences between the gabapentin-treated group and the comparison group (Besag, 1996).

A case study by Tallian et al. (1996) reported two children who had aggressive behavior with gabapentin. The first case was a 16-year-old boy in whom the seizures were fully controlled but his appetite decreased and he had problems sleeping. He then developed aggressive behavior with biting, slapping, scratching, growling, and “acting like an animal.” The gabapentin was discontinued and the behavioral disturbance resolved. When the gabapentin was recommenced and the dose increased, the aggression became marked. When the gabapentin was subsequently discontinued, the problem resolved again. The second patient was a 6-year-old girl with intellectual disability and ADHD but no history of aggressive behavior. When gabapentin was commenced, she stopped interacting socially; she became physically aggressive when confronted socially and she assaulted other children. A moderate improvement in behavior occurred when the gabapentin dose was decreased; it was continued because her parents judged her behavior as being tolerable and she remained seizure free.

A case series described by Wolf et al. (1995) reported behavioral problems in three children with learning disability when treated with gabapentin. The behaviors described included unprovoked outbursts of anger in case one, episodes of hyperactivity and oppositional behavior in case two, and outbursts characterized by throwing food, screaming, and fighting in case three.

It is difficult to assess the causative role of the gabapentin itself in all of these reports, not only because of the small numbers and open nature of the studies but also because of confounding factors such as drug interactions and changes in seizure control.

#### 8. Lacosamide

Our searches for lacosamide data in children retrieved only small observational studies (Supplemental Table 2).

Gavatha et al. (2011) reported a study in 18 children (10 male and 8 female, aged 3–18 years) with intellectual disability. No aggression was specifically reported but there were two cases of irritability. No further details were provided (Gavatha et al., 2011).

Guilhoto et al. (2011) analyzed results from a retrospective study of 16 young people with treatment-resistant epilepsy (7 male and 9 female, aged 8–21 years, mean age 14.9 years). Lacosamide was withdrawn in one boy (aged 8.4 years) because of severe behavioral outbursts (Guilhoto et al., 2011).

Heyman et al. (2012) carried out a retrospective study of medical records of 17 children (10 male and 7 female, aged 1.5–16 years, mean age 8 years) with epilepsy taking lacosamide. Restlessness was reported in two patients but no other behavioral disturbance was reported (Heyman et al., 2012).

Kim et al. (2014) described a retrospective study of medical records that included 21 children (16 male and 5 female, aged 1.2–17.9 years, median age 13.9), 2 of whom discontinued lacosamide because of AEs (aggressive behavior and depression).

#### 9. Lamotrigine

Two observational studies were retrieved (Supplemental Table 2). In a long-term, open-label extension study, Piña-Garza et al. (2008) followed 204 infants (aged 1–24 months), treated with lamotrigine after phase III studies. The only AE that they considered could be reasonably attributable to lamotrigine in >2% of patients was irritability, which occurred in 10 patients (5%) (Piña-Garza et al., 2008).

Cardenas et al. (2010) carried out a retrospective review of patients who developed “neurobehavioural adverse reactions to lamotrigine.” They identified nine children (seven male and two female, mean age 5 years) who became hyperactive and agitated, over a wide range of doses from 0.7 to 14 mg/kg per day. Five patients developed self-injurious and violent behaviors, two had severe insomnia, and the most affected patient (a 6-year-old boy) developed “extremely volatile mood and affect” with visual and auditory hallucinations together with insomnia. All nine patients improved markedly after discontinuation or dose reduction of the lamotrigine. These authors said that severe, reversible neurobehavioral disturbance associated with lamotrigine therapy had not previously been reported in the literature.

It should be noted that lamotrigine is now widely acknowledged as an AED that can improve mood significantly; large studies have established this in adults and the positive psychotropic effects of improving mood/behavior in young people with or without epilepsy have also been confirmed (Frye et al., 2000; Cramer et al., 2004; Biederman et al., 2010).

#### 10. Levetiracetam

There are several reports of aggression in children with epilepsy treated with levetiracetam, including two RCTs with behavior-specific measures; however, there are also several reports of improved behavior (Supplemental Table 2).

Our searches identified one study that specifically explored the behavioral effects of levetiracetam in a randomized, placebo-controlled study, using standardized measures including the CBCL and the Child Health Questionnaire–Parent Form 50 (de la Loge et al., 2010). Patients received adjunctive levetiracetam (*N* = 64) or placebo (*N* = 34) for 12 weeks. The CBCL separates scores into a total problems score and a total competence score. Among the per-protocol population (levetiracetam, *N* = 46; placebo, *N* = 27), there was no difference between treatment groups in the total competence score but a significant difference in the total problems score (*P* = 0.020) between levetiracetam (worsening) and placebo (improvement). In the problems component of the CBCL, there was a significant worsening of aggression with levetiracetam versus placebo (*P* = 0.013), which drove the overall difference in the problems score. In the competence component of the CBCL, there was a small improvement with levetiracetam versus placebo in the activities subscale (*P* = 0.049). The Child Health Questionnaire–Parent Form 50 score showed little change during treatment and no significant between-group differences. A long-term extension that included 80 patients who continued from this study and 23 additional patients found no significant change from the baseline CBCL score (or aggression subscale scores) with levetiracetam (Schiemann-Delgado et al., 2012). From AE reporting in this study, aggression was seen in 7.8% of patients, irritability in 7.8%, and abnormal behavior in 3.9%.

One other study with levetiracetam specifically studied aggressive behavior but did not use standardized measures. In 12 children with epilepsy with continuous spikes and waves during slow sleep and pervasive developmental disorder, parents reported the frequency of seizures and frequency of episodes of panic or aggressive behavior. In the eight patients whose seizures improved with the addition of levetiracetam to existing AEDs, six patients also had a ≥50% reduction in the frequency of episodes of panic or aggression; there was no change in episode frequency in the other two responders (Kanemura et al., 2014).

There have been many RCTs and observational studies with levetiracetam in children and adolescents; rather than review them all here, the results of a recent systematic review and meta-analysis of the behavioral effects of levetiracetam are presented (Halma et al., 2014). These authors included studies in children aged from 1 month to 18 years with a diagnosis of epilepsy, who were taking oral levetiracetam as monotherapy or add-on therapy, with follow-up of at least 2 weeks and which reported behavioral side effects. They excluded case studies or case series with fewer than 10 patients, studies of neonatal convulsions, and studies that included adults and did not have separate subgroup analyses for patients aged <18 years. Their review identified 13 studies in 727 patients. In the three RCTs they identified, the most frequent behavioral AEs with add-on levetiracetam (*N* = 165) were hostility (7.3%), nervousness (6.1%), and aggression (4.9%). A meta-analysis of these studies revealed a statistically significant increased risk of behavioral AEs with levetiracetam (risk ratio 2.18 versus placebo; 95% confidence interval, 1.42 to 3.37). Ten observational studies met their selection criteria and these reported both worsening and improvement of behavior with levetiracetam. Levetiracetam add-on therapy was associated with behavioral AEs of irritability (4.7%), hyperexcitability (4.4%), and aggression (2.7%); monotherapy was associated with behavioral problems in general (19%) and irritability (2.6%). No meta-analysis was possible across the observational studies.

Our searches identified the majority of the studies included by Halma et al. (2014) in their systematic review and meta-analysis, as well as a number of additional studies that were excluded by their selection criteria. Details of all of these studies can be found in Supplemental Table 2; the rates of behavioral AEs they report are broadly in line with those reported by Halma et al. (2014).

Mbizvo et al. (2014) performed another meta-analysis of levetiracetam studies, which was also discussed earlier (section V.B.11). This meta-analysis only included two RCTs in children/adolescents, whereas Halma et al. (2014) included three; when all of the captured behavioral AE terms were combined across these two studies for the child/adolescent populations, an incidence of 40.6% (versus 21.4% with placebo) was found. This seems remarkably high, especially considering the fact that the analysis excluded behavioral AEs that were not reported in the original publications because they were below the reporting thresholds (≥5% or ≥10%); however, it is broadly consistent with the doubling of risk (i.e., a risk ratio of approximately 2 versus placebo) as reported by Halma et al. (2014). The terms included by Mbizvo et al. (2014) were hostility, personality disorder, nervousness, depression, aggression, agitation, emotional lability, psychomotor hyperactivity, irritability, abnormal behavior, altered mood, anxiety, and dissociation. It was not clear, from their article, whether they excluded “double counting” (e.g., adding together reports of AEs of aggression and agitation when these occurred in the same subject); if they did not exclude double counting, this could explain the very large percentages of behavioral AEs in both the levetiracetam and placebo groups.

#### 11. Oxcarbazepine

Very little evidence of any aggression-related features was found for oxcarbazepine, and just one open-label study was identified (Supplemental Table 2). Northam et al. (2005) carried out a prospective, open-label study of 24 young patients (aged 2–45 months). Oxcarbazepine was associated with irritability in 5 of 24 patients (21%) in the treatment phase (up to 30 days) and in 7 of 20 (35%) in the 6-month extension phase (Northam et al., 2005). Oxcarbazepine was discontinued in one of the patients with irritability (who also had fatigue and ataxia). The usual comments about the reliability of findings from small, open, uncontrolled studies apply.

#### 12. Perampanel

Our searches identified one study with perampanel in adolescents that used standardized measures to assess behavior, one pooled analysis of phase III clinical trials, and several observational studies (Supplemental Table 2). Lagae et al. (2014) reported a phase II, randomized, placebo-controlled trial of add-on perampanel in 133 adolescents with refractory focal-onset epilepsy; behavior was assessed with the CBCL. There was no significant difference between perampanel and placebo in the change from baseline in CBCL total problem or total competence scores, or in any of the subscales (e.g., aggression). AEs related to hostility or aggression occurred in 17.6% (*n* = 15) of the perampanel-treated subjects (aggression, *n* = 7; irritability, *n* = 6; anger, *n* =2; and laceration, *n* = 1) and 4.2% (*n* = 2) of the placebo group (aggression, *n* = 1; irritability, *n* = 1).

Rosenfeld et al. (2015) reported pooled AE data for the 143 adolescents in the three phase III trials of perampanel. The most common AEs included aggression in 8.2% of adolescents versus 0% for placebo; this was more frequent than in the overall perampanel-treated population (1.6%). Furthermore, aggression was reported as being one of the most common reasons (6.6%) for interruption or dose adjustment of the perampanel among adolescents during the extension phase (Rosenfeld et al., 2015).

The other perampanel reports come from observational studies. Biró et al. (2015) published a retrospective analysis of 58 children (mean age 10.3 years; range, 2–17 years) treated with perampanel. Aggression was reported in eight patients (13.8%). In a retrospective study of 18 patients (age range, 4–19 years), Philip et al. (2014) reported behavioral change in only 1 patient (5.6%).

#### 13. Phenobarbital

Four studies were identified for phenobarbital (Supplemental Table 2). Willis et al. (1997) carried out a neuropsychological and EEG study of 11 children with epilepsy aged 7–14 years treated with phenobarbital and mephobarbital. They stated that parents reported clear behavioral changes in 6 of 11 subjects, including irritability, oppositional attitude, and overactivity. In four of the six patients, the changes were relatively mild and the barbiturate was not discontinued.

In a large observational study that used parent questionnaires, Domizio et al. (1993) compared 197 children (116 male and 81 female, mean age 5.3 years) treated with phenobarbital, with 103 children (66 male and 37 female, mean age 6.4 years) who were treated with other AEDs. In the phenobarbital group, 150 children (76.1%) had one or more behavioral disturbances, compared with 32 (31%) in the other group (*P* < 0.0001). Hyperactivity was the most frequent behavioral disorder (Domizio et al., 1993).

In a randomized study of four AEDs (phenobarbital, phenytoin, carbamazepine, and sodium valproate), de Silva et al. (1996) discontinued the phenobarbital arm after 6 of the first 10 children taking this drug had unacceptable AEs, which were primarily behavioral. In contrast, Pal et al. (1998) found no behavioral effects of phenobarbital (*N* = 47) in a study in 94 children in India.

#### 14. Phenytoin

Although anecdotally phenytoin is associated with behavioral disturbance in young people, no firm published evidence for this was found (Supplemental Table 2). In the study in India referred to above, Pal et al. (1998) also found no behavioral effects of phenytoin in 47 children. Krishnamoorthy et al. (1983) reported three cases of phenytoin-induced choreoathetosis associated with agitation/restlessness in three young children: two were aged 2 years and one was aged 15 months.

#### 15. Pregabalin

No evidence was found.

#### 16. Primidone

Despite the fact that primidone is partly metabolized to phenobarbital, no evidence of aggression in children and teenagers with primidone was found.

#### 17. Retigabine

We identified only one study. Groening et al. (2012) treated 17 patients (aged from 1 year and 10 months to 19 years) with retigabine for pharmacoresistant seizures (Supplemental Table 2). Results were analyzed for 12 patients, and AEs included hallucinations, agitation, and personality changes. Further details were not provided in this conference abstract (Groening et al., 2012).

#### 18. Rufinamide

In a recent consensus paper on rufinamide in childhood epilepsy by Coppola et al. (2014), aggression and related behaviors were not reported among the adverse effects. There have been few other reports of behavioral adverse effects with rufinamide (Supplemental Table 2).

In a multicenter, prospective, add-on, observational study of rufinamide in 43 children, adolescents, and adults with Lennox–Gastaut syndrome (26 male and 17 female, aged 4–34 years, mean 15.9 years), Coppola et al. (2010) reported irritability/aggressiveness in 3 patients (6.9%). In a separate observational study, the effects of rufinamide in encephalopathies other than Lennox–Gastaut syndrome were reported in 38 patients (19 male and 19 female, aged 4–34 years, mean age 13.7 years). Irritability/aggressiveness was seen in two patients (5.3%) (Coppola et al., 2011).

In a prospective, open-label, add-on trial in refractory epilepsy in 69 children and adolescents, Cusmai et al. (2014) reported irritability in 11 patients (15.9%). In a retrospective study in 23 patients in Korea (age range, 4–22 years), Lee et al. (2013) reported aggressive behavior in 2 patients (8.7%).

#### 19. Stiripentol

One observational study was identified (Supplemental Table 2). Inoue et al. (2009) reported clinical results using stiripentol to treat Dravet syndrome in patients aged 1–22 years. Of 23 patients, 6 had hyperactivity/irritability early in treatment, which resolved with continued treatment or dose reduction. One patient (a 15-year-old girl) discontinued stiripentol because of early irritability (Inoue et al., 2009).

#### 20. Tiagabine

No evidence was found.

#### 21. Topiramate

Two large retrospective studies, together with several smaller studies and case studies, were identified (Supplemental Table 2). Reith et al. (2003) reported data in 159 patients with epilepsy aged <18 years who were taking topiramate. Follow-up was possible in 127 patients (0.5–17.9 years); aggression/psychosis was a treatment-limiting AE in 10 of these patients (7.9%) (Reith et al., 2003). Grosso et al. (2005) treated 59 children aged ≤2 years with topiramate for localization-related and generalized epilepsies. Irritability was listed as one of the most frequent AEs, but precise rates were not given (Grosso et al., 2005). During topiramate treatment, Lee et al. (2011a) reported that 4 of 28 infants (14.3%) aged 2–18 months with West syndrome developed irritability, and Endoh et al. (2012) reported that 5 of 33 children (15.2%) aged ≤12 years with epileptic spasms developed irritability.

Metabolic acidosis is a known adverse effect of topiramate, and this can be associated with irritability/aggression (Ko and Kong, 2001). In patients taking topiramate who present with hyperpnoea or mental status change, metabolic acidosis must be excluded as a possible cause.

#### 22. Valproic Acid

Two studies were identified with sodium valproate in adolescent epilepsy populations (Supplemental Table 2). In a retrospective study of 100 children with epilepsy treated with sodium valproate, Egger and Brett (1981) reported aggressive behavior in 4 (4%).

Ronen et al. (2000) studied eight children (six male and two female, aged 6–12 years) without clinical seizures but with abnormal EEGs and significant developmental learning disorder. While taking sodium valproate, the children were more distractible, had increased delay in response time, and had lower memory scores. Their parents also reported higher internalizing scores on the CBCL while the children were taking valproate (Ronen et al., 2000). In contrast, sodium valproate and divalproex sodium are used extensively as mood-leveling drugs in both adults and children in nonepilepsy populations; for example, Hollander et al. (2000) reported that impulsivity and aggression were decreased in 14 patients with autism taking divalproex sodium as a psychotropic medication.

#### 23. Vigabatrin

We found no RCTs of vigabatrin with behavior-specific endpoints, and we found one observational study in children and adolescents that focused on behavior (Supplemental Table 2). Sheth et al. (1996) reported that in 31 patients aged 1–22 years (mean age 12.6 years) with refractory focal and generalized seizures, add-on vigabatrin was associated with negative behavior change in 6 children (based on parent reports), with discontinuation in 1 patient with severe aggressive agitation and positive behavior change in 1 patient.

The remaining studies we identified relied on AE reporting from five observational studies, three case reports, and six studies in babies with infantile spasms (Supplemental Table 2). Gobbi et al. (1999) carried out a prospective study of vigabatrin monotherapy in the treatment of focal epilepsies in 40 children (mean age at last visit 7.5 years) compared with 40 children treated with carbamazepine monotherapy. They stated that tolerability was good in the vigabatrin group but 4 of 37 patients had mild irritability at the end of the trial (versus none in the carbamazepine-treated group). Raucci et al. (1994) studied 61 children with various types of epilepsy treated with vigabatrin, 12 as monotherapy and 49 as add-on therapy. Vigabatrin was discontinued in six children because of adverse effects, including irritability.

In the first of three case studies, Weber et al. (2012) reported psychosis associated with vigabatrin in an adolescent girl with refractory symptomatic epilepsy after an early middle cerebral artery insult. The onset of the psychosis was 7 weeks after the vigabatrin was commenced, when she had been seizure free for 2 weeks. They attributed this to the phenomenon of forced normalization, although it should, more accurately, be termed alternative psychosis or reciprocal psychosis (the situation when the psychosis is likely to occur when the seizure control has improved; see earlier). Two of the teenage patients of one of the current authors also developed psychosis with vigabatrin, which resolved with dose reduction or discontinuation (F. Besag, personal communication). Cánovas Martínez et al. (1995) published a case of a 7-year-old boy with refractory epilepsy who developed an acute psychosis 3 days after rapid introduction of vigabatrin. The psychosis resolved within 48 hours of discontinuing the vigabatrin. Recommencement of the vigabatrin 2 months later, using a slower dose escalation, was well tolerated with no return of the psychosis. Chiaretti et al. (1994) also published a single case report of psychosis with vigabatrin in a child.

The data in infantile spasms are not presented here, because AEs such as irritability in young infants cannot be interpreted as aggression-related behaviors, but studies are listed in Supplemental Table 2.

#### 24. Zonisamide

We identified one study with behavior-specific outcomes and five additional studies reporting behavioral AEs (Supplemental Table 2).

Eun et al. (2011) studied the neuropsychological and behavioral effects of low-dose and high-dose zonisamide, using a Korean version of the CBCL, in children aged 2–16 years receiving zonisamide monotherapy for newly diagnosed epilepsy. Data were available for 63 patients (27 and 36 receiving low-dose and high-dose treatment, respectively) and were presented as group data, so it is not possible to determine whether some patients became more aggressive and others less aggressive. Overall, Eun et al. (2011) saw a significant improvement (*P* < 0.05) in various parameters, including aggressive behavior, in the low-dose group and a nonsignificant improvement in the high-dose group. No aggression-related or behavior-related AEs were reported.

Cross et al. (2014) carried out a pooled analysis of 17 zonisamide studies in patients aged 16 years or younger, including 4 randomized, double-blind trials. Irritability was reported in 5.8% of the 391 zonisamide-treated patients but was not among the AEs commonly leading to discontinuation. Irritability was somewhat more common (7.5%) in patients aged 6–11 years than in patients aged 12–16 years (<5%).

A phase III study by Guerrini et al. (2013) was included in the meta-analysis by Cross et al. and is thus not discussed in detail here. No aggression-related AEs were reported in the zonisamide-treated group, but one patient in the placebo group discontinued due to aggression (Guerrini et al., 2013). In the subsequent long-term extension study, there were no behavioral AEs in patients continuing on zonisamide from the phase III study but there were two cases of aggression (2.8%) in patients who switched from placebo to zonisamide during the extension (Guerrini et al., 2014).

Coppola et al. (2009) carried out a prospective, add-on, open-label study of 82 young patients (45 male and 37 female, aged 3–34 years, mean age 13.1 years). Irritability was reported in nine patients (11.0%) but resolved in most cases with dose reduction. No other behavioral adverse effects were reported (Coppola et al., 2009).

Hirai et al. (2002) treated 27 children who had idiopathic epilepsy with zonisamide in a prospective study, observing behavioral disturbance in 2 children (7.4%). One of the cases developed an obsessive-compulsive disorder. The other case, a 14-year-old girl with focal seizures whose seizures were treated effectively with zonisamide from age 6 years, developed selective mutism, violent behavior, and lack of concentration at age 10 years. Decreasing the zonisamide dose was said to have maintained adequate seizure control while resolving the behavioral disturbance. Because of the long time interval between the prescription of zonisamide and the development of the violent behavior, the direct role of the AED is questionable in this case; a more likely explanation might be that the violent behavior was an interaction between seizure control and developmental factors.

### C. Conclusions

In most cases, there is inadequate evidence to draw any firm conclusions about the aggression-related behavioral AEs associated with AEDs in children and teenagers. From the evidence that is available, however, it is suggested that children and adolescents who are treated with gabapentin, levetiracetam, perampanel (especially at higher doses), phenobarbital, sodium valproate, topiramate, and zonisamide should be monitored closely for possible behavioral AEs. The most extensive evidence is for levetiracetam but the reports of both improvement and deterioration with this drug emphasize the need for monitoring each individual patient for positive or adverse effects of AEDs. Although there is credible evidence for psychosis developing with vigabatrin, this is more likely to be the phenomenon of alternative or reciprocal psychosis that might have occurred with any AED that achieved rapid seizure control and could probably have been avoided by starting at low doses and escalating the dose slowly. This emphasizes the importance of excluding the confounding factors that have been described in detail elsewhere (Besag, 2001) before attributing behavioral or aggression-related AEs to an AED.

## VII. Avoidance and Management of Antiepileptic Drug–Induced Aggression

Although there is a limited amount of good-quality clinical data on aggression with AEDs in children, adolescents, and adults with epilepsy, it is becoming clear that there is increased propensity for some patients to develop aggression-related behavior during treatment with some AEDs. In a recent survey, for example, 49% of 158 people with epilepsy treated with levetiracetam reported aggression as sometimes or always being a problem, whereas 39% of 260 patients taking other AEDs were aware of anger issues and only 9% of 41 controls admitted to ever losing their temper (Wieshmann and Baker, 2013). Based on the evidence collected in this review (in sections V and VI), we can conclude that 1) there is reasonable evidence of an increased risk of aggressive behaviors occurring in patients with epilepsy treated with levetiracetam, perampanel, and topiramate and 2) that use of gabapentin, phenobarbital, sodium valproate, and zonisamide in children/adolescents also carries some risk. What can we do to anticipate, identify, and ameliorate aggressive-type behaviors that can occur with these AEDs?

When it comes to anticipating which patients may develop aggressive behaviors with AEDs, the evidence is very limited. Previous psychiatric history is a predictive factor in some studies, but certainly not all patients with a history of psychiatric illness or aggressive behavior will go on to develop aggressive behavior with higher-risk AEDs. However, when initiating treatment with any AED, a personal and, if possible, a family history of psychiatric disorders should be explored and documented. All patients should be asked whether they have a short temper, how often they lose it, and what is the likely outcome. A similar question to accompanying partners and family members often elicits a different response, since some people are not aware that they have this problem or appreciate its extent. Any history of physical violence is of particular concern in this context. If the patient has had serious anger management issues in the past or has regularly demonstrated hostile or aggressive behavior, it does not necessarily completely preclude using AEDs that have evidence for increased risk of aggressive behaviors, but such AEDs should be used with extreme caution or avoided altogether based on clinical judgment. The decision will involve weighing the risks of aggressive behaviors and their possible effects against the risks of inadequate seizure control and will be influenced by the other treatment options available to the patient. Alcohol and other stimulating agents can exacerbate aggression and their effects should also be discussed with the patient, partner, and family (Heinz et al., 2011). Providing patients and/or carers with a medical contact number to call if inappropriate behavior occurs is a sensible precaution, particularly in patients who admit to frequently losing their temper or to exhibiting aggressive behavior. Regardless of the individual patient history, the potential for aggressive behavior should be explained to the patient and/or caregivers starting an AED associated with risk to aid early detection of any problems. Finally, documentation of this discussion in the case notes and in the correspondence with the patient’s general practitioner should be considered as an integral part of good practice and as an essential precaution. The possibility of later medicolegal repercussions if a serious assault subsequently occurs underlines the importance of not only following good practice but also documenting it.

In general, slow titration should be used when possible, particularly when the patient is considered at risk of aggressive behavior based on his or her psychiatric history or selection of AED.

Certain groups of patients require special attention. When introducing AEDs in teenagers, particularly those with juvenile myoclonic epilepsy, clinicians must be aware that some patients will have a tendency to exhibit impulsive behavior (Crespel et al., 2013). Particular attention should also be paid to patients with intellectual disability, who cannot easily express their frustrations in an acceptable way, and those with dementia, who may demonstrate unexpected violent behavior (Newman, 2012). The possibility of release phenomena in patients with intellectual disability should also be considered (Besag, 2001). Some AEDs, such as the sodium channel blockers carbamazepine, oxcarbazepine, and, particularly, lamotrigine (Labiner et al., 2009) or valproic acid, may be better choices in these situations (Comai et al., 2012b).

Another possibility to help anticipate aggressive responses is to use questionnaires to screen patients. There are a number of published and validated scoring systems for measuring aggression that could be used in patients with epilepsy (Silver and Yudofsky, 1991; Buss and Perry, 1992; Harris, 1995). An instrument for measuring irritability in people with epilepsy was also recently published (Piazzini et al., 2011).

If irritability, anger, hostility, or aggressive behaviors do develop or worsen, managing these depends on their severity and the extent of the positive pharmacological response to the implicated AED. This consideration is particularly important in patients with severe pharmacoresistant epilepsy in whom other treatment options may be limited. Dose reduction of the most recently added AED should be considered. Reducing alcohol intake can also be a helpful step in ameliorating aggression. Often, however, the aggressive behavior can only be stopped by discontinuing the AED. The decision whether to maintain AED treatment in this setting should be made in discussion with the patient, partner, and family; again, this discussion should be documented in the patient’s case notes. If the AED is continued, improvement in anger-related symptoms can occur with time, although this appears to be relatively uncommon, and anger management programs can also be a helpful addition to the therapeutic regimen, particularly in seizure-free patients. It is important to make the patient’s general practitioner fully aware of the situation, stating which AED is implicated.

If the AED is continued, and/or the aggressive behavior continues, then pharmacological management of the behavior may be warranted. There is, however, no single pharmacological strategy recommended for the management of anger and aggression (Newman, 2012). Antipsychotic, antidepressant, and other psychotropic agents have all been used to ameliorate these behaviors (Alper et al., 2002; Nevels et al., 2010), and there is also evidence that mood stabilizers (e.g., carbamazepine, oxcarbazepine, phenytoin, and lithium) are significantly better than placebo in reducing aggressive behavior (Jones et al., 2011). Pharmacological management of aggression can be complicated by proseizure effects of some psychotropic medications, particularly in higher doses (Varma et al., 2011). Existing treatment of psychiatric comorbidities, particularly depression, anxiety, psychosis, panic attacks, bipolar symptoms, and attention deficit disorder, should be reviewed.

If the comorbid psychiatric symptoms are chronic and severe, it is advisable that the patient also remains under the care of an experienced psychiatrist. Managing psychiatric comorbidities is not always an attractive option for the neurologist, but this should be attempted if prompt management from a psychiatrist is not available and the symptoms are relatively mild and amenable to standard pharmacological intervention with widely used mood-stabilizing drugs.

## VIII. Overall Summary

One of the major areas of interest in the management of epilepsy is the effect of psychiatric comorbidities on the choice of and response to AED therapy (Hitiris et al., 2007). There is, of course, substantial overlap among their clinical presentations in the setting of newly diagnosed and refractory epilepsy (Lin et al., 2012). There has been particular concern recently regarding the potential for some of these drugs to cause or worsen hostility and aggression, with possible medicolegal consequences. This evidence-based review discusses for the first time the relationship between epilepsy, AEDs, and aggression, covering a wide range of issues including definitions, psychiatric comorbidities and epilepsy, the neurobiology and pharmacology of aggression, and evidence for each AED in causing or exacerbating this problem in children, adolescents, and adults, and some suggestions for prevention and management are also provided. The main conclusion must be that better quality evidence and comparative studies are needed to clarify the link between AEDs and aggressive behavior in patients with epilepsy. However, based on the available evidence, some AEDs seem to be associated with higher risk than others, including clobazam, clonazepam, levetiracetam, perampanel, phenobarbital, tiagabine, topiramate, vigabatrin, and zonisamide (in alphabetical order). The potential for aggressive behavior should be explained to every patient starting treatment with any of these drugs, particularly patients with known anger management issues. The AEDs with strongest evidence for a risk of aggressive behaviors are levetiracetam, perampanel, and possibly topiramate, but the majority of patients taking these, and any other AEDs, will have no problems with aggressive behaviors. Involvement of partners and families is important, since many people are not aware that they have a short temper or that their demeanor could be perceived as aggressive. These issues should be taken into consideration when making the choice of AED therapy for all patients with newly diagnosed and chronic epilepsy. Future research should clarify the neurobiology of aggression and epilepsy and may help clinical decision making and treatment selection to avoid problems with aggression in patients with epilepsy.

## Supplementary Material

Data Supplement

## Abbreviations

5-HT: serotonin
ADHD: attention deficit hyperactivity disorder
AE: adverse event
AED: antiepileptic drug
CBCL: Achenbach Child Behavior Checklist
DA: dopamine
DSM: *Diagnostic and Statistical Manual of Mental Disorders*
EEG: electroencephalogram
ERK: extracellular signal–regulated protein kinase
MAO: monoamine oxidase
MAPK: mitogen-activated protein kinase
MedDRA: *Medical Dictionary for Regulatory Activities*
NA: noradrenaline
PCP: phencyclidine
POMS: Profile of Mood States
RCT: randomized controlled trial
SMQ: standardized MedDRA query
TLE: temporal lobe epilepsy
VGLUT: vesicular glutamate transporter
