# Supplementary material for: Epilepsy, Antiepileptic Drugs, and Aggression: An Evidence-Based Review
Source: Pharmacol Rev. 2016 Jul;68(3):563–602. doi: 10.1124/pr.115.012021 (PMC4931873; doi:10.1124/pr.115.012021)
Supplement: Data Supplement [file supp_68_3_563__index.html]

Data Supplement 

# Epilepsy, Antiepileptic Drugs, and Aggression: An Evidence-Based Review

## Data Supplement

**Files in this Data Supplement:**

- Data Supplement
