# Supplementary material for: Epilepsy, Antiepileptic Drugs, and Aggression: An Evidence-Based Review
Source: Pharmacol Rev. 2016 Jul;68(3):563–602. doi: 10.1124/pr.115.012021 (PMC4931873; doi:10.1124/pr.115.012021)
Supplement: Data Supplement [file supp_68.3.563_12021SupplementalData.pdf]

Supplemental Information

A. Search strategy

For 24 approved antiepileptic drugs (AEDs), two searches were performed in PubMed, limited to the English language, and with an end date of 13 Jan 2015 (brivaracetam, carbamazepine, clobazam, clonazepam, eslicarbazepine acetate, ethosuximide, felbamate, gabapentin, lacosamide, lamotrigine, levetiracetam, oxcarbazepine, perampanel, phenobarbital, phenytoin, pregabalin, retigabine [ezogabine], rufinamide, stiripentol, tiagabine, topiramate, valproate/valproic acid, vigabatrin, zonisamide). Duplicates were removed, and abstracts of the retrieved article were reviewed for relevance. Publications focusing exclusively on non-epilepsy indications or populations were excluded, as were animal studies. Additional searches were conducted as necessary (see search #3 below); product SPCs and USPIs were reviewed (search #4); and, for recently approved AEDs and products in development, abstracts from epilepsy congresses were also searched for data that had not yet been published in full (search #5). One researcher (K Carpenter) performed all searches, stated the exclusions, and collected abstracts. Three authors (A Aldenkamp, F Besag, and B Steinhoff [for perampanel only]) reviewed the retrieved abstracts, obtained full text, and included relevant data in the manuscript text and Supplemental tables S-1 and S-2. Researcher (K Carpenter) then checked the text and tables for accuracy against the references.

Search #1

“<<AED generic name>> AND (anger OR aggression OR irritability OR hostility OR impulsivity OR homicide OR homicidality OR agitation OR assault)”

Search #2

This search was used to identify reports using known behaviour/aggression scales. “<<AED generic name>> AND (UPPS OR CBCS OR IPAS OR DAR-5 OR SIS OR CICS OR MOAS OR OAS OR P-GBI OR BIS OR IPAS OR CBCL)

Search #3

This search was conducted to identify additional relevant articles that were not captured in the searches above. For articles retrieved in searches #1 #2 which were deemed relevant, reference lists were hand searched for additional relevant articles. In addition, for AEDs where no relevant articles were retrieved through searches #1 or #2, Phase III clinical trial

results and meta-analyses were identified and reviewed for any evidence of aggression or related behaviours.

Search #4

The most recent European SPC and US PI were retrieved for each AED, and reviewed for data, information, and warnings about aggression and related terms.

Search #5

For newer and developmental AEDs only (brivaracetam, retigabine, perampanel, eslicarbazepine) which had limited published information on clinical trials and/or clinical experience, we also manually searched available abstracts at key epilepsy meetings, and retrieved all abstracts that mentioned aggression or any of the related terms (in Search #1 above). Abstracts were excluded if the data had been superseded by a full publication. Congresses searched: AES 2000 to 2014; IEC 2013, 2011, 2009, 2007, 2005; ECE 2014, 2012, 2010.

Additional comments and exclusions

Reviews were not used as this could lead to repetitive errors of evaluation.

Anecdotal information also was omitted from evaluation.

Some relevant study publications were identified after the end of the initial search window, and these were included where relevant (e.g. Paolicchi *et al*. 2015)

For the section on AEDs and adults, we only used studies including adults only (aged ≥18), unless otherwise noted. For the section on children/adolescents, we sometimes used studies with populations that included a small number of adults (always noted), as evidence in children/adolescents was often scarce.

B. Supplemental Table S-1: Summary of the evaluated studies for each AED (in adults)

**AED**	**Reference**	**Study type/design**	**Treatment and seizure type**	**Total N**	**Behaviour-specific measures used?**	**Prevalence of aggression-related behaviour**
Studies shown in italics have been presented at meetings/conferences and are not yet published in full.						
**Brivaracetam (BRV)**						
BRV	(Biton *et al.*, 2014)	**RCT** Multi-centre, randomised, double-blind, placebo-controlled, Phase III trial	Adjunctive, partial-onset seizures	n=396	**NO** AE report	Irritability: 5% (vs 2% PBO)
BRV	(Ryvlin *et al.*, 2014)			N=398	**NO** AE report	Irritability: 5.1% (vs 2% PBO)
BRV	(Kwan *et al.*, 2014)			N=480	**NO** AE report	Irritability: 1.9% (vs 0% PBO)<br>Aggression: 1.4% (vs 0.8% PBO)
BRV	(French *et al.*, 2010)	**RCT** Multi-centre, randomised, double-blind, placebo-controlled, Phase IIb trial		N=208	**NO** AE report	None mentioned
BRV	(Van Paesschen *et al.*, 2013)			N=157	**NO** AE report	Irritability: 5.7% (vs. 3.8% PBO)
BRV	*(D'Souza et al., 2012)*	*Meta-analysis of the 5 RCTs above*	*Adjunctive, partial-onset seizures*	*N= 1639 (BRV N=1214)*	***NO*** *AE report*	*Non-psychiatric behavioural AEs: 6.8% (vs 4.2% PBO)*
**Carbamazepine (CBZ)**						
CBZ	(Shehata *et al.*, 2009)	**Observational** (Case–control)	Monotherapy in seizure-free epilepsy patients, vs. untreated epilepsy patients, vs. healthy controls	N=137 (N=45 on CBZ or VPA; N=34 untreated epilepsy patients; N=58 healthy controls)	**YES (ABS)**	Dose of AED was correlated with worsening verbal + non-verbal aggression; duration of intake correlated with worsening non-verbal aggression
CBZ	(Wieshmann *et al.*, 2011)	**Observational** (Audit of cases at a Neurology unit)	Patients with epilepsy taking AEDs ≥4 weeks	N=119 (CBZ N=36)	**NO (AEP)**	Feelings of anger, 16% (vs 33% LEV, 19% VPA, 15% LTG)
CBZ	(Hessen *et al.*, 2007)	**RCT** Randomised, double-blind, placebo-controlled study of AED withdrawal	Monotherapy, seizure-free epilepsy patients	N=115 (CBZ N=39 non-withdrawal, N= 37 withdrawal)	**YES (MMPI-2)**	Significant improvement with withdrawal vs. continuing AED, in depression (p=0.012) and brooding (p=0.027) subscales.
CBZ	(Pulliainen and Jokelainen, 1994)	Randomised, partially blinded, prospective study	Monotherapy, patients with epilepsy (aged 15–57 years)	N=64 (CBZ N=23; PHT N=20; control N=21)	**YES (POMS)**	Irritability improved with time after onset of treatment in both the CBZ and PHT group (p<004)
CBZ	(Friedman *et al.*, 1992)	**Observational**	Patients with mental retardation taking CBZ for seizures alone, for seizures and behavioural disturbance, or for behaviour alone	N=65 (CBZ+ epilepsy N=21)	**NO** (Subjective assessment and incidence of side effects)	No worsening in behaviour in the 20 patients taking CBZ solely for seizure control. Behaviour worsened in patients taking CBZ for psychiatric/behavioural disturbance
**Clobazam –** No relevant studies in adult populations were retrieved						
**Clonazepam (CLN)**						
CLN	(Lander *et al.*, 1979)	**Observational** (case series)	CLN monotherapy in 40 patients (33 with generalised seizures, 17 with focal)	N=40	**NO** AE incidence	Irritability: 7 patients (17%)
CLN	(Edwards, 1974)	**Review** of previous studies, and personal experience	Not specified	not specified	**NO** AE incidence	Irritability: ~20%
**Felbamate (FBM)**						
FBM	(Weintraub *et al.*, 2007)	**Observational** (retrospective chart review)	Adult outpatients (aged ≥16 years) with epilepsy, seen at a US epilepsy centre and treated with AED mono- and polytherapy.	N=1394 (FBM N=28)	**YES** Documentation of psychiatric side effects attributed to the AED, according to charts and physician notes.	Incidence of AED-attributed psychiatric/behavioural side effects was 3.6% with FBM (vs. 8.4% average of newer AEDs, 16% with LEV [highest] and 0.6% with GBP [lowest]). There were too few FBM patients for statistical comparison.<br>***[See text for detailed discussion]***
FBM	(Ettinger *et al.*, 1996)	**Observational** (prospective study with interviews and questionnaires)	Epilepsy patients receiving FMB (monotherapy and add-on)	N=60	**NO** AE incidence	Agitation or restlessness in 14 (23%), which caused discontinuation in 6 (12%)
FBM	(McConnell *et al.*, 1996)	**Observational** (selected case series)	Epilepsy patients with behavioural changes with FBM	N=7	**NO** Subjective description of behaviour	4 of the 7 patients had behavioural changes which included agitation/irritability
**Gabapentin (GBP)**						
GBP	(Weintraub *et al.*, 2007)	**Observational** (retrospective chart review)	Adult outpatients (aged ≥16 years) with epilepsy, seen at a US epilepsy centre and treated with AED monotherapy or polytherapy.	N=1394 (GBP N=160)	**YES** Documentation of psychiatric side effects attributed to the AED, according to charts and physician notes.	Incidence of AED-attributed psychiatric/behavioural side effects was lowest with GBP (0.6%), and significantly lower than average with the newer AEDs (8.4%, p<0.001). Rates of irritability (0.6%) and depression (0%) were significantly lower with GBP than average for other AEDs.<br>***[See text for detailed discussion]***
**Lacosamide (LCM) –** No relevant studies in adult populations were retrieved						
**Lamotrigine (LTG)**						
LTG	(Labiner *et al.*, 2009)	**RCT** (randomized, double-blind, parallel-group study of LTG vs LEV)	Adults with partial-onset seizures, with adjunctive LEV or adjunctive LTG	N=132 (LTG)<br>N=136 (LEV)	**YES Anger-Hostility subscale of POMS; IDAS**	Anger-Hostility subscale scores improved from baseline with adjunctive LTG, and significantly more than with adjunctive LEV. Anger/hostility improved with LTG at every time-point, and deteriorated with LEV at the majority of time-points.<br>LTG improved irritability (IDAS), whereas irritability worsened with LEV.

AED	Reference	Study type/design	Treatment and seizure type	Total N	Behaviour-specific measures used?	Prevalence of aggression-related behaviour
LTG	(Weintraub *et al.*, 2007)	**Observational** (retrospective chart review to explore psychiatric/behavioural effects of AEDs)	Adult outpatients (aged ≥16 years) with epilepsy, seen at a US epilepsy centre and treated with AED mono- and polytherapy.	N=1394 (LTG N=547)	**YES** Documentation of psychiatric side effects attributed to the AED, according to charts and physician notes.	Incidence of AED-attributed psychiatric/behavioural side effects was lower than average with LTG (4.8%, p<0.001), compared with 8.4% average for the newer AEDs. Rates of irritability (1.3%) and depression (1.5%) were significantly better (lower) with LTG than average for other AEDs. ***[See text for detailed discussion]***
LTG	(Huber *et al.*, 1998)	**Observational** (prospective open study)	Add-on LTG in patients with severe therapy-resistant epilepsy and multiple additional handicaps	N=125	**YES** **POM scale**	Positive psychotropic effect in 26% Negative psychotropic effect in 8% (mostly aggression)
LTG	(Kato *et al.*, 2011)	**Observational** (prospective open study)	Add-on LTG in Japanese patients with temporal lobe epilepsy	N=21	**YES** **BAA**	BAQ total score was significantly improved at Week 10 compared with baseline (p=0.002), and the anger subscale score was significantly improved (p=0.004)
LTG	(Chung *et al.*, 2007)	**Observational** (Retrospective review of retention rates and tolerability from patient records)	Patients with various seizure types, taking LTG, LEV, OXC, TPM, ZNS as mono- or polytherapy	N=828 (LTG, N=251)	**NO** Recorded side effects and reason for discontinuation	Behavioural side effects led to discontinuation in 8 pts taking LTG (3%), which was 12% of all patients who discontinued LTG. Behavioural AEs accounted for 40% of LEV discontinuations, 5% of OXC, 6% of TPM, and 10% of ZNS
LTG	(Faught *et al.*, 1999)	**Observational** (Prospectively planned chart review)	Add-on of LTG to VPA, patients with partial, generalised, and LGS seizures, followed up to 27 months	N=108	**NO** Side effect reporting	Irritability and agitation, classed as serious, was reported in 4 patients (4%) LTG was discontinued because of behaviour change in 1 patient (1/108, 1%)
LTG	(Beran and Gibson, 1998)	**Observational** (Prospective survey)	Patients with intellectual disability and refractory epilepsy	N=19	**YES** **Description of behaviour and changes**	10 patients developed/worsened aggressive behaviour after LTG add-on 4 patients had no change 4 patients had non-aggressive behaviour problems 1 patient's behaviour improved with LTG
LTG	(Ettinger *et al.*, 1998)	**Observational** (case series of patients with behaviour change)	Patients with epilepsy and mental retardation, who had significant behaviour changers with add-on LTG	N=7	**YES** **ABC**	Behavioural improvement 4/7 patients. Adverse behaviour change in 3/7 (irritability, temper/tantrums, self-injurious behaviour)
LTG	(Frey *et al.*, 2009)	**Observational** (retrospective case series of patients with end-of-dose withdrawal symptoms)	Long-term LTG monotherapy for epilepsy	N=6	**YES** **AEs (transient emergent psychological symptoms at end of LTG dose)**	From all patients treated with LTG by two physicians over 2 years, 6 were identified with possible transient end-of-dose symptoms of LTG withdrawal, which resolved on dosing. Symptoms included irritability, anxiety, trouble organising thoughts.
**Levetiracetam (LEV)**						
LEV	(Labiner *et al.*, 2009)	**RCT** (randomized, double-blind, parallel-group study of LTG vs LEV)	Adults with partial-onset seizures, with adjunctive LEV or adjunctive LTG	N=136 (LEV) N=132 (LTG)	**YES** **Anger-Hostility subscale of POMS; IDAS**	Anger-Hostility subscale scores improved from baseline with adjunctive LTG, and significantly more than with adjunctive LEV. Anger/hostility improved with LTG at every time-point, and deteriorated with LEV at the majority of time-points. LTG improved irritability (IDAS), whereas irritability worsened with LEV.
LEV	(Lee *et al.*, 2011)	**Observational** (prospective open-label study)	Korean patients with epilepsy, treated with add-on LEV	N=71	**YES** **BDI; BAI; SCL-90-R; SSIBeck; QOLIE-31**	Significant improvement from baseline in BAI, and some domains of SCL-90-R with LEV. Other scales/subscales showed no significant change (including no change in hostility subscale). 12 patients (17%) discontinued due to AEs, 5 of these due to psychiatric symptoms (nervousness, irritability, anxiety, hostility, depression, and suicidal ideation or attempt)
LEV	(Weintraub *et al.*, 2007)	**Observational** (retrospective chart review to explore psychiatric/behavioural effects of AEDs)	Adult outpatients (aged ≥16 years) with epilepsy , seen at a US epilepsy centre and treated with AED mono- and polytherapy.	N=1394 (LEV N=521)	**YES** Documentation of psychiatric side effects attributed to the AED, according to charts and physician notes.	Significantly higher incidence of psychiatric/behavioural side effects than average was found with LEV (16%), and significantly lower than average incidence with gabapentin (0.6%) and lamotrigine (4.8%). LEV patients had significantly more irritability (9.0%, P < 0.001) and behavioural change (3.5%, P < 0.001) than the average, and trend towards more depression. ***[See text for detailed discussion]***
LEV	(Helmstaedter *et al.*, 2008)	**Observational** (Case–control study with interviews and questionnaires of consecutive patients with epilepsy and their carers/relative)	Outpatients with epilepsy, taking either chronic LEV or other AEDs (monotherapy or polytherapy), and a spouse, parent, or significant other	N=288 (Taking chronic LEV patients N=288, LEV carers N=135; taking other AEDs and never taken LEV N=43)	**YES** Structured interview (direction of behaviour change overall and across 10 sub-categories), **FPZ**, **BIS-11**	Negative behaviour change in 33% (very negative in 12%), positive change in 22%, no change in 41% of patients taking LEV. Behaviour change only reported in 9% of controls (no LEV). Most common negative behaviour change was aggression.

AED	Reference	Study type/design	Treatment and seizure type	Total N	Behaviour-specific measures used?	Prevalence of aggression-related behaviour
LEV	(Helmstaedter *et al.*, 2013)	**Genetic** (candidate gene association study)	Epilepsy patients taking LEV as monotherapy or polytherapy	N=208 (stage I), N=100 (stage II)	**YES** Stage I: patient-rated psychotropic AEs attributed to LEV intake. Stage II: presence/absence of psychotropic AEs, based on clinical record review	Using patient data from Helmstaedter 2008, 38% of LEV patients had negative psychotropic AEs, and 22% had positive. Genetic polymorphisms in 3 genes were associated with negative psychiatric AEs, all related to dopamine signalling. 1 was confirmed in stage 2. Hypothesis: Reduced dopamine activity is associated with increased susceptibility to negative psychotropic effects of LEV.
LEV	(Ciesielski *et al.*, 2006)	**Observational** (open, prospective, non-randomised comparative short-term trial, comparing baseline performance to 7-days after add-on AED	Adults with refractory partial epilepsy, treated with add-on PGB or LEV (treatment selection based on clinical judgement)	N= 20 (PGB N=10; LEV N=10)	**YES** **Several neuro-**psychological tests, and two anxiety questionnaires	No major differences between drugs. Some improvement in behaviour with LEV (less insecurity, improved depression score, and improved anxiety vs. baseline) However, no aggressive-behaviour-specific scales were used, and only 1 week of treatment.
LEV	(M. Mula *et al.*, 2003)	**Observational** (Prospective chart review)	Adults with epilepsy treated with LEV (Partial and generalised epilepsies)	N=517	**YES** **Evaluation of psychiatric AEs at each visit**	53/517 patients developed psychiatric AEs (PAEs; 10.1%), most commonly aggressive behaviour (n=19, 3.5%). Risk factors for PAEs were history of febrile convulsions, previous psychiatric history, and history of status epilepticus. Co-therapy with lamotrigine reduced risk of PAEs.
LEV	(Mula *et al.*, 2004)	**Observational** (Prospective chart review)	Adults with epilepsy treated with LEV. who also had learning difficulties	N=118	**YES** **Evaluation of psychiatric AEs at each visit**	15 patients developed psychiatric adverse events (PAEs; 12.7%), most commonly aggression (7.6%). LEV was discontinued due to PAEs in 8.5%. No relationship between PAEs and seizure control was found. Previous psychiatric history and history of status epilepticus were risk factors for PAEs.
LEV	(Mula *et al.*, 2007)	**Observational** (Case registry analysis)	Patients who had a treatment trial with both TPM and LEV	N=108 patients	**YES** **Case registry recording/ assessing psychiatric AEs**	9 patients (8.3%) developed PAEs with both TPM and LEV; 5 had PAEs with LEV only (4.6%), 23 had PAEs with TPM only (22%); and 71 patients (66%) did not develop PAEs with either drug. (Overall, 14/108 patients, 13% had PAEs with LEV, 32/108, 30% with TPM). Risk factors for developing PAEs with both AEDs were previous psychiatric history, family psychiatric history, and history of febrile convulsions.
LEV	(White *et al.*, 2003)	**Observational** (case–control study of patients discontinuing LEV due to behavioural abnormalities)	Patients treated with LEV between 2000 and 2002	N=553 2 controls were selected for every index case of LEV discontinuation due to behavioural reasons.	**NO** Causes of discontinuation determined by chart review	The most common reason for LEV discontinuation was behavioural abnormalities: 38 patients (6.9%; depression in 16, irritability in 14, aggression in 5, psychosis/hallucination in 2). Ten patients (1.8%) were considered a danger to themselves or others. Variables associated with discontinuation were LEV faster titration, history of psychiatric disorder, and symptomatic generalised epilepsy.
LEV	(Wieshmann and Baker, 2013)	**Observational** (AED register analysis, of self-reported feelings of anger)	Patients with epilepsy taking LEV or other AEDs, and enrolled in the UK AED register; and non-AED-medicated controls	N= 418 (LEV N=158; other AEDs N=260)	**NO** LAEP (including questions on anger and depression)	% of patients reporting anger as sometimes or always a problem: LEV: 49% (monotherapy = 48%, polytherapy = 50%) no LEV: 39% control: 7%
LEV	(Wieshmann *et al.*, 2011)	**Observational** (Audit of cases at a Neurology unit)	Patients with epilepsy taking AEDs ≥4 weeks	N=119 (LEV N=12)	**NO** LAEP	% of patients reporting anger as sometimes or always a problem: LEV: 33% (vs. 16% CBZ, 19% VPA, 15% LTG)
LEV	(Mula *et al.*, 2015)	Observational (Consecutive sample questionnaire)	Adults with epilepsy treated with epilepsy	N=163	**NO** LAEP	9.8% of patients reported aggression as 'always' a problem. Aggression at any frequency was associated with 7-fold increased risk of being depressed.
LEV	(Mbizvo *et al.*, 2014)	**Meta-analysis** (of randomised controlled trials)	10 randomised placebo controlled trials of add-on LEV in patients with epilepsy (8 studies in adults, 2 studies in children)	N=1831	**NO** Only included AEs reported in original studies above the reporting thresholds (5% or 10%), so may exclude AEs below these thresholds.	**Hostility: 12% with LEV** vs. 6% with PBO in 1 RCT, but overall rate = 1.1% vs 0.81% **Aggression: 8%** vs 3% in 1 RCT, but overall rate =0.73% **Agitation: 3% and 6%** vs 0% and 1% in 2 RCTs, but overall rate=0.82% vs 0.14% **Irritability: 5%** vs 0% in 1 RCT, overall rate 0.46% **Abnormal behaviour: 5%** vs 0% in 1 RCT, overall rate 0.46% **Altered mood: 4%** vs 0% in 1 RCT, overall rate 0.37%
LEV	(French *et al.*, 2001)	**Systematic review/meta-analysis** (of randomised controlled trials)	All patients in the LEV safety database, including healthy volunteers, patients with refractory partial-onset seizures, elderly patients in cognitive studies, and patients with anxiety disorder.	N=3347 (Epilepsy: N=394 LEV; N=344 PBO)	**NO** AE reporting	**Epilepsy vs. other conditions:** Behavioural AEs were more common in epilepsy patients than in cognitive and anxiety patients, and more common with LEV vs PBO. Rates in placebo-controlled trials: Epilepsy: 13.5% LEV; 6.0% PBO Cognition: 6.3% LEV; 4.1% PBO Anxiety: 5.2% LEV; 5.5% PBO

AED	Reference	Study type/design	Treatment and seizure type	Total N	Behaviour-specific measures used?	Prevalence of aggression-related behaviour
LEV	(Cramer *et al.*, 2003)	**Systematic review/meta-analysis** (of randomised controlled trials)	Patients with refractory partial-onset seizures taking adjunctive LEV in short-term placebo-controlled trials and long-term open-label extensions, and also taking LEV in non-epilepsy trials	Epilepsy trials, LEV N=1393, PBO N=439	**NO** AE reporting	**Nervousness: 7.3%** (vs 1.8%) **Hostility: 3.3%** (vs 0.9%) **Emotional lability: 3.0%** (vs 0.2%) **Agitation: 1.6%** (vs 0.2%) **All affective behavioural AEs:** 25.4% (vs 6.2%)
LEV	(Peltola *et al.*, 2009)	**RCT** (placebo-controlled, double-blind)	Patients with refractory partial-onset seizures taking adjunctive LEV 3000 mg/day vs. placebo for 12 weeks. Age range 12–68 (mean=34).	N=158 (LEV N=79, PBO N=79)	**NO** AE reports	**Irritability:** 5/77 (6.5%) vs 0% with placebo. *NOTE that this study excluded patients with a history of recurrent psychiatric disorder*
LEV	(Xiao *et al.*, 2009)	**RCT** (placebo-controlled, double-blind)	Chinese adults (≥17 years) with refractory partial-onset seizures taking adjunctive LEV extended release 1000 mg/day vs. placebo for 12 weeks	N=56 (LEV N=28, PBO N=28)	**NO** AE reports	**Agitation:** 3/28 (10.7%) vs 0% with placebo. *NOTE that this study excluded patients who had mental retardation or history of a serious psychiatric disorder in the past 5 years.*
LEV	(Chung *et al.*, 2007)	**Observational** (Retrospective review of retention rates and tolerability from patient records)	Patients with various seizure types, taking LTG, LEV, OXC, TPM, ZNS as mono- or polytherapy. Age range 17–89 (mean=38.5)	N=828 (LEV N=196)	**NO** Recorded side effects and reason for discontinuation	Retention rate was 53.6% for LEV (highest retention was with LTG, 74.1% and lowest with topiramate, 44.2%). Behavioural side effects led to discontinuation in 38 patients taking LEV (19.4% of total, and 40.4% of the patients discontinuing). For comparison, OXC had fewest discontinuations due to behavioural AEs (2.1%) and the net highest after LEV was zonisamide (7.0%). Behavioural AEs accounted for just 5% of OXC discontinuations, 6% of TPM, and 10% of ZNS.
LEV	(Kang *et al.*, 2013)	**Observational** (retrospective electronic chart review, long-term treatment with LEV)	Patients treated with LEV at a tertiary epilepsy centre (generalised and focal epilepsies, mono- and polytherapy). Age range 14–79 years (mean=33).	N=568 treated with LEV either in monotherapy or add-on	**NO** Recorded side effects and reason for discontinuation	**Irritability (24%)** was the most common AE, and 28 patients discontinued as a result (4.9% of total, 51% of patients who discontinued) . Irritability was seen in **41%** of the 34 patients with a history of mood disorders (n=14).
LEV	(Steinhoff *et al.*, 2005)	**Observational** (16-week, open-label, Phase IV trial)	Adult patients (≥16 years) with refractory focal epilepsy treated with adjunctive LEV at epilepsy centres in Germany, Austria and Switzerland.	N= 178	**NO** Recorded side effects and reason for discontinuation	**Hostility** (5.1%, n=9) was the only behavioural AE reported (≤5% threshold), was mild in 7 cases, moderate in 2, and was considered related to LEV in all 9 cases. *NOTE that this study excluded patients with a history of serious psychiatric disorders within the past 5 years.*
LEV	(Ben-Menachem and Gilland, 2003)	**Observational** (prospective, open-label study)	Patients with refractory seizures (no limits on seizure type or previous medical history). Age range 12–68 (mean=39).	N=98 Included 10 patients with previous serious psychiatric history and 22 mentally handicapped patients.	**NO** Side effects were determined from patient diaries	**Irritation/aggression (12%**, n=12), and led to discontinuation in 4 patients (4.1% of total). 3 of the patients who discontinued had previous behavioural problems and mental retardation.
LEV	(Bootsma *et al.*, 2007)	**Observational** (retrospective file study)	Patients treated with LEV at an epilepsy centre in The Netherlands. Age range 1–75 years (mean=33), and 20% were aged <18 years. LEV was used as mono- and polytherapy across several focal and generalised seizure types.	N=301 33% of patients had mental retardation	**NO** Adverse events derived from physician notes	**Mood disorders: 5.0%--10.6%** at time points up to 18 months, most commonly "activating" AEs (aggression, hyperirritability, and agitation) **Positive behaviour: 6.3–7.4%** Discontinuation was associated with aggression/hyperirritability/agitation in 5.6% of patients (17/301), and was more common in patients with mental handicap than without.
LEV	(Stephen *et al.*, 2011)	**Observational** (retrospective audit)	Patients prescribed LEV as monotherapy at an epilepsy centre in the UK. Age range 12–81 years. Several focal and generalised seizure types.	N=228; all on monotherapy	**NO** Side effects were derived from patient diaries and physician notes	Neuropsychiatric symptoms (aggression, mood swings, irritability, depression) led to discontinuation in 18 patients (7.9%), accounting for 49% of all discontinuations. Discontinuations due to individual side effects (some patients reported more than 1 side effect at discontinuation): Aggression: n=10 (4.4%) Mood swings: n=7 (3.2%) Irritability: n=3 (1.3%) Depression: n=2 (0.9%)
LEV	(Belcastro *et al.*, 2008)	**Observational** (prospective open-label flexible dosing study)	Patients aged ≥65 with late-onset post-stroke seizures	N=35	**NO** Presence of side effects determined using a checklist that included behavioural symptoms	3 patients discontinued due to severe AEs related to aggression (8.6%): n=1: aggressive behaviour within first 2 months n=2: severe psychomotor agitation with aggressive behaviour
LEV	(Weber and Beran, 2004)	**Open-label** (prospective open label pilot study)	Patients with PGE or LGS. Age range 31–48 years.	N=10	**NO** AE reporting	1 patient with LGS withdrew due to aggression (10%).

AED	Reference	Study type/design	Treatment and seizure type	Total N	Behaviour-specific measures used?	Prevalence of aggression-related behaviour
LEV	(Dinkelacker *et al.*, 2003)	**Observational** (case series)	Patients with aggressive behaviour with LEV treatment	N=33	**NO** Case history	Aggression seen in ~3.5% of patients overall. In these 33 cases, aggression resolved in 24, but was severe in the remaining 9 patients, including physical violence. In 2 cases, emergency psychiatric treatment was needed. In 4 of these 9 patients, there was a history of irritability or aggression, and 1 patient had experienced aggressiveness with gabapentin treatment.
LEV	(Eisenschenk *et al.*, 2014)	**Case study**	VAL+LEV	N=1	**NO**	Case report of a homicide following a cluster of seizures and post-ictal psychosis. The patient was taking VAL+LEV at the time of the homicide, which changed 9 months prior from VAL+CBZ.
**Oxcarbazepine (OXC)**						
OXC	(Weintraub *et al.*, 2007)	**Observational** (retrospective chart review to explore psychiatric/behavioural effects of AEDs)	Adult outpatients (aged ≥16 years) with epilepsy, seen at a US epilepsy centre and treated with AED mono- and polytherapy.	N=1394 (OXC N=162)	**YES** Documentation of psychiatric side effects attributed to the AED, according to charts and physician notes.	Incidence of AED-attributed psychiatric/behavioural side effects was 5.6% with OXC (vs. 8.4% average of newer AEDs, 16% with LEV [highest] and 0.6% with GBP [lowest]). Rates of irritability (0%) were significantly better (lower) with OXC than average for other AEDs. ***[See text for detailed discussion]***
OXC	(Chung *et al.*, 2007)	**Observational** (Retrospective review of retention rates and tolerability from patient records)	Patients with various seizure types, taking LTG, LEV, OXC, TPM, ZNS as mono- or polytherapy. Age range 17–89 (mean=38.5)	N=828 (OXC N=97)	**NO** Recorded side effects and reason for discontinuation	Retention rate was 58.8% for OXC (highest retention was with LTG, 74.1% and lowest with topiramate, 44.2%). OXC had fewest discontinuations due to behavioural side effects (2 of 97 patients) of all AEDs tested, and LEV had the highest (38 of 196 patients, 19%).
**Perampanel (PMP)**						
PMP	(French *et al.*, 2012)	**RCT** Multicentre, randomized, double-blind, placebo-controlled, Phase III trial	Adjunctive PMP, patients with refractory partial-onset seizures (aged ≥12 years)	N=388 (PMP N=267)	**NO** (Adverse event reporting)	Irritability AE: 14.2% with 12 mg (vs 5% with PBO) Aggression led to discontinuation in 1.9% of patients with 8 or 12 mg perampanel (vs 0 with PBO) Psychiatric SAEs: 3.7% with 12 mg (vs 1.7% with PBO)
PMP	(French *et al.*, 2013)			N=386 (PMP N=250)	**NO** (Adverse event reporting)	Irritability AE: 9.2% Aggression AE: 1.6% with 12 mg (vs <1% with PBO) Anger AE: 1.7% with 12 mg (vs 0% with PBO) No aggression-related SAEs
PMP	(G L Krauss *et al.*, 2012)			N=706 (PMP N=521)	**NO** (Adverse event reporting)	No aggression-related AEs reported
PMP	(Steinhoff *et al.*, 2013)	**RCT** Pooled analysis of the 3 studies above	Patients with refractory partial-onset seizures (aged ≥12 years)	N=1478 (PMP N=1038)	**NO** (Adverse event reporting)	Irritability AE: 11.8% with 12 mg (vs 2.9% with PBO) Aggression AE: 3.0% with 12 mg (vs 1.0%) Psychiatric SAE: 12/1038 (1.2%) with 12 mg (vs 0.9%) Aggression SAE: 3/1038 (0.29% with 12 mg (vs 0 with PBO)
PMP	(Gidal *et al.*, 2013)	**RCT** Pooled PK/PD analysis of the 3 Phase III studies	Patients with refractory partial-onset seizures (aged ≥12 years) and PK data	N=1109 (PMP N=770)	**NO** (Adverse event reporting)	Mean predicted probability of irritability increased with plasma concentrations of perampanel (p<0.001 vs lower plasma concentrations) Probability was increased in patients coadministered phenobarbital.
PMP	(Krauss *et al.*, 2014)	**RCT/open-label** Open-label extension to three Phase III trials, with exposure of up to 3.3 years	Patients with refractory partial-onset seizures (aged ≥12 years), continuing into extension phase	N=1216, all PMP-treated	**NO** (Adverse event reporting)	Incidence during 1803 patient-years: Irritability AE: 11.5% Aggression AE: 5.1% Aggression SAE: 1.0% (12/1216) Agitation SAE: 0.2% (2/1206) Psychiatric SAE: 3.9% (47/1216)
PMP	(G. L. Krauss *et al.*, 2012)	**RCT** Two randomised, double-blind, placebo-controlled Phase II trials	Adults with refractory partial-onset seizures (aged 18–70 years)	N=153 (PMP N=102) N=48 (PMP N=38)	**NO** (Adverse event reporting)	No aggression-related AEs reported
PMP	(Hsu *et al.*, 2013)	**Meta-analysis** Of five trials in epilepsy (see above)	Patients with pharmacoresistant partial-onset seizures (aged ≥12 years)	N=1678 (PMP N=1176)	**NO** (Adverse event reporting)	Perampanel not significantly associated with any aggression-related AEs. However, analysis could only include studies that reported aggression-related AEs Risk ratios vs placebo (95% CIs): Irritability: 2.19 (0.93–5.14) Aggression: 1.63 (0.17–15.54) Anger: 2.73 (0.13–56.44)
PMP	(Zaccara *et al.*, 2013)	**Meta-analysis** Of five trials in epilepsy and four in Parkinson's disease	Patients with pharmacoresistant epilepsy (aged ≥12 years) and patients with Parkinson's disease	N=3947 (PMP N=2627)	**NO** (Adverse event reporting)	Irritability AEs (irritability + aggression, merged) were significantly associated with 12 mg dose (not other individual doses). Risk difference vs. placebo = 0.09 (99% CI 0.02–0.16, p<0.001)
PMP	(LoPresti *et al.*, 2014). Now published in full (Ettinger *et al.*, 2015)	***RCT post-hoc analysis*** *(exploration of behavioural AEs in three Phase III studies)*	*Patients with pharmacoresistant partial-onset seizures (aged ≥12 years)*	*N=1480 (PMP N=1038)*	***NO*** *(Adverse event reporting)*	*OVERALL, PMP total vs placebo* *Irritability: 7.0% (vs 2.9%)* *Aggression: 1.6% (vs 0.5%)* *Anger: 1.2% (vs 0.2%)* *Agitation: 0.4% (vs 0.5%)* *ADULTS (excluding patients aged ≤17)* *Aggression: 1.3% (vs 0.5%)*

AED	Reference	Study type/design	Treatment and seizure type	Total N	Behaviour-specific measures used?	Prevalence of aggression-related behaviour
PMP	(Ettinger *et al.*, 2014). Now published in full (Ettinger *et al.*, 2015)	***RCT post-hoc analysis*** *(exploration of behavioural AEs in all perampanel clinical trials)*	*Healthy volunteers, epilepsy populations, and non-epilepsy (Parkinson's, neuropathic pain, multiple sclerosis, migraine)*	*N=4368 PMP-treated subjects*	***NO*** *(Adverse event reporting)*	*Epilepsy Phase III trials*<br>*Rates as reported in Fain et al. AES 2014.*<br>*Non-epilepsy trials*<br>*No increase in aggression-related AEs. Rates not reported, as below the 0.2% threshold*<br>*All trials*<br>*Homicidal ideation/threat in 4 of 4368 PER-treated patients (0.1%)*
PMP	(Fain *et al.*, 2015).	***RCT post-hoc analysis*** *(aggression AEs in Phase III epilepsy studies, by levetiracetam use)*	*Patients with pharmacoresistant partial-onset seizures (aged ≥12 years)*	*N=1480 (PMP, no LEV N=728; PMP with LEV N=310)*	***NO*** *(Adverse event reporting)*	*No increased liability for hostility/aggression AEs with concomitant LEV vs no concomitant LEV*<br>*Irritability: 13.2% vs 11.3%*<br>*Aggression: 1.9% vs 1.5%*
PMP	(French *et al.*, 2015)	**RCT** Multicentre, randomised, double-blind, placebo-controlled, Phase III trial	Adjunctive treatment of patients with pharmacoresistant PGTC seizures in idiopathic generalised epilepsy	N=162 (PMP N=81)	**NO** (Adverse event reporting)	Irritability AE: 11.1% (vs 2.4% with PBO)<br><br>All hostility/aggression-related AEs:<br>-Broad and narrow SMQ terms: 18.5% with perampanel, vs 4.9% with placebo<br>-Just narrow SMQ terms: 2.5% with perampanel, *vs* 0% with placebo
PMP	(Steinhoff *et al.*, 2014)	**Observational** (Multicentre prospective survey of clinical experience over 6 months)	Patients prescribed adjunctive PMP at tertiary epilepsy centres for refractory seizures (mean age 39 years, range 12–84)	N=281	**NO** (Adverse event reporting)	Aggression: 2.8%<br>Irritability: 2.1%
PMP	(Coyle *et al.*, 2014)	**Observational** (Retrospective analysis)	Adults prescribed adjunctive PMP for refractive epilepsy	N=47	**NO** (Adverse event reporting)	Behavioural AEs were the most common reason for discontinuation of PMP: aggressive behaviour (2/47); suicidal ideation (2/47); both (1/47)
PMP	(Villanueva *et al.*, 2015)	***Observational*** *(Multicentre, retrospective audit of clinical experience)*	*Patients prescribed adjunctive PMP at tertiary epilepsy centres for refractory seizures (mean age 38 years)*	*N=111*	***NO*** *(Adverse event reporting)*	*Irritability: 13.5%*<br>*Irritability and aggression were more frequent in patients with a comorbid psychiatric condition.*
PMP	*(Goulding et al., 2014)*	***Observational*** *(Prospectively planned audit)*	*Patients prescribed adjunctive PMP for refractory partial-onset seizures*	*N=39*	***NO*** *(Adverse event reporting)*	*Anger or aggression: 10%*
PMP	(Juhl and Rubboli, 2014)	***Observational*** *(Audit of clinical experience)*	*Adults prescribed adjunctive PMP for drug-resistant focal epilepsy*	*N=22*	***NO*** *(Adverse event reporting)*	*Aggressiveness: 8/22 (36%)*
PMP	(Kottmel *et al.*, 2014)	***Observational*** *(Audit of clinical experience)*	*Patients prescribed adjunctive PMP for refractory focal seizures*	*N=38*	***NO*** *(Adverse event reporting)*	*Discontinuation due to irritability: 4/38 (10.5%)*
PMP	*(Greshake and Straub, 2014)*	***Observational*** *(Audit of clinical experience)*	*Adults prescribed adjunctive PMP for pharmacoresistant epilepsy*	*N=60*	***NO*** *(Adverse event reporting)*	*Behaviour disturbance: 5/60 (8.3%)*
PMP	*(Sieradzan and Hodgson, 2014)*	***Observational*** *(Retrospective analysis)*	*Adults prescribed adjunctive PMP for refractory partial epilepsy at a tertiary epilepsy centre*	*N=60*	***NO*** *(Adverse event reporting)*	*Behavioural disturbance: 8%*
PMP	*(Parrett et al., 2014)*	***Observational*** *(Retrospective data collection)*	*Adults prescribed adjunctive PMP in neurology practice*	*N=24*	***NO*** *(Adverse event reporting)*	*Behaviour disturbances: 4/24 (17%)*
PMP	*(Flynn and Delanty, 2014)*	***Observational*** *(Audit of electronic epilepsy patient record)*	*Adult patients prescribed PMP for severely refractory epilepsy*	*N=20*	***NO*** *(Adverse event reporting)*	*Mood/behavioural alteration: 6/20 (30%)*
PMP	*(Keogh et al., 2014)*	***Observational*** *(Retrospective audit of clinical experience)*	*Adults prescribed PMP for focal epilepsy*	*N=16*	***NO*** *(Adverse event reporting)*	*Behavioural disturbance: 37%*
PMP	*(Schmid and Huber, 2014)*	***Observational*** *(Audit of clinical experience)*	*Cognitively impaired, institutionalised adults with highly therapy-resistant epilepsy*	*N=26 (17 evaluable)*	***NO*** *(Adverse event reporting)*	*Discontinuation due to:*<br>*-mood instability (1/17, 5.9%)*<br>*-agitation (1/17, 5.9%)-*
PMP	*(Kelly et al., 2014)*	***Observational*** *(Prospective audit)*	*Patients prescribed adjunctive PMP for highly drug-resistant focal epilepsy*	*N=41*	***NO*** *(Adverse event reporting)*	*No aggression-related side effects reported*
PMP	(Dolton and Choudry, 2014)	**Case study**	A patient with moderate learning disability, challenging behaviour, and treatment-refractory epilepsy	N=1	**NO** (Clinical description)	Patient had a history of occasional challenging behaviour not associated with seizures. Increase in aggressive behaviour occurred, and seizure frequency/severity markedly decreased when PMP was added and increased to 8 mg (concomitant with gabapentin, zonisamide, and valproate). Behaviour continued when PMP dose was reduced. Patient could no longer live at home, and impact on social function was substantial.
**Phenytoin (PHT)**						
PHT	(Pulliainen and Jokelainen, 1994)	Randomised, partially blinded, prospective study	Newly diagnosed before and during monotherapy treatment with CBZ and PHT, compared with controls	N=64 (CBZ N=23; PHT N=20; control N=21)	**YES (POMS)**	Irritability improved with time after onset of treatment in both the CBZ and PHT group (p<004)

**AED**	**Reference**	**Study type/design**	**Treatment and seizure type**	**Total N**	**Behaviour-specific measures used?**	**Prevalence of aggression-related behaviour**
**Pregabalin (PGB)**						
PGB	(Ciesielski *et al.*, 2006)	**Observational** (open, prospective, non-randomised comparative short-term trial, comparing baseline performance to 7-days after add-on AED	Adults with refractory partial epilepsy, treated with add-on PGB or LEV (treatment selection based on clinical judgement)	N= 20 (PGB N=10; LEV N=10)	**YES** Several neuro-psychological tests, and two anxiety questionnaires	No major differences between drugs. Some improvement in behaviour with LEV (less insecurity, improved depression score, and improved anxiety vs. baseline). Tendency toward increase in hostility after PGB treatment. However, no aggressive-behaviour specific scales were used, and **only 1 week of treatment**.
PGB	(Valentin *et al.*, 2009)	**Observational** (audit of out-patients)	Out-patients prescribed PGB for refractory epilepsy as mono/polytherapy. Age range 21–84 years.	N=96	**NO** (side-effect reporting)	Irritability reported in 1 of 50 patients remaining on treatment at last follow-up. 1 patient discontinued due to irritability and cognitive side effects.
PGB	(Huber *et al.*, 2008)	**Observational** (retrospective evaluation)	Inpatients with refractory epilepsy and intellectual disability (adolescents and adults).	N=32	**NO** (side-effect reporting, against a list of 10 common AEs)	In 1 patient, increase in self-injurious behaviour was observed.
**Retigabine (RTG)** – No studies we identified included any reports of aggression-related behaviours						
**Tiagabine (TGB)**						
TGB	(Sveinbjornsdottir *et al.*, 1994)	**RCT** (open-label titration and fixed dose phase, with cross-over to double-blind/placebo-controlled phase)	Add-on tiagabine in patients with intractable epilepsy.	N=19	**YES** MACL and also AE reporting	No effect on mood or behaviour rating scales. Aggression/irritability was the most common AE (8/19 in the open-label titration, and 3/11 with TGB in the double-blind period, vs 0/11 with placebo). Three patients withdrew early due to aggression.
TGB	(Weintraub *et al.*, 2007)	**Observational** (retrospective chart review to explore psychiatric/behavioural effects of AEDs)	Adult outpatients (aged ≥16 years) with epilepsy, seen at a US epilepsy centre and treated with AED mono- and polytherapy.	N=1394 (TGB N=19)	**YES** Documentation of psychiatric side effects attributed to the AED, according to charts and physician notes.	Incidence of AED-attributed psychiatric/behavioural side effects was 15.8% with TGB (vs. 8.4% average of newer AEDs, 16% with LEV [highest] and 0.6% with GBP [lowest]). There were too few TGB patients for this high rate to reach statistical significance. Irritability (10.5%), depression (5.3%), anxiety (5.3%) and psychosis (5.3%) rates were high with TGB, but did not reach significance due to small numbers. ***[See text for detailed discussion]***
TGB	(Kaufman *et al.*, 2002)	**Observational** (case reports)	Case reports of 2 patients with postencephalitic epilepsy and impulse control disorder	N=2	**NO** Descriptive	In these two patients, tiagabine improved seizure control and behaviour (fewer aggressive outbursts), but led to increased irritability in 1 patient if dosed >20 mg/day.
**Topiramate (TPM)**						
TPM	(Weintraub *et al.*, 2007)	**Observational** (retrospective chart review to explore psychiatric/behavioural effects of AEDs)	Adult outpatients (aged ≥16 years) with epilepsy, seen at a US epilepsy centre and treated with AED mono- and polytherapy.	N=1394 (TPM N=112)	**YES** Documentation of psychiatric side effects attributed to the AED, according to charts and physician notes.	Incidence of AED-attributed psychiatric/behavioural side effects was 6.3% with TPM (vs. 8.4% average of newer AEDs, 16% with LEV [highest] and 0.6% with GBP [lowest]). Irritability (2.7%), depression (2.7%), and behavioural change (2.8%) were seen in some patients, but not significantly more than average. ***[See text for detailed discussion]***
TPM	(Marco Mula *et al.*, 2003)	**Observational** (retrospective file study)	The fist consecutive epilepsy patients treated with TPM at a tertiary epilepsy clinic.	N=431	**NO** Evaluation at each patient visit, and recording and describing psychiatry adverse events (PAEs).	PAEs reported in 103 of 431 patients (24%):<br>-Aggressive behaviour: 5.6% (with or without irritability)<br>-Other behaviour abnormalities: 3.9% (e.g. agitation, anger/hostility, anxiety)
TPM	(Mula and Trimble, 2003)	**Observational** (retrospective analysis)	Epilepsy patients who developed psychiatric AEs during topiramate treatment	N=103	**NO** Classified behavioural changes, and defined two behavioural dimensions	Of the 103 patients with PAEs:<br>-45% had affective disorder<br>-23% had aggressive behaviour<br>-16% had psychosis<br>-13% had anxiety<br>-4% had behavioural/personality changes<br>Aggressive behaviour resolved in 22/24 (most required discontinuation, some dose reduction).
TPM	(Mula *et al.*, 2007)	**Observational** (Case registry analysis)	Patients who had a treatment trial with both TPM and LEV	N=108 patients	**YES** Case registry recording/ assessing psychiatric AEs	9 patients (8.3%) developed PAEs with both TPM and LEV; 5 had PAEs with LEV only (4.6%), 23 had PAEs with TPM only (22%); and 71 patients (66%) did not develop PAEs with either drug. (Overall, 14/108 patients, 13% had PAEs with LEV, 32/108, 30% with TPM). Risk factors for developing PAEs with both AEDs were previous psychiatric history, family psychiatric history, and history of febrile convulsions.
TPM	(Chung *et al.*, 2007)	**Observational** (Retrospective review of retention rates and tolerability from patient records)	Patients with various seizure types, taking LTG, LEV, OXC, TPM, ZNS, as mono- or polytherapy. Age range 17–89 (mean=38.5)	N=828 (TPM N=156)	**NO** Recorded side effects and reason for discontinuation	Retention rate was 44.2% for TPM (highest retention was with LTG, 74.1% and lowest with topiramate). TPM had few discontinuations due to behavioural side effects (5 of 156 patients), only OXC had fewer. LEV had the highest (38 of 196 patients, 19%).

AED	Reference	Study type/design	Treatment and seizure type	Total N	Behaviour-specific measures used?	Prevalence of aggression-related behaviour
TPM	(Bootsma *et al.*, 2004)	**Observational** (Long-term, retrospective file study, up to 10 years follow-up)	All patients with refractory epilepsy treated with TPM at an epilepsy centre in 1993, up to 2002. Mean age 34.9, range 1–73 years.	N=470	**N** Side-effect reporting by patients and neurologists	Together, side effects of agitation, aggression, and hyperirritability were seen in 5.7% of patients at 18 months, and led to discontinuation in 32 patients (11.9% of the 269 who discontinued).
TPM	(Stephen *et al.*, 2000)	**Observational** (Prospective case review)	Patients with refractory epilepsy treated with TPM at an epilepsy centre. Age range 18–75 years.	N=170	**NO** Side-effect reporting	TPM was withdrawn in 51 patients<br>-due to irritability in 9 patients (5%)<br>-due to agitation in 2 patients<br>In the 46 patients continuing on TPM, 3 had side effects of irritability.
TPM	(Voronkova *et al.*, 2007)	**Observational**	Patients treated with topiramate at Russian Neurology centre; range of epilepsy types and ages from early childhood to elderly.	N=114 (N=39 adults)	**NO** Side-effect reporting	Irritability seen in 1 patient (<1%); improved behaviour in 1 patient (ages not specified).
TPM	(Tartara *et al.*, 1996)	**Observational** (Long-term, prospective, open, study)	Patients with refractory partial epilepsy or LGS. Age range 19–48 years.	N=15	**NO** Side-effect reporting	Irritability seen in 3 of 15 patients (20%), and led to discontinuation in 1 of these patients (7%).
**Valproate (VPA)**						
VPA	(Shehata *et al.*, 2009)	**Prospective** (Non-randomised, controlled, study)	Newly diagnosed and untreated epileptic patients vs treated epileptic patients, aged 18–45 years.	N=137, total. Treated, N=34 (CBZ=25; VPA=13); untreated, N=45; controls, N=58.	**YES** ABS (validated Arabic version)	Aggression was significantly worse in both treated and untreated patients vs healthy controls. Aggression was significantly worse in patients treated with VPA vs CBZ.
VPA	(Ruuskanen *et al.*, 1979)	**Retrospective** (Clinical record review)	Inpatients with severe refractory epilepsy, prescribed sodium valproate in June 1978	N=55	**NO** Side-effect reporting	Aggression in 1 patient (1.8%). No discontinuations due to side effects.
**Vigabatrin (VGB)**						
VGB	(Weintraub *et al.*, 2007)	**Observational** (retrospective chart review to explore psychiatric/behavioural effects of AEDs)	Adult outpatients (aged ≥16 years) with epilepsy, seen at a US epilepsy centre and treated with AED mono- and polytherapy.	N=1394 (VGB N=13)	**YES** Documentation of psychiatric side effects attributed to the AED, according to charts and physician notes.	Incidence of AED-attributed psychiatric/behavioural side effects was 0% with VGB (vs. 8.4% average of newer AEDs, 16% with LEV [highest]), but with too few patients (N=13) to state this as a definitive rate. ***[See text for detailed discussion]***
VGB	(Loiseau *et al.*, 1986)	**RCT** (Randomised, double-blind, placebo-controlled, cross-over trial)	Patients aged 10–58 years old with refractory complex partial seizures or generalised epilepsy	N=23	**NO** Subjective adverse event reporting	Irritability: Reported in 2 patients during placebo phase, and 1 during VGB phase.
VGB	(Levinson and Devinsky, 1999)	**RCT** (Pooled analysis of VGB double-blind, placebo-controlled trials)	Patients with treatment-resistant partial epilepsy, receiving add-on VGB	N=1434 (VGB, N-812)	**NO** Data extracted from adverse event reporting	Odds ratio vs. placebo (95% CI)<br>Aggressive reaction: 1.9 (0.6–5.4), p=ns<br>Agitation: 1.4 (0.8–2.3), p=ns
VGB	(Thomas *et al.*, 1996)	**Case series** (Follow-up of patients reporting behavioural problems with VGB to either the authors or the manufacturers)	Follow-up of 81 of the 136 patients reporting behavioural problems	N=81	**NO** (review of clinical information)	22 patients met criteria for depression<br>28 patients met criteria for psychosis<br>No mention of aggression-related behaviour.
VGB	(Robinson *et al.*, 1990)	**Case series** Report of behavioural reactions among patients treated with VGT	Adjunctive use, patients with refractory epilepsy	N=119	**NO** Case report	8 patients became both physically and verbally aggressive, beginning 5 weeks from start of treatment.<br>Most patients had a history of aggression.
VGB	(Armour *et al.*, 1992)	**Observational** (Open study of vigabatrin in adults with uncontrolled epilepsy and severe learning difficulties)	Adjunctive use, various refractory seizure types	N=22	**NO** (Adverse event reporting)	Adverse events possibly/probably related to vigabatrin treatment:<br>Aggression: 4 of 22<br>Agitation: 2 of 22
**Zonisamide (ZNS)**						
ZNS	(Carmichael *et al.*, 2013)	**RCT** (meta-analysis of RCTs with add-on zonisamide)	Patients with drug-resistant partial epilepsy, aged ≥12 years.	N=949	**NO** Adverse event reporting	Zonisamide use was significantly associated with agitation/irritability (relative risk 2.35, 95% CI 1.05–5.27). Total events: 33 (ZNS) vs 12 (placebo)
ZNS	(Sackellares *et al.*, 2004)	**RCT** (Double-blind, placebo-controlled trial)	Adult patients with drug-resistant partial epilepsy	N=152 (ZNS N=78)	**NO** (Adverse event reporting)	Irritability was one of the most frequently reported AEs (rate not given).
ZNS	(Weintraub *et al.*, 2007)	**Observational** (retrospective chart review to explore psychiatric/behavioural effects of AEDs)	Adult outpatients (aged ≥16 years) with epilepsy, seen at a US epilepsy centre and treated with AED mono- and polytherapy.	N=1394 (ZNS N=192)	**YES** Documentation of psychiatric side effects attributed to the AED, according to charts and physician notes.	Incidence of psychiatric/behavioural side effects was 9.9% with ZNS (vs. 8.4% average of newer AEDs, 16% with LEV [highest] and 0.6% with GBP [lowest]). ***[See text for detailed discussion]***
ZNS	(Chung *et al.*, 2007)	**Observational** (Retrospective review of retention rates and tolerability from patient records)	Patients with various seizure types, taking LTG, LEV, OXC, TPM, ZNS, as mono- or polytherapy. Age range 17–89 (mean=38.5)	N=828 (ZNS N=128)	**NO** Recorded side effects and reason for discontinuation	Retention rate was 60.2% for ZNS (highest retention was with LTG, 74.1% and lowest with topiramate 44.2%).<br>ZNS had few discontinuations due to behavioural side effects (5 of 128 patients), only TPM and OXC had lower rates. LEV had the highest (38 of 196 patients, 19%).

**AED**	**Reference**	**Study type/design**	**Treatment and seizure type**	**Total N**	**Behaviour-specific measures used?**	**Prevalence of aggression-related behaviour**

Studies shown in italics have been presented at meetings/conferences and are not yet published in full.
Abbreviations:
ABC: Aberrant Behavioural Checklist; ABS: Aggressive Behaviour Scale; AEP: Liverpool Adverse Event Profile; BAI: Beck Anxiety Inventory; BAQ: Buss-Perry Aggression Questionnaire; BDI: Beck Depression Inventory; BIS-11: Barratt Impulsiveness Scale-11; CI: confidence interval; FPZ: Fragebogens zur Persönlichkeit bei zerebralen Erkrankungen (assesses the aspects of personality in central nervous system-related diseases); IDAS: Irritability-Depression-Anxiety Scale; LGS: Lennox–Gastaut Syndrome; MACL: Mood Adjunctive Check List; MMPI-2: Minnesota Multiphasic Personality Inventory 2; PGE: primary generalised epilepsy; POMS: Profile of Mood States; QOLIE-31: Quality of Life in Epilepsy Inventory-31; SCL-90-R: 30 Symptom Checklist-90-Revised; SSIBeck: Scale for Suicide Ideation-Beck.

C. Supplemental Table S-2: Summary of the evaluated studies for each AED (in children and adolescents)

AED	Reference	Study type/design	Treatment stage and seizure type	Total N	Outcome measure	Prevalence
**Clobazam (CLB)**						
CLB	(Paolicchi *et al.*, 2015)	**RCT**<br>Randomised, double-blind, placebo-controlled trial	CLB or placebo add-on therapy patients aged ≤18 years with LGS.<br>CLB low-dose (0.25mg/kg), medium-dose (0.5mg/kg), or high-dose (1.0mg/kg)	N=194<br>(CLB=146; placebo=48)	**YES**<br>CBCL<br>Also AE reporting	**CBCL** results:<br>No significant difference between CLB and PBO change from baseline in CBCL aggression scale. Trend towards a significant difference in patients with a prior history of aggression (*P*=0.287)<br><br>All aggression-related AEs:<br>CLB: 23/146 (15.8%)<br>PBO: 4/48 (8.3%)<br>Individual AEs:<br>Aggression: 6.8% *vs* 4.2%<br>Irritability: 7.5% *vs* 4.2%
CLB	(Sheth *et al.*, 1994, 1995)	**Observational**<br>(Prospective, open-label)	CLB mono- or add-on therapy in refractory epilepsy of various seizure types and aetiologies	N=63 treated with clobazam, case series reported for the 7 patients with behavioural AEs	**NO**<br>Parental-report	"Aggressive agitation" in seven children (11.1%), including biting, kicking, head-banging, tantrums and hyperactivity
CLB	(Jan and Shaabat, 2000)	**Observational**<br>(Prospective, open, uncontrolled)	CLB add-on therapy for intractable epilepsy	N=31	**NO**<br>Side-effect reporting	Irritability and behavioural change reported in two patients. Three patients withdrawn due to vomiting or unspecified behavioural change
CLB	(Bawden *et al.*, 1999)	Prospective, randomised, blinded (double dummy) parallel group	CLB monotherapy for partial or generalised seizures (vs CBZ).	N=41	**NO**<br>Checklist of systemic and behavioural side effects; behavioural side effects characterised as internalising or externalising	Six CLB patients had behavioural side-effects (3 internalising, 3 externalising), vs. 5 taking standard monotherapy (2 externalising, 3 internalising).
CLB	(Klehm *et al.*, 2014)	**Observational**<br>(Retrospective chart review)	CLB add-on or monotherapy in children with epilepsy (generalised and focal)	N=300	**NO**<br>Adverse-event reporting	Mood/behavioural changes were reported in 23/300 patients (7.7%)
**Clonazepam (CLN)**						
CLN	(Lander *et al.*, 1979)	**Observational**	CLN monotherapy in children and adults with refractory epilepsy (ages 9–60 years)	N=40	**NO**<br>Side-effect reporting	7 patients (17%) had "undesirable effects", including irritability and aggression
CLN	(Kalachnik *et al.*, 2002)	**Systematic review of studies**	Various epilepsies, and psychiatric and other indications	N=446 (N=208 with epilepsy)	**NO**	Prevalence of behavioural disturbance in individuals with epilepsy was 15.4% (across all benzodiazepines)
CLN	(Mikkelsen *et al.*, 1976)	**Observational**<br>(Single-blind, cross-over trial, with placebo run-in)	CLN add-on in children with refractory epilepsy (absence seizures and myoclonic-atonic seizures), aged 6 months to 30 years (predominantly children)	N=20 (17 aged <18 years)	**NO**	Two patients withdrawn due to severe irritability, dysphoria and aggressiveness, and somnolence, behavioural disturbance and lack of efficacy, respectively
CLN	(Browne, 1976)	**Review**<br>(of clinical trials)			**NO**	Ten studies reported behavioural disturbance rates between 2-50% (e.g. irritable, aggressive, excitable, irrational, antisocial, temperamental, violent)<br>Some behavioural disturbance could be attributed to an exacerbation of the pre-existing condition<br>Dose-reduction was of benefit, but some patients discontinued
**Eslicarbazepine acetate (ESL)**						
ESL	(Almeida *et al.*, 2008)	Prospective open-label study	Pharmacokinetics of ESL monotherapy in children aged 2–17 with epilepsy	N=29	**NO**<br>Adverse event reporting	Aggressive behaviour in one child and "aggression aggravated" in one child
**Ethosuximide (ETX)**						
ETX	(Yamamoto *et al.*, 2001)	**Case-report**	Add-on ETX for refractory myoclonic epilepsy and severe psychomotor delay	N=1	**NO**<br>Healthcare professional/teacher/parental report	"Behavioural changes, more of the manic type" were reported (attributed to forced-normalisation)
ETX	(Chien, 2011)<br>(Chien, 2011)	**Case-report**	Absence epilepsy<br>Ethosuximide used as monotherapy	N=1	Physician, parental or self-report	Acute mania and psychotic symptoms with suicidal ideation
**Gabapentin (GBP)**						

AED	Reference	Study type/design	Treatment stage and seizure type	Total N	Outcome measure	Prevalence
GBP	(Lee *et al.*, 1996)	**Observational** (Retrospective chart review/parental follow-up)	Add-on GBP in children with focal seizures	Case series of 7 patients, from treated pool of N=55	**NO** Semi-structured parental interviews	7 of 55 children had behavioural changes (12.7%). Mostly intensification of existing behaviours, and some new behaviours emerged (oppositional behaviour, fighting, increased anger)
GBP	(Khurana *et al.*, 1996)	**Observational** (Retrospective chart review)	Add-on GBP in children aged 2–16 years with refractory partial epilepsy	N=32	**NO** Physician report	15/32 children showed behavioural adverse events (46.9%), these were mild in 11 patients, and required medical intervention in 4 patients (1 patient became withdrawn, and 3 became more hyperactive and aggressive, with violent outbursts and mood swings). GBP discontinued in three, and behaviour returned to baseline.
GBP	(Tallian *et al.*, 1996)	Case report	Two children who developed behavioural problems on low dose of GBP (monotherapy in 1 patient and add-on in the other)	N=2	**NO**	Two reports of aggression, both resolved with dose-reduction/discontinuity of GBP; one case became aggressive again once GBP was recommenced
GBP	(Wolf *et al.*, 1995)	Case report	Three patients with learning disability who developed severe behavioural problems with add-on GBP	**N=3**	**NO**	Anger outbursts, oppositional behaviour, hyperactivity
**Lacosamide (LCM)**						
LCM	(Gavatha *et al.*, 2011)	**Observational** (Prospective, open, uncontrolled)	Add-on LCM in children aged 3–17 with pharmacoresistant partial epilepsy	N=18	NO	Irritability: 2/18 (11.1%)
LCM	(Guilhoto *et al.*, 2011)	**Observational** (Retrospective chart review)	Add-on LCM in children and young adults (aged 8–21 years) with focal epilepsies of various pathologies and aetiologies Lacosamide used as add-on therapy	N=16	**NO** Parental/self-report of AEs	LCM withdrawn in 1 of the 16 children (patient aged 8.4 years) due to severe behavioural outbursts
LCM	(Heyman *et al.*, 2012)	**Observational** (Retrospective chart review)	LCM mono- or add-on therapy in children aged 1.5–16 years with refractory epilepsy	N=17	NO Parental/teacher-report	Restlessness in 2/17 patients (11.8%) but no other behavioural disturbance was reported Social/behavioural/motor improvement seen in 7/17 (41.2%)
LCM	(Kim *et al.*, 2014)	**Observational** (Retrospective chart review)	Add-on LCM in patients aged 1–18 years with refractory focal epilepsy of various aetiologies	N=21	NO Caregiver-report	Two patients (10%) discontinued treatment due to aggressive behaviour and depression
**Lamotrigine (LTG)**						
LTG	(Cardenas *et al.*, 2010)	**Observational** (Retrospective chart review and case series)	LTG add-on or monotherapy in various seizure types	N=9	**NO** Interviews, examinations, and routine medical records	Case series of 9 patients who had behaviour change (agitation and hyperactivity) with LTG. Five patients developed self-injurious and violent behaviours. One six-year-old boy developed an “extremely volatile mood and affect”, including visual and auditory hallucinations. All patients improved markedly following cessation/reduction of the lamotrigine.
LTG	(Piña-Garza *et al.*, 2008)	**Open-label** (Prospective, open-label extension of RCTs	Add-on LTG in multiple seizure types	N=204	**NO** AE reporting	Irritability attributed to lamotrigine in 5% of patients
**Levetiracetam (LEV)**						
LEV	(de la Loge *et al.*, 2010)	**RCT** (Randomised, double-blind, placebo-controlled, multicentre, safety study of behavioural and emotional functioning)	Adjunctive LEV vs. placebo in children and adolescents (aged 4–16 years) with partial onset seizures	N=98 (LEV N=64)	**YES CBCL, CHQ-PF50**	Significant worsening with LEV vs. PBO in the CBCL Total Problem Score (p=0.02), which includes the Aggressive Behaviour score (worse with LEV vs. PBO, p=0.013) and the Externalising Syndrome score (p=0.011). No significant difference with LEV vs. PBO in the CBCL Total Competence Score, but significant improvement with LEV vs. PBO in the Activities score (p=0.049) which is a component of the competence score. No significant difference between LEV and PBO on CHQ-PF50 scores.

AED	Reference	Study type/design	Treatment stage and seizure type	Total N	Outcome measure	Prevalence
LEV	(Schiemann-Delgado *et al.*, 2012)	**RCT open-label extension**<br>(Long-term extension of the RCT reported by de la Loge et al.)	Adjunctive LEV vs. placebo in children and adolescents (aged 4–16 years) with partial onset seizures, enrolled from previous RCT (N=80) or enrolled directly (N=23).	N=103	**YES**<br>**CBCL**<br>Also AE reporting	CBCL: scores overall, and also aggression scores, improved from baseline.<br>AE reports:<br>Aggression: 7.8%<br>Irritability: 7.8%<br>Abnormal behaviour: 3.9%<br>Withdrawal:<br>Anger: 1 patients<br>Abnormal behaviour: 1 patient
LEV	(Kanemura *et al.*, 2014)	**Observational**<br>**(**Prospective, non-comparative)study	Children with epilepsy and pervasive developmental disorder	N=12	**YES**<br>**Parental report of frequency of episodes panic/aggressive behaviours**	Improvement in behaviour with LEV.<br>Of the 8 patients whose seizures improved with LEV: Six (who all had frontal EEG paroxysms) showed a ≥50% reduction in panic/aggressive behaviour; two (with rolandic EEG paroxysms) had no change in behaviour.
LEV	(Halma *et al.*, 2014)	Meta-analysis of RCTs and observational studies	Children treated with LEV in mono- or add-on therapy, and with generalised and localisation-related epilepsies.	LEV N=727, from 13 studies	**NO**<br>Adverse event reporting	**Behavioural AEs in RCTs:**<br>LEV add-on therapy (N=165)<br>Hostility: 7.3%<br>Nervousness: 6.1%<br>Aggression: 4.9%<br>LEV mono- and add-on (N=203)<br>Hostility: 5.9%<br>Nervousness: 4.9%<br>Aggression: 3.9%<br>**Comparison:** Significant risk of behavioural side effects vs. placebo (2.18, 95% CI 1.42–3.37)<br>**Behavioural AEs in observational studies**<br>LEV add-on therapy (N=408)<br>Irritability: 4.7%<br>Hyperexcitability: 4.4%<br>Aggression: 2.7%<br>LEV monotherapy (N=116)<br>Behavioural problems: 19%<br>Irritability: 2.6%<br>LEV mono- and add-on (N=524)<br>Behavioural problems: 5%<br>Irritability: 4.2%<br>Hyperexcitability: 3.4%<br>Aggression: 2.1%<br>Agitation: 1.2%
LEV	(Glauser et al., 2006)*	**RCT**<br>(Double-blind, placebo-controlled Phase III trial)	Add-on LEV in patients aged 3–17 years with partial seizures ± secondary generalisation	N=198 (LEV N=101)	**NO**<br>Parental/self-report	Increased hostility, nervousness, emotional lability and agitation compared with placebo *(specific rates not reported)*
LEV	(Levisohn et al., 2009)*	**RCT**<br>(Randomised, double-blind, placebo-controlled Phase III trial)	Add-on LEV in patients with refractory epilepsy (predominantly partial-onset (98.4%))	N=98 (64 levetiracetam, 34 placebo)	**NO**	Increased aggression, altered mood, "abnormal behaviour" and "non-psychotic mood disorders" reported for LEV versus placebo [See de la Loge et al. for the behavioural results from this study, and Schiemann-Delgado et al. for the long-term results]
LEV	(Piña-Garza *et al.*, 2009)	**RCT**<br>Randomised, double-blind, placebo-controlled, multicentre Phase III trial	Add-on LEV in patients with refractory epilepsy (mixed seizure types and pathologies, predominantly partial-onset)	N=111 (58 levetiracetam, 53 placebo)	**NO**	Irritability: 11.7% (vs. 0% with PBO)
LEV	(Piña-Garza et al., 2010)*	Prospective, open-label, extension, multicentre	Add-on LEV in patients with partial-onset seizures (various pathologies and aetiologies)	N=152 (in intention-to-treat group)	**NO**<br>Parent-report of AEs	Irritability: 12.5%<br>Agitation: 3.9%<br>13 discontinued due to adverse events, two of which due to aggression.
LEV	(Fattore *et al.*, 2011)*	RCT<br>Randomised, double-blind, placebo-controlled, multicentre	LEV monotherapy in newly-diagnosed childhood/juvenile epilepsy	N=69 (LEV = 38)	**NO**<br>Parental/self-report of AEs	Adverse behavioural effects reported in two levetiracetam-treated children (irritability, and irritability and dysphoria, respectively)
LEV	(Callenbach *et al.*, 2008)	Prospective, open-label, multicentre	Add-on LEV in refractory epilepsy, various seizure types and syndromes	N=33	**NO**<br>Parental/self-report of AEs	Aggressive behaviour: 27.3%<br>Two children withdrew from the study as a result
LEV	(Wheless and Ng, 2002)	**Observational**<br>Open-label	Add-on LEV in children with refractory epilepsy	N=39	**NO**	Aggression: 12.8%
LEV	(Lagae *et al.*, 2005)*	**Observational**<br>Prospective, open, uncontrolled	Mono- and add-on LEV in children with various seizure types and aetiologies	N=77 (67 in an add-on study and 10 in a monotherapy study)	**NO**	Aggression: 5%<br>Improvement in behaviour: 22%
LEV	(Coppola *et al.*, 2004)	**Observational**<br>Prospective, open-label	Add-on LEV in children with refractory epilepsy (partial or generalised)	N=99	**NO**<br>Parental/self-report of AEs	Irritability: 7/99 (7%)<br>Aggression: 2/99 ((2%)
LEV	(Grosso, Franzoni, *et al.*, 2005)	**Observational**<br>Prospective, open, uncontrolled, multicentre	Add-on LEV for refractory epilepsy, various syndromes	N=110	**NO**<br>Parental report of AEs	**Irritability: 14%**
LEV	(Opp *et al.*, 2005)	**Observational**<br>Retrospective, open-label, multicentre	Add-on LEV in refractory, generalised/focal epilepsy	N=285	**NO**<br>Parental/self-report of AEs	**Aggression: 10.5%**
LEV	(Grosso *et al.*, 2007)*	**Observational**<br>Retrospective, uncontrolled, multicentre	Add-on LEV in refractory focal, generalised, and partial seizures	N=81	**NO**<br>Physician-report of AEs	Mild and transient irritability reported in 28%

**AED**	Reference	**Study type/design**	**Treatment stage and seizure type**	**Total N**	**Outcome measure**	**Prevalence**
LEV	(Khurana *et al.*, 1996)*	**Observational** Retrospective chart review	LEV monotherapy in partial (N=14) and generalised (N=4) epilepsy	N=18	**NO** Physician-report of AEs	Irritability: n=1 Significant behavioural changes (requiring discontinuation of levetiracetam) in two others
LEV	(Peake *et al.*, 2007)	**Observational** Retrospective chart review	LEV mono (7%)- or add-on therapy in refractory epilepsy; various aetiologies	N=200	**NO** Report of AEs	Of 17 children with pre-existing behavioural problems, behavioural problems were reported in 7 patients on follow-up Among the patients with no pre-existing behavioural problems, problems were reported in 14% on follow-up.
LEV	(Perry and Benatar, 2007)	**Observational** Retrospective chart review	LEV mono- (39%) or add-on therapy in children aged <4 years with various seizure types	N=122	**NO** Physician-report	Behavioural disturbance and/or irritability: 22% 8.2% discontinued
LEV	(Lee *et al.*, 2010)	**Observational** Open-label	Add-on LEV in childhood intractable epilepsy	N=130	**NO** Chart review	Irritability: 5% Aggression: 2% Two discontinued because of increased aggression
LEV	(Obeid and Pong, 2010)	**Observational** (Retrospective chart review)	LEV add-on (n=31) or monotherapy (n=1) in patients with various seizure types (50% partial) and aetiologies	N=32	**NO** Physician-report	Behavioural AEs: 12% (irritability n=3) Treatment discontinued in one patient due to increase irritability (not ameliorated by pyridoxine)
LEV	(Specchio *et al.*, 2010)	**Observational** (Prospective, open-label)	Add-on LEV in patients (7.5-19 years) with drug-resistant Rett syndrome	N=8	**NO** Parental/self-report	Two patients had transient irritability, which resolved as the treatment continued
LEV	(Feng *et al.*, 2015)	**Observational** (Prospective, open-label)	LEV add-on or monotherapy in various seizure types and aetiologies	N=124	**NO** Parental/self-report	Irritability was reported in 38.7%, and "abnormal behaviour" was reported in 13.3% (in the 75 included in the efficacy study)
**Oxcarbazepine (OXC)**						
OXC	(Northam *et al.*, 2005)	**Observational** Prospective, open-label	Mono- or add-on OXC therapy in young children (2–45 months) with partial seizures	N=24	**NO** AE reporting	Irritability: 21% during the treatment phase (30 days) and 35% in the extension phase (six months) One patient withdrew due to irritability (associated with fatigue and ataxia
**Perampanel (PMP)**						
PMP	(Lagae *et al.*, 2014)	**RCT** Phase II, randomised, placebo-controlled safety study	Add-on PMP in adolescents (12–17 years) with refractory partial seizures	N=133	**YES CBCL**	No difference in CBCL scores/change from baseline between PMP and PBO. AE reports: hostility/aggression reported in 17.6% of PMP patients (vs. 4.2% PBO)
PMP	(Rosenfeld *et al.*, 2015)	**RCT** Pooled results from multinational, double-blind, phase-III core studies, and extension study	Add-on PMP in adolescents (12–17 years) with drug-resistant partial seizures	**N=143**	**NO** Adverse event reporting	Aggression: 8/143 (8.2%) vs. 0 with PBO, and 1 discontinuation. Two SAEs of aggression. Total events during 1-year follow-up: -Aggression: n=22 (18.2%) -Abnormal behaviour: n=5 (4.1%) Dose interruption required in 5 patients and discontinuation in 3 patients due to aggression (total 6.6% adjustment/withdrawal)
PMP	(Biro *et al.*, 2013)	**Observational** Retrospective chart review	PMP monotherapy (n=6) or add-on (n=52), in children aged 2–17 years with various refractory epilepsies, multiple seizure types and aetiologies	N=58	**NO** Physician-report	Aggression: 8/58 (13.8%)
PMP	(Philip *et al.*, 2014)	**Observational** (Retrospective chart review)	PMP in children aged 4–19 years with refractory epilepsies of multiple aetiologies	N=18	**NO**	Behavioural change: 1/18 (5.6%) reported in one patient
**Phenobarbital (PHB)**						
PHB	(Willis *et al.*, 1997)	Open comparative study on and off the medication with matched control group	Multiple seizure types and aetiologies	Total of 11 children, eight on phenobarbital and three on mephobarbital (plus 13 age- and IQ-matched controls)	**YES Achenbach Behaviour Rating Scale**; parental report	Treatment with phenobarbital and mephobarbital The parents of six patients (five on phenobarbital) reported behavioural changes (irritability, oppositionality and overactivity) Four of the six had only mild changes and did not discontinue the drug
PHB	(Domizio *et al.*, 1993)	**Observational** Open study	PHB add-on therapy in children with febrile convulsions and "different types of epilepsy"	N=300 (N=197 PHB, N=103 other AEDs)	**NO**	Statistically significantly higher rate of behavioural disturbance (76.1%) in the PHB group than in the other AED group (31%). Hyperactivity most common behavioural disturbance

AED	Reference	Study type/design	Treatment stage and seizure type	Total N	Outcome measure	Prevalence
PHB	(de Silva *et al.*, 1996)	Randomised (multiple AEDs, including PHB)	Generalised tonic-clonic or partial (± secondary generalisation)<br>Drugs (including PHB) were initiated as monotherapy	N=167 (N=10 PHB)	Not stated	PHB-arm of the study was discontinued after six out of ten children had unacceptable, primarily behavioural, adverse effects
PHB	(Pal *et al.*, 1998)	Prospective, randomised	PHB monotherapy for generalised and partial-onset seizures	N=94 (N=47 PHB)	**YES**<br>Bengali-adaptation of the Conners scale (parent); behaviour screening questionnaire (<6 years)	No behavioural effects found
**Phenytoin**						
PHT	(Pal *et al.*, 1998)	Prospective, randomised	PHT monotherapy for generalised and partial seizures	N=94 (N=47 PHT)	**YES**<br>Bengali adaptation of the Conners (parent); behaviour screening questionnaire (<6 years)	No behavioural effects found
PHT	(Krishnamoorthy *et al.*, 1983)	**Case-reports**	Seizure types, syndrome and aetiologies not discussed<br>Commencement of PHT was in response to acute presentation, and was used as monotherapy in two cases and with PHB in one	N=3	**NO**<br>Physician/parent-report	Reports of phenytoin-induced choreoathetosis, which was associated with agitation/restlessness
**Retigabine (RTG)**						
RTG	(Groening *et al.*, 2012)	**Not stated**	RTG add-on in patients aged 10–19 years with pharmacoresistant epilepsies	N=17 (data available for 12)	**NO**<br>Parental/self-report	25% reported agitation and personality changes
**Rufinamide (RUF)**						
RUF	(Coppola *et al.*, 2014)	Consensus paper	N/A – no new data presented	Data from 481 patients (1-50 years) summarised	Dependent on individual studies	No reports of adverse effects involving aggression or related behaviours
RUF	(Coppola *et al.*, 2010)	**Observational**<br>Prospective, open-label, add-on, multicentre	Add-on RUF in patients aged 4–34 years with Lennox-Gastaut syndrome	N=43	**NO**<br>Parental report	Irritability/aggressiveness was reported in 6.9% of patients
RUF	(Coppola *et al.*, 2011)	**Observational**<br>Prospective, open-label, add-on, multicentre	Add-on RUF in patients aged 4–34 with non-LGS encephalopathies	N=38	**NO**<br>Parental report	Irritability/aggressiveness was reported in 5.3% of patients
RUF	(Cusmai *et al.*, 2014)	**Observational**<br>Prospective, open-label, add-on	Add-on RUF in patients with refractory epilepsy (multiple seizure types and pathologies/aetiologies	**N=69**	**NO**<br>Parental/self-report	Irritability: 15.9%
RUF	(Lee *et al.*, 2013)	**Observational**<br>Retrospective chart review	Add-on RUF in patients aged 4–22 years with LGS	N=23	**NO**<br>Parental report	Aggressive behaviour reported in two patients
**Valproic acid / sodium valproate (VPA)**						
VPA	(Ronen *et al.*, 2000)	Randomised, double-blind, single-crossover	VPA monotherapy in patients with abnormal EEG findings but no clinical seizures	N=8	**YES**<br>**Child Behaviour Checklist (CBCL)**	No reports of aggression specifically but children more distractible and parents reported higher (worse) internalising scores on the CBCL
VPA	(Egger and Brett, 1981)	**Observational**<br>Retrospective	VPA monotherapy (n=85) or add-on (n=15) in various seizure types; pathologies and aetiologies	N=100	**NO**<br>Parental report	Aggressive behaviour: 4%
**Stiripentol (STR)**						
STR	(Inoue *et al.*, 2009)	**Observational**<br>Multicentre chart review	STR add-on in patients aged 1–46 years with Dravet syndrome	N=23	**NO**<br>Parental report	Hyperactivity/irritability: 26% during the first four weeks following titration<br>These effects resolved with continued treatment/dose reduction<br>One patient withdrew due to early irritability
**Topiramate (TPM)**						
TPM	(Reith *et al.*, 2003)	**Observational**<br>Retrospective cohort chart review	TPM add-on (monotherapy n=3) in multiple seizure types and pathologies/aetiologies	N=159 (data available for 127)	**NO**<br>Physician report	Aggression/psychosis was reported as a treatment-limiting adverse effect in 7.8% of patients
TPM	(Grosso, Galimberti, *et al.*, 2005)	**Observational**<br>Prospective, open	TPM add-on in patients with refractory seizures of multiple aetiologies	N=59	**NO**<br>Parental report	37% reported adverse events, of which irritability was one of the most common; no further data given
TPM	(Endoh *et al.*, 2012)	**Observational**<br>Apparently prospective	TPM add-on in intractable childhood generalised epilepsy with epileptic spasms (Ohtahara, West, Lennox-Gastaut syndromes or related disorders)	N=33	**NO**	Irritability: 15.2%
**Vigabatrin (VGT)**						

**AED**	Reference	**Study type/design**	**Treatment stage and seizure type**	**Total N**	**Outcome measure**	**Prevalence**
VGT	(Sheth *et al.*, 1996)	**Observational** (Prospective, open label)	Add-on VGT for refractory generalised and partial seizures, multiple pathologies/aetiologies, in patients aged 1–22 years (mean 12.6 years)	N=31	**YES** Parental and teacher report of behaviour (alertness, activity level, school grades, and general)	Negative behaviour change in 6 children (by parent report, 5 by school report) Positive behaviour change in 1 (by parent report; 2 by school report). VGT discontinued in 1 child due to severe aggressive agitation, and behaviour returned to normal
VGT	(Gobbi *et al.*, 1999)	**Observational** (Prospective, open, non-randomised, comparative pilot study)	VGT or CBZ monotherapy in children with newly-diagnosed partial epilepsies Mixed pathologies/aetiologies VGB used as monotherapy	N=80 (VGT N=40; CBZ N=40)	**NO** Physician, parent or self-report	4/37 (10.8%) in the vigabatrin group reported mild irritability at the end of the study (vs none in the CBZ group)
VGT	(Luna *et al.*, 1989)	**Observational** (Single-blind, placebo run-in study)	Add-on VGT in children aged 1–19 years, with various generalised and localisation-related epilepsies	N=61	**NO** Adverse event reporting	Agitation, aggression, hyperactivitiy: 18/61 (29.5%), vs 1/61 (1.6%) during placebo phase. 3/18 discontinued VGT, symptoms resolved with no change or dose reduction in the remainder
VGT	(Livingston *et al.*, 1989)	**Observational** (Prospective, open, uncontrolled study)	Infants and children aged 2–12 years with refractory epilepsy (partial, generalised, West Syndrome, LGS)	N=135	**NO** Adverse event reporting	Excitation/agitation: 8.8% (severe in 2/9)
VGT	(Raucci *et al.*, 1994)	**Observational**	VGT add-on or monotherapy in children with severe epilepsies of various types	N=61 (12 monotherapy)	**NO**	Discontinued in six patients due to adverse effects, including irritability, somnolence, weight gain and cutaneous rash (Italian paper – further information not available in English)
VGT	(Gherpelli *et al.*, 1997)	**Observational** (Prospective, open-label	Add-on VGT in children aged 6 months–18 years with severe refractory epilepsy of various types (excluding West syndrome)	N=47	**NO** Adverse event reporting	Hyperactivity: N=5 (10.6%) Irritability: N=5 (10.6%), leading to withdrawal in 1 patient.
VGT	(Weber *et al.*, 2012)	**Case-report**	Adolescent girl receiving add-on VGT for refractory symptomatic epilepsy	N=1	Physician report	Psychosis, attributed to forced normalisation.
VGT	(Cánovas Martínez *et al.*, 1995)	**Case-report**	Seven-year-old boy with refractory epilepsy receiving VGT with rapid dose escalation	N=1	Physician report	Psychosis, attributed to rapid induction, which resolved after withdrawal of treatment (and failed to return following subsequent slow titration of VGT)
VGT	(Chiaretti *et al.*, 1994)	**Case-report**	Child treated with VGT	N=1	Physician report	Psychosis
VGT	(Appleton *et al.*, 1999)	**RCT** (Randomised, placebo-controlled study)	First-line VGT in infantile spasms	N=40 (VGT N=20)	**NO** Adverse event reporting	Irritability and marked behaviour change in 1 VGT-treated patient (5%), placebo rate not reported.
VGT	(Lux *et al.*, 2004)(UKISS)	**Interventional** (Open, randomised, parallel-group, multicentre trial)	Infants with infantile spasms of various aetiologies, randomised to first-line VGT or hormonal treatments (prednisolone or tetracosactide)	N=107 (VGT N=52; hormonal N=55)	**NO** Parental-report of adverse events	Irritability: 3.8% with VGT ((vs 34.5% with hormones) Neuropsychiatric (incl sleep disturbance): 7.7% with VGT (vs. 1.8% with hormones)
VGT	(Vigevano and Cilio, 1997)	**Interventional** (Prospective, randomised, response-mediated cross-over study)	VGT vs ACTH for infantile spasms	N=65 (VGT N=23)	**NO** Adverse event reporting	Irritability: N=1 (4%) vs 7 (37%) with ACTH
VGT	(Fejerman *et al.*, 2000)	**Observational** (Prospective case review)	Infants who received VGT for infantile spasms	N=116	**NO** Adverse event reporting	Irritability: N=15 (12.9%), no discontinuations
VGT	(Kankirawatana *et al.*, 2002)	**Observational** (case review)	Infants who received VGT for infantile spasms	N=36	**NO** Adverse event reporting	Irritability: N=3 (8.3%)
VGT	(Tay *et al.*, 2001)	**Observational** (Retrospective case review)	Asian infants who received VGT monotherapy or add-on therapy for infantile spasms	N=18	**NO** Adverse event reporting	Irritability: N=2 (11.1%)
**Zonisamide (ZNS)**						
ZNS	(Eun *et al.*, 2011)	**Interventional** (randomised, open-label, multicentre study)	ZNS monotherapy in children aged 2–16 years with newly diagnosed epilepsy of various types	N=90 Behavioural data available for N=63 (27 low-dose, 36 high-dose)	**YES** **Korean CBCL** and AE reporting	CBCL showed significant improvement in aggression from baseline in the low-dose group, and small non-significant improvement in the high-dose group No aggression-related AEs reported.
ZNS	(Cross *et al.*, 2014)	**Pooled analysis** Pooled analysis of 17 ZNS trials (four of which were randomised and double-blind)	Most studies were add-on treatment of partial seizures, some used ZNS monotherapy.	N=398 (391 treated with zonisamide)	**NO** Parental/self-report of AEs	Irritability: 5.8%, and not listed as a common reason for discontinuation Irritability more common in younger patients (6-11 years) than in adolescents (12-16 years) (7.5% vs. <5%)
ZNS	(Guerrini *et al.*, 2013)	**RCT** (Randomised, double-blind, placebo-controlled, multicentre phase III trial)	ZNS add-on treatment of partial seizures in children aged 6–17 years	N=207 (100 placebo)	**NO** Parental/self-report of AEs	Incidence of aggression-related AEs not given (<3%); 1 placebo patient discontinued due to aggression

AED	Reference	Study type/design	Treatment stage and seizure type	Total N	Outcome measure	Prevalence
ZNS	(Guerrini *et al.*, 2014)	**RCT extension** (Extension of the study above; blinded titration followed by open-label ZNS)	ZNS add-on treatment of partial seizures in children aged 6–17 years	N=144 (72 previously taking placebo)	**YES CBCL, School Performance Questionnaire** Parental/self-report of AEs also collected	No significant change in CBCL or school performance from baseline. Aggression reported as AE in 2 patients who switched to ZNS from placebo (2.8%)
ZNS	(Hirai *et al.*, 2002)	**Observational** (Prospective study with case report of behavioural AEs)	Children with idiopathic epilepsy treated with zonisamide monotherapy	N=27 (two case-reports)	**NO Parental/self-report**	Two of 27 patients developed behavioural changes (7.4%) during effective seizure control with ZNS. One patient developed OCD 3 years after starting ZNS, and one patient developed selective mutism, violent behaviour and poor concentration, 4 years after starting ZNS.
ZNS	(Coppola *et al.*, 2009)	**Observational** (Prospective, open-label)	Add-on ZNS in children and adults (3–34 years) with partial or generalised refractory epilepsies	**N=82**	**NO** Parental/self-report	Irritability reported in 11%; resolved in most by dose reduction
*Studies that were selected for inclusion in the Halma et al. systematic review and meta-analysis of levetiracetam adverse events in children. CBCL: Achenbach Child Behaviour Checklist; CHQ-PF50: Child Health Questionnaire – Parent Form 50;						

D. References

Almeida L, Minciu I, Nunes T, Butoianu N, Falcão A, Magureanu S-A, and Soares-da-Silva P (2008) Pharmacokinetics, efficacy, and tolerability of eslicarbazepine acetate in children and adolescents with epilepsy. *J Clin Pharmacol* **48**:966–977.

Appleton RE, Peters AC, Mumford JP, and Shaw DE (1999) Randomised, placebo-controlled study of vigabatrin as first-line treatment of infantile spasms. *Epilepsia* **40**:1627–1633.

Armour DJ, Fidler C, Wright EC, and Balarajan S (1992) Vigabatrin in adults with poorly-controlled epilepsy and learning disabilities. *Seizure* **1**:157–162.

Bawden HN, Camfield CS, Camfield PR, Cunningham C, Darwish H, Dooley JM, Gordon K, Ronen G, Stewart J, and van Mastrigt R (1999) The cognitive and behavioural effects of clobazam and standard monotherapy are comparable. Canadian Study Group for Childhood Epilepsy. *Epilepsy Res* **33**:133–143.

Belcastro V, Costa C, Galletti F, Autuori A, Pierguidi L, Pisani F, Calabresi P, and Parnetti L (2008) Levetiracetam in newly diagnosed late-onset post-stroke seizures: a prospective observational study. *Epilepsy Res* **82**:223–226.

Ben-Menachem E, and Gilland E (2003) Efficacy and tolerability of levetiracetam during 1-year follow-up in patients with refractory epilepsy. *Seizure* **12**:131–135.

Beran RG, and Gibson RJ (1998) Aggressive behaviour in intellectually challenged patients with epilepsy treated with lamotrigine. *Epilepsia* **39**:280–282.

Biro A, Muller A, Stephani U, Tarallo T, and Schlachter K (2013) Effectiveness and tolerability of perampanel after 3 months of treatment in children and adolescents with refractory epilepsy first european experience.

Biton V, Berkovic SF, Abou-Khalil B, Sperling MR, Johnson ME, and Lu S (2014) Brivaracetam as adjunctive treatment for uncontrolled partial epilepsy in adults: a phase III randomized, double-blind, placebo-controlled trial. *Epilepsia* **55**:57–66.

Bootsma HPR, Coolen F, Aldenkamp AP, Arends J, Diepman L, Hulsman J, Lambrechts D, Leenen L, Majoie M, Schellekens A, and de Krom M (2004) Topiramate in clinical practice: long-term experience in patients with refractory epilepsy referred to a tertiary epilepsy center. *Epilepsy Behav* **5**:380–387.

Bootsma HPR, Ricker L, Diepman L, Gehring J, Hulsman J, Lambrechts D, Leenen L, Majoie M, Schellekens A, de Krom M, and Aldenkamp AP (2007) Levetiracetam in clinical practice: long-term experience in patients with refractory epilepsy referred to a tertiary epilepsy center. *Epilepsy Behav* **10**:296–303.

Browne TR (1976) Clonazepam. A review of a new anticonvulsant drug. *Arch Neurol* **33**:326–332.

Callenbach PMC, Arts WFM, ten Houten R, Augustijn P, Gunning WB, Peeters EAJ, Weber AM, Stroink H, Geerts Y, Geerts AT, and Brouwer OF (2008) Add-on levetiracetam in children and adolescents with refractory epilepsy: results of an open-label multi-centre study. *Eur J Paediatr Neurol EJPN Off J Eur Paediatr Neurol Soc* **12**:321–327.

Cánovas Martínez A, Ordovás Baines JP, Beltrán Marqués M, Escrivá Aparisi A, and Delgado Cordón F (1995) Vigabatrin-associated reversible acute psychosis in a child. *Ann Pharmacother* **29**:1115–1117.

Cardenas JF, Rho JM, and Ng Y (2010) Reversible lamotrigine-induced neurobehavioral disturbances in children with epilepsy. *J Child Neurol* **25**:182–187.

Carmichael K, Pulman J, Lakhan SE, Parikh P, and Marson AG (2013) Zonisamide add-on for drug-resistant partial epilepsy. *Cochrane Database Syst Rev* **12**:CD001416.

Chiaretti A, Castorina M, Tortorolo L, Piastra M, and Polidori G (1994) [Acute psychosis and vigabatrin in childhood]. *Pediatr Medica E Chir Med Surg Pediatr* **16**:489–490.

Chien J (2011) Ethosuximide-induced mania in a 10-year-old boy. *Epilepsy Behav* **21**:483–485.

Chung S, Wang N, and Hank N (2007) Comparative retention rates and long-term tolerability of new antiepileptic drugs. *Seizure* **16**:296–304.

Ciesielski A-S, Samson S, and Steinhoff BJ (2006) Neuropsychological and psychiatric impact of add-on titration of pregabalin versus levetiracetam: a comparative short-term study. *Epilepsy Behav* **9**:424–431.

Coppola G, Besag F, Cusmai R, Dulac O, Kluger G, Moavero R, Nabbout R, Nikanorova M, Pisani F, Verrotti A, von Stülpnagel C, and Curatolo P (2014) Current role of rufinamide in the treatment of childhood epilepsy: literature review and treatment guidelines. *Eur J Paediatr Neurol* **18**:685–690.

Coppola G, Grosso S, Franzoni E, Veggiotti P, Zamponi N, Parisi P, Spalice A, Habetswallner F, Fels A, Capovilla G, Verrotti A, Mangano S, Balestri A, Curatolo P, and Pascotto A (2010) Rufinamide in children and adults with Lennox-Gastaut syndrome: first Italian multicenter experience. *Seizure* **19**:587–591.

Coppola G, Grosso S, Franzoni E, Veggiotti P, Zamponi N, Parisi P, Spalice A, Habetswallner F, Fels A, Verrotti A, D'Aniello A, Mangano S, Balestri A, Curatolo P, and Pascotto A (2011) Rufinamide in refractory childhood epileptic encephalopathies other than Lennox-Gastaut syndrome. *Eur J Neurol* **18**:246–251.

Coppola G, Grosso S, Verrotti A, Parisi P, Luchetti A, Franzoni E, Mangano S, Pelliccia A, Operto FF, Iannetti P, Curatolo P, Balestri P, and Pascotto A (2009) Zonisamide in children and young adults with refractory epilepsy: an open label, multicenter Italian study. *Epilepsy Res* **83**:112–116.

Coppola G, Mangano S, Tortorella G, Pelliccia A, Fels A, Romano A, Nardello R, Habetswallner F, Licciardi F, Operto FF, and Pascotto A (2004) Levetiracetam during 1-year follow-up in children, adolescents, and young adults with refractory epilepsy. *Epilepsy Res* **59**:35–42.

Coyle H, Clough P, Cooper P, and Mohanraj R (2014) Clinical experience with perampanel: focus on psychiatric adverse effects. *Epilepsy Behav* **41**:193–196.

Cramer JA, De Rue K, Devinsky O, Edrich P, and Trimble MR (2003) A systematic review of the behavioral effects of levetiracetam in adults with epilepsy, cognitive disorders, or an anxiety disorder during clinical trials. *Epilepsy Behav* **4**:124–132.

Cross JH, Auvin S, Patten A, and Giorgi L (2014) Safety and tolerability of zonisamide in paediatric patients with epilepsy. *Eur J Paediatr Neurol* **18**:747–758.

Cusmai R, Verrotti A, Moavero R, Curatolo P, Battaglia D, Matricardi S, Spalice A, Vigevano F, Pruna D, Parisi P, D'Aniello A, Di Gennaro G, and Coppola G (2014) Rufinamide for the treatment of refractory epilepsy secondary to neuronal migration disorders. *Epilepsy Res* **108**:542–546.

de la Loge C, Hunter SJ, Schiemann J, and Yang H (2010) Assessment of behavioral and emotional functioning using standardized instruments in children and adolescents with partial-onset seizures treated with adjunctive levetiracetam in a randomized, placebo-controlled trial. *Epilepsy Behav* **18**:291–298.

de Silva M, MacArdle B, McGowan M, Hughes E, Stewart J, Neville BG, Johnson AL, and Reynolds EH (1996) Randomised comparative monotherapy trial of phenobarbitone, phenytoin, carbamazepine, or sodium valproate for newly diagnosed childhood epilepsy. *Lancet* **347**:709–713.

Dinkelacker V, Dietl T, Widman G, Lengler U, and Elger CE (2003) Aggressive behavior of epilepsy patients in the course of levetiracetam add-on therapy: report of 33 mild to severe cases. *Epilepsy Behav EB* **4**:537–547.

Dolton E, and Choudry A (2014) Perampanel and challenging behaviour in intellectual disability and epilepsy: a management dilemma. *Case Rep Psychiatry* **2014**:409209.

Domizio S, Verrotti A, Ramenghi LA, Sabatino G, and Morgese G (1993) Anti-epileptic therapy and behaviour disturbances in children. *Childs Nerv Syst* **9**:272–274.

D'Souza J, Johnson M, and Borghs S (2012) Meta-analysis of non-psychotic behavioural treatment-emergent adverse events in brivaracetam and levetiracetam development programmes (abstract P402). *Epilepsia* **53 (Suppl. 5)**:118.

Edwards VE (1974) Side effects of clonazepam therapy. *Proc Aust Assoc Neurol* **11**:199–202.

Egger J, and Brett EM (1981) Effects of sodium valproate in 100 children with special reference to weight. *Br Med J* **283**:577–581.

Eisenschenk S, Krop H, and Devinsky O (2014) Homicide during postictal psychosis. *Epilepsy Behav Case Rep* **2**:118–120.

Endoh F, Kobayashi K, Hayashi Y, Shibata T, Yoshinaga H, and Ohtsuka Y (2012) Efficacy of topiramate for intractable childhood generalized epilepsy with epileptic spasms: with special reference to electroencephalographic changes. *Seizure* **21**:522–528.

Ettinger AB, Jandorf L, Berdia A, Andriola MR, Krupp LB, and Weisbrot DM (1996) Felbamate-induced headache. *Epilepsia* **37**:503–505.

Ettinger AB, LoPresti A, Yang H, Williams B, Zhou S, Fain R, and Laurenza A (2015) Psychiatric and behavioral adverse events in randomized clinical studies of the noncompetitive AMPA receptor antagonist perampanel. *Epilepsia* **56**:1252–1263.

Ettinger AB, Weisbrot DM, Saracco J, Dhoon A, Kanner A, and Devinsky O (1998) Positive and negative psychotropic effects of lamotrigine in patients with epilepsy and mental retardation. *Epilepsia* **39**:874–877.

Ettinger AB, Yang H, Williams B, Zhou S, Fain R, and Laurenza A (2014) Review of psychiatric and behavioural events in perampanel clinical studies (abstract 1.146). *Epilepsy Curr* **14 (S1)**:67.

Eun S-H, Kim HD, Eun B-L, Lee IK, Chung HJ, Kim JS, Kang H-C, Lee Y-M, Suh ES, Kim DW, Eom S, Lee JS, and Moon HK (2011) Comparative trial of low- and high-dose zonisamide as monotherapy for childhood epilepsy. *Seizure* **20**:558–563.

Fain R, Chung S, Yang H, Xing D, Williams B, and Laurenza A (2015) Aggression adverse events with concomitant levetiracetam use in perampanel phase III partial-onset seizure clinical studies (Abstract 2.288). *Epilepsy Curr* **15 (S1)**:321.

Fattore C, Boniver C, Capovilla G, Cerminara C, Citterio A, Coppola G, Costa P, Darra F, Vecchi M, and Perucca E (2011) A multicenter, randomized, placebo-controlled trial of levetiracetam in children and adolescents with newly diagnosed absence epilepsy. *Epilepsia* **52**:802–809.

Faught E, Morris G, Jacobson M, French J, Harden C, Montouris G, and Rosenfeld W (1999) Adding lamotrigine to valproate: incidence of rash and other adverse effects. Postmarketing Antiepileptic Drug Survey (PADS) Group. *Epilepsia* **40**:1135–1140.

Fejerman N, Cersósimo R, Caraballo R, Grippo J, Corral S, Martino RH, Martino G, Aldao M, Caccia P, Retamero M, Macat MC, Di Blasi MA, and Adi J (2000) Vigabatrin as a first-choice drug in the treatment of West syndrome. *J Child Neurol* **15**:161–165.

Feng X, Chen Y, Liu L, and Xiao N (2015) Retention rates of levetiraceram in Chinese children and adolescents with epilepsy. *Eur J Paediatr Neurol EJPN Off J Eur Paediatr Neurol Soc* **19**:143–148.

Flynn C, and Delanty N (2014) Seizure response to perampanel in a severe refractory group of epilepsy patients (abstract p343). *Epilepsia* **55 (Suppl. 2)**:113.

French JA, Costantini C, Brodsky A, von Rosenstiel P, and N01193 Study Group (2010) Adjunctive brivaracetam for refractory partial-onset seizures: a randomized, controlled trial. *Neurology* **75**:519–525.

French JA, Krauss GL, Biton V, Squillacote D, Yang H, Laurenza A, Kumar D, and Rogawski MA (2012) Adjunctive perampanel for refractory partial-onset seizures: randomized phase III study 304. *Neurology* **79**:589–596.

French JA, Krauss GL, Steinhoff BJ, Squillacote D, Yang H, Kumar D, and Laurenza A (2013) Evaluation of adjunctive perampanel in patients with refractory partial-onset seizures: results of randomized global phase III study 305. *Epilepsia* **54**:117–125.

French JA, Krauss GL, Wechsler RT, Wang X-F, DiVentura B, Brandt C, Trinka E, O'Brien TJ, Laurenza A, Patten A, and Bibbiani F (2015) Perampanel for tonic-clonic seizures in idiopathic generalized epilepsy A randomized trial. *Neurology* **85**:950–957.

French J, Edrich P, and Cramer JA (2001) A systematic review of the safety profile of levetiracetam: a new antiepileptic drug. *Epilepsy Res* **47**:77–90.

Frey LC, Strom LA, Shrestha A, and Spitz MC (2009) End-of-dose emergent psychopathology in ambulatory patients with epilepsy on stable-dose lamotrigine monotherapy: a case series of six patients. *Epilepsy Behav* **15**:521–523.

Friedman DL, Kastner T, Plummer AT, Ruiz MQ, and Henning D (1992) Adverse behavioral effects in individuals with mental retardation and mood disorders treated with carbamazepine. *Am J Ment Retard* **96**:541–546.

Gavatha M, Ioannou I, and Papavasiliou AS (2011) Efficacy and tolerability of oral lacosamide as adjunctive therapy in pediatric patients with pharmacoresistant focal epilepsy. *Epilepsy Behav* **20**:691–693.

Gherpelli JL, Guerreiro MM, da Costa JC, Rotta NT, Manreza ML, Reed UC, Diament A, Silva EA, Guerreiro CA, Nunes ML, Palmini A, Vega-Gutiérrez L, Vizioli J, Pedroso F, and Chisté MA (1997) Vigabatrin in refractory childhood epilepsy. The Brazilian Multicenter Study. *Epilepsy Res* **29**:1–6.

Gidal BE, Ferry J, Majid O, and Hussein Z (2013) Concentration-effect relationships with perampanel in patients with pharmacoresistant partial-onset seizures. *Epilepsia* **54**:1490–1497.

Glauser TA, Ayala R, Elterman RD, Mitchell WG, Van Orman CB, Gauer LJ, Lu Z, and N159 Study Group (2006) Double-blind placebo-controlled trial of adjunctive levetiracetam in pediatric partial seizures. *Neurology* **66**:1654–1660.

Gobbi G, Pini A, Bertani G, Menegati E, Tiberti A, Valseriati D, Besana D, Rasmini P, Guerrini R, Belmonte A, Veggiotti P, Resi C, Lanzi G, Capovilla G, Galeone D, and Milani S (1999) Prospective study of first-line vigabatrin monotherapy in childhood partial epilepsies. *Epilepsy Res* **35**:29–37.

Goulding P, Wright E, Geldard J, and Maguire M (2014) A service evaluation of perampanel (Fycompa) at leeds general infirmary (abstract p335). *Epilepsia* **55 (Suppl. 2)**:111.

Greshake B, and Straub H-B (2014) Tolerability and effectiveness of perampanel in treatment of patients with pharmacoresistant epilepsy (abstract p124). *Epilepsia* **55 (Suppl. 2)**:46.

Groening K, Dreiwes C, Stephani U, and Boor R (2012) Retigabine in children and adolescents with pharmacoresistant epilepsies - documentation with the electronic treatment diary Epi-Vista(R). *Epilepsia* **53**:119–120.

Grosso S, Cordelli DM, Franzoni E, Coppola G, Capovilla G, Zamponi N, Verrotti A, Morgese G, and Balestri P (2007) Efficacy and safety of levetiracetam in infants and young children with refractory epilepsy. *Seizure* **16**:345–350.

Grosso S, Franzoni E, Coppola G, Iannetti P, Verrotti A, Cordelli DM, Marchiani V, Pascotto A, Spalice A, Acampora B, Morgese G, and Balestri P (2005) Efficacy and safety of levetiracetam: an add-on trial in children with refractory epilepsy. *Seizure* **14**:248–253.

Grosso S, Galimberti D, Farnetani MA, Cioni M, Mostardini R, Vivarelli R, Di Bartolo RM, Bernardoni E, Berardi R, Morgese G, and Balestri P (2005) Efficacy and safety of topiramate in infants according to epilepsy syndromes. *Seizure* **14**:183–189.

Guerrini R, Rosati A, Bradshaw K, and Giorgi L (2014) Adjunctive zonisamide therapy in the long-term treatment of children with partial epilepsy: results of an open-label extension study of a phase III, randomized, double-blind, placebo-controlled trial. *Epilepsia* **55**:568–578.

Guerrini R, Rosati A, Segieth J, Pellacani S, Bradshaw K, and Giorgi L (2013) A randomized phase III trial of adjunctive zonisamide in pediatric patients with partial epilepsy. *Epilepsia* **54**:1473–1480.

Guilhoto LMFF, Loddenkemper T, Gooty VD, Rotenberg A, Takeoka M, Duffy FH, Coulter D, Urion D, Bourgeois BF, and Kothare SV (2011) Experience with lacosamide in a series of children with drug-resistant focal epilepsy. *Pediatr Neurol* **44**:414–419.

Halma E, de Louw AJA, Klinkenberg S, Aldenkamp AP, IJff DM, and Majoie M (2014) Behavioral side-effects of levetiracetam in children with epilepsy: a systematic review. *Seizure* **23**:685–691.

Helmstaedter C, Fritz NE, Kockelmann E, Kosanetzky N, and Elger CE (2008) Positive and negative psychotropic effects of levetiracetam. *Epilepsy Behav* **13**:535–541.

Helmstaedter C, Mihov Y, Toliat MR, Thiele H, Nuernberg P, Schoch S, Surges R, Elger CE, Kunz WS, and Hurlemann R (2013) Genetic variation in dopaminergic activity is associated with the risk for psychiatric side effects of levetiracetam. *Epilepsia* **54**:36–44.

Hessen E, Lossius MI, Reinvang I, and Gjerstad L (2007) Slight improvement in mood and irritability after antiepileptic drug withdrawal: a controlled study in patients on monotherapy. *Epilepsy Behav* **10**:449–455.

Heyman E, Lahat E, Levin N, Berkovitch M, and Gandelman-Marton R (2012) Preliminary efficacy and safety of lacosamide in children with refractory epilepsy. *Eur J Paediatr Neurol* **16**:15–19.

Hirai K, Kimiya S, Tabata K, Seki T, Jozaki K, and Kumagai N (2002) Selective mutism and obsessive compulsive disorders associated with zonisamide. *Seizure* **11**:468–470.

Hsu WWQ, Sing CW, He Y, Worsley AJ, Wong ICK, and Chan EW (2013) Systematic review and meta-analysis of the efficacy and safety of perampanel in the treatment of partial-onset epilepsy. *CNS Drugs* **27**:817–827.

Huber B, Bocchicchio M, Feuerbaum E, May T, Meinert T, Robertson E, Schorlemmer H, Wagner W, Wilking E, and Seidel M (2008) Efficacy and tolerability of pregabalin in patients with difficult-to-treat epilepsy and intellectual disability. *Epilepsy Behav* **13**:397–401.

Huber B, May T, and Seidel M (1998) Lamotrigine in multihandicapped therapy-resistant epileptic patients. *Clin Drug Investig* **16**:263–277.

Inoue Y, Ohtsuka Y, Oguni H, Tohyama J, Baba H, Fukushima K, Ohtani H, Takahashi Y, and Ikeda S (2009) Stiripentol open study in Japanese patients with Dravet syndrome. *Epilepsia* **50**:2362–2368.

Jan MM, and Shaabat AO (2000) Clobazam for the treatment of intractable childhood epilepsy. *Neurosci Riyadh Saudi Arab* **5**:159–161.

Juhl S, and Rubboli G (2014) Perampanel as add-on treatment in patients with drug-resistant focal epilepsy: the clinical experiences of the Danish Epilepsy Center (abstract p348). *Epilepsia* **55 (Suppl. 2)**:115.

Kalachnik JE, Hanzel TE, Sevenich R, and Harder SR (2002) Benzodiazepine behavioral side effects: review and implications for individuals with mental retardation. *Am J Ment Retard* **107**:376–410.

Kanemura H, Sano F, Ohyama T, Sugita K, and Aihara M (2014) Effect of levetiracetam on behavioral problems in pervasive developmental disorder children with epilepsy. *Eur J Paediatr Neurol* **18**:482–488.

Kang BS, Moon HJ, Kim Y-S, Lee S-T, Jung K-H, Chu K, and Lee SK (2013) The long-term efficacy and safety of levetiracetam in a tertiary epilepsy centre. *Epileptic Disord* **15**:302–310.

Kankirawatana P, Raksadawan N, and Balangkura K (2002) Vigabatrin therapy in infantile spasms. *J Med Assoc Thai* **85 Suppl 2**:S778–783.

Kato H, Fukatsu N, Noguchi T, Oshima T, Tadokoro Y, and Kanemoto K (2011) Lamotrigine improves aggression in patients with temporal lobe epilepsy. *Epilepsy Behav* **21**:173–176.

Kaufman KR, Kugler SL, and Sachdeo RC (2002) Tiagabine in the Management of Postencephalitic Epilepsy and Impulse Control Disorder. *Epilepsy Behav EB* **3**:190–194.

Kelly K, Stephen L, Parker P, and Brodie M (2014) Adjunctive perampanel in highly drug-resistant localization-related epilepsy - a prospective audit (abstract p119). *Epiliepsia* **55 (Suppl. 2)**:44.

Keogh S, Greenhill L, McCorry D, and Bagary M (2014) The efficacy and tolerability of perampanel (Fycompa) in everyday clinical practice using a retrospective multicenter audit (abstract p358). *Epilepsia* **55 (Suppl. 2)**:118.

Khurana DS, Riviello J, Helmers S, Holmes G, Anderson J, and Mikati MA (1996) Efficacy of gabapentin therapy in children with refractory partial seizures. *J Pediatr* **128**:829–833.

Kim JS, Kim H, Lim BC, Chae J-H, Choi J, Kim KJ, Hwang YS, and Hwang H (2014) Lacosamide as an adjunctive therapy in pediatric patients with refractory focal epilepsy. *Brain Dev* **36**:510–515.

Klehm J, Thome-Souza S, Sánchez Fernández I, Bergin AM, Bolton J, Harini C, Kadish NE, Libenson M, Peters J, Poduri A, Rotenberg A, Takeoka M, Bourgeois B, and Loddenkemper T (2014) Clobazam: effect on frequency of seizures and safety profile in different subgroups of children with epilepsy. *Pediatr Neurol* **51**:60–66.

Kottmel K, Guiblinger S, Pirker S, Freydl E, Langenberger E, Riederer F, Dedlacek C, and Baumgartner C (2014) First clinical experiences with perampanel in Vienna (abstract p573). *Epilepsia* **55 (Suppl. 2)**:186.

Krauss GL, Bar M, Biton V, Klapper JA, Rektor I, Vaiciene-Magistris N, Squillacote D, and Kumar D (2012) Tolerability and safety of perampanel: two randomized dose-escalation studies. *Acta Neurol Scand* **125**:8–15.

Krauss GL, Perucca E, Ben-Menachem E, Kwan P, Shih JJ, Clément J-F, Wang X, Bagul M, Gee M, Zhu J, and Squillacote D (2014) Long-term safety of perampanel and seizure outcomes in refractory partial-onset seizures and secondarily generalized seizures: results from phase III extension study 307. *Epilepsia* **55**:1058–1068.

Krauss GL, Serratosa JM, Villanueva V, Endziniene M, Hong Z, French J, Yang H, Squillacote D, Edwards HB, Zhu J, and Laurenza A (2012) Randomized phase III study 306: adjunctive perampanel for refractory partial-onset seizures. *Neurology* **78**:1408–1415.

Krishnamoorthy KS, Zalneraitis EL, Young RS, and Bernad PG (1983) Phenytoin-induced choreoathetosis in infancy: case reports and a review. *Pediatrics* **72**:831–834.

Kwan P, Trinka E, Van Paesschen W, Rektor I, Johnson ME, and Lu S (2014) Adjunctive brivaracetam for uncontrolled focal and generalized epilepsies: results of a phase III, double-blind, randomized, placebo-controlled, flexible-dose trial. *Epilepsia* **55**:38–46.

Labiner DM, Ettinger AB, Fakhoury TA, Chung SS, Shneker B, Tatum Iv WO, Mitchell Miller J, Vuong A, Hammer AE, and Messenheimer JA (2009) Effects of lamotrigine compared with levetiracetam on anger, hostility, and total mood in patients with partial epilepsy. *Epilepsia* **50**:434–442.

Lagae L, Buyse G, and Ceulemans B (2005) Clinical experience with levetiracetam in childhood epilepsy: an add-on and mono-therapy trial. *Seizure* **14**:66–71.

Lagae L, Velkey I, Bagul M, Yang H, Laurenza A, and Kumar D (2014) Impact of adjunctive perampanel on behaviour in adolescents with refractory partial-onset seizures (abstract p558). *Epilepsia* **55 (Suppl. 2)**:181.

Lander CM, Donnan GA, Bladin PF, and Vajda FJ (1979) Some aspects of the clinical use of clonazepam in refractory epilepsy. *Clin Exp Neurol* **16**:325–332.

Lee DO, Steingard RJ, Cesena M, Helmers SL, Riviello JJ, and Mikati MA (1996) Behavioral side effects of gabapentin in children. *Epilepsia* **37**:87–90.

Lee EH, Yum M-S, and Ko T-S (2013) Effectiveness and tolerability of rufinamide in children and young adults with Lennox-Gastaut syndrome: a single center study in Korea. *Clin Neurol Neurosurg* **115**:926–929.

Lee J-J, Song H-S, Hwang Y-H, Lee H-W, Suh C-K, and Park S-P (2011) Psychiatric symptoms and quality of life in patients with drug-refractory epilepsy receiving adjunctive levetiracetam therapy. *J Clin Neurol Seoul Korea* **7**:128–136.

Lee YJ, Kang H-C, Kim HD, and Lee JS (2010) Efficacy and safety of adjunctive levetiracetam therapy in pediatric intractable epilepsy. *Pediatr Neurol* **42**:86–92.

Levinson DF, and Devinsky O (1999) Psychiatric adverse events during vigabatrin therapy. *Neurology* **53**:1503–1511.

Levisohn PM, Mintz M, Hunter SJ, Yang H, Jones J, and on behalf of the N01103 Levetiracetam Study Group (2009) Neurocognitive effects of adjunctive levetiracetam in children with partial-onset seizures: A randomized, double-blind, placebo-controlled, noninferiority trial. *Epilepsia* **50**:2377–2389.

Livingston JH, Beaumont D, Arzimanoglou A, and Aicardi J (1989) Vigabatrin in the treatment of epilepsy in children. *Br J Clin Pharmacol* **27 Suppl 1**:109S–112S.

Loiseau P, Hardenberg JP, Pestre M, Guyot M, Schechter PJ, and Tell GP (1986) Double-blind, placebo-controlled study of vigabatrin (gamma-vinyl GABA) in drug-resistant epilepsy. *Epilepsia* **27**:115–120.

LoPresti A, Ettinger AB, Yang H, Williams B, Zhou S, Fain R, and Laurenza A (2014) Analysis of aggression in perampanel phase III epilepsy clinical trials (abstract 2.050). *Epilepsy Curr* **14 (S1)**:172–173.

Luna D, Dulac O, Pajot N, and Beaumont D (1989) Vigabatrin in the treatment of childhood epilepsies: a single-blind placebo-controlled study. *Epilepsia* **30**:430–437.

Lux AL, Edwards SW, Hancock E, Johnson AL, Kennedy CR, Newton RW, O'Callaghan FJK, Verity CM, and Osborne JP (2004) The United Kingdom Infantile Spasms Study comparing vigabatrin with prednisolone or tetracosactide at 14 days: a multicentre, randomised controlled trial. *Lancet* **364**:1773–1778.

Mbizvo GK, Dixon P, Hutton JL, and Marson AG (2014) The adverse effects profile of levetiracetam in epilepsy: a more detailed look. *Int J Neurosci* **124**:627–634.

McConnell H, Snyder PJ, Duffy JD, Weilburg J, Valeriano J, Brillman J, Cress K, and Cavalier J (1996) Neuropsychiatric side effects related to treatment with felbamate. *J Neuropsychiatry Clin Neurosci* **8**:341–346.

Mikkelsen B, Birket-Smith E, Bradt S, Holm P, BParm null, Lung M, Thorn I, Vestermark S, and Olsen PZ (1976) Clonazepam in the treatment of epilepsy. A controlled clinical trial in simple absences, bilateral massive epileptic myoclonus, and atonic seizures. *Arch Neurol* **33**:322–325.

Mula M, Agrawal N, Mustafa Z, Mohanalingham K, Cock HR, Lozsadi DA, and von Oertzen TJ (2015) Self-reported aggressiveness during treatment with levetiracetam correlates with depression. *Epilepsy Behav* **45**:64–67.

Mula M, and Trimble MR (2003) The importance of being seizure free: topiramate and psychopathology in epilepsy. *Epilepsy Behav EB* **4**:430–434.

Mula M, Trimble MR, Lhatoo SD, and Sander JWAS (2003) Topiramate and psychiatric adverse events in patients with epilepsy. *Epilepsia* **44**:659–663.

Mula M, Trimble MR, and Sander JW (2007) Are psychiatric adverse events of antiepileptic drugs a unique entity? A study on topiramate and levetiracetam. *Epilepsia* **48**:2322–2326.

Mula M, Trimble MR, and Sander JWAS (2004) Psychiatric adverse events in patients with epilepsy and learning disabilities taking levetiracetam. *Seizure* **13**:55–57.

Mula M, Trimble MR, Yuen A, Liu RSN, and Sander JW a. S (2003) Psychiatric adverse events during levetiracetam therapy. *Neurology* **61**:704–706.

Northam RS, Hernandez AW, Litzinger MJ, Minecan DN, Glauser TA, Mangat S, Zheng C, Souppart C, and Sturm Y (2005) Oxcarbazepine in infants and young children with partial seizures. *Pediatr Neurol* **33**:337–344.

Obeid M, and Pong AW (2010) Efficacy and tolerability of high oral doses of levetiracetam in children with epilepsy. *Epilepsy Res* **91**:101–105.

Opp J, Tuxhorn I, May T, Kluger G, Wiemer-Kruel A, Kurlemann G, Gross-Selbeck G, Rating D, Brandl U, Bettendorf U, Härtel C, and Korn-Merker E (2005) Levetiracetam in children with refractory epilepsy: a multicenter open label study in Germany. *Seizure* **14**:476–484.

Pal DK, Das T, Chaudhury G, Johnson AL, and Neville BG (1998) Randomised controlled trial to assess acceptability of phenobarbital for childhood epilepsy in rural India. *Lancet* **351**:19–23.

Paolicchi JM, Ross G, Lee D, Drummond R, and Isojarvi J (2015) Clobazam and Aggression-Related Adverse Events in Pediatric Patients With Lennox-Gastaut Syndrome. *Pediatr Neurol* **53**:338–342.

Parrett M, McLean B, Shankar R, and Cox D (2014) A service evaluation of perampanel in Cornwall, UK (abstract p334). *Epilepsia* **55 (Suppl. 2)**:110.

Peake D, Mordekar S, Gosalakkal J, Mukhtyar B, Buch S, Crane J, Wheway R, Rittey C, Donnelly J, Whitehouse WP, and Philip S (2007) Retention rate of levetiracetam in children with intractable epilepsy at 1 year. *Seizure* **16**:185–189.

Peltola J, Coetzee C, Jiménez F, Litovchenko T, Ramaratnam S, Zaslavaskiy L, Lu ZS, Sykes DM, and Levetiracetam XR N01235 Study Group (2009) Once-daily extended-release levetiracetam as adjunctive treatment of partial-onset seizures in patients with epilepsy: a double-blind, randomized, placebo-controlled trial. *Epilepsia* **50**:406–414.

Perry MS, and Benatar M (2007) Efficacy and tolerability of levetiracetam in children younger than 4 years: a retrospective review. *Epilepsia* **48**:1123–1127.

Philip S, Concannon B, Morris D, Seri S, and Agrawal S (2014) Efficacy and tolerability of perampanel in pharmacoresistant epilepsy in children and young people (abstract p126). *Epilepsia* **55 (Supp. 2)**:47.

Piña-Garza JE, Elterman RD, Ayala R, Corral M, Mikati MA, Piña-Garza MJ, Warnock CR, Conklin HS, and Messenheimer JA (2008) Long-term tolerability and efficacy of lamotrigine in infants 1 to 24 months old. *J Child Neurol* **23**:853–861.

Piña-Garza JE, Nordli DR, Rating D, Yang H, Schiemann-Delgado J, Duncan B, and Levetiracetam N01009 Study Group (2009) Adjunctive levetiracetam in infants and young children with refractory partial-onset seizures. *Epilepsia* **50**:1141–1149.

Piña-Garza JE, Schiemann-Delgado J, Yang H, Duncan B, Hadac J, and Hunter SJ (2010) Adjunctive levetiracetam in patients aged 1 month to <4 years with partial-onset seizures: subpopulation analysis of a prospective, open-label extension study of up to 48 weeks. *Clin Ther* **32**:1935–1950.

Pulliainen V, and Jokelainen M (1994) Effects of phenytoin and carbamazepine on cognitive functions in newly diagnosed epileptic patients. *Acta Neurol Scand* **89**:81–86.

Raucci U, Spalice A, Basile LA, Guardalà C, Nasta L, Terenzi S, and Iannetti P (1994) [New drugs in the treatment of childhood epilepsy: vigabatrin (study of 61 subjects)]. *Pediatr Medica E Chir Med Surg Pediatr* **16**:575–578.

Reith D, Burke C, Appleton DB, Wallace G, and Pelekanos J (2003) Tolerability of topiramate in children and adolescents. *J Paediatr Child Health* **39**:416–419.

Robinson MK, Richens A, and Oxley R (1990) Vigabatrin and behaviour disturbances. *Lancet* **336**:504.

Ronen GM, Richards JE, Cunningham C, Secord M, and Rosenbloom D (2000) Can sodium valproate improve learning in children with epileptiform bursts but without clinical seizures? *Dev Med Child Neurol* **42**:751–755.

Rosenfeld W, Conry J, Lagae L, Rozentals G, Yang H, Fain R, Williams B, Kumar D, Zhu J, and Laurenza A (2015) Efficacy and safety of perampanel in adolescent patients with drug-resistant partial seizures in three double-blind, placebo-controlled, phase III randomized clinical studies and a combined extension study. *Eur J Paediatr Neurol* **19**:435–445.

Ruuskanen I, Kilpeläinen HO, and Riekkinen PJ (1979) Side effects of sodium valproate during long-term treatment in epilepsy. *Acta Neurol Scand* **60**:125–128.

Ryvlin P, Werhahn KJ, Blaszczyk B, Johnson ME, and Lu S (2014) Adjunctive brivaracetam in adults with uncontrolled focal epilepsy: results from a double-blind, randomized, placebo-controlled trial. *Epilepsia* **55**:47–56.

Sackellares JC, Ramsay RE, Wilder BJ, Browne TR, and Shellenberger MK (2004) Randomized, controlled clinical trial of zonisamide as adjunctive treatment for refractory partial seizures. *Epilepsia* **45**:610–617.

Schiemann-Delgado J, Yang H, Loge C de la, Stalvey TJ, Jones J, Legoff D, and Mintz M (2012) A long-term open-label extension study assessing cognition and behavior, tolerability, safety, and efficacy of adjunctive levetiracetam in children aged 4 to 16 years with partial-onset seizures. *J Child Neurol* **27**:80–89.

Schmid G, and Huber B (2014) Limited benefit of perampanel in institutionalized cognitively impaired patients with highly therapy-resistant epilepsy (abstract p135). *Epilepsia* **55 (Suppl. 2)**:50.

Shehata GA, Bateh AEM, Hamed SA, Rageh TA, and Elsorogy YB (2009) Neuropsychological effects of antiepileptic drugs (carbamazepine versus valproate) in adult males with epilepsy. *Neuropsychiatr Dis Treat* **5**:527–533.

Sheth RD, Buckley D, Penney S, and Hobbs GR (1996) Vigabatrin in childhood epilepsy: comparable efficacy for generalized and partial seizures. *Clin Neuropharmacol* **19**:297–304.

Sheth RD, Goulden KJ, and Ronen GM (1994) Aggression in children treated with clobazam for epilepsy. *Clin Neuropharmacol* **17**:332–337.

Sheth RD, Ronen GM, Goulden KJ, Penney S, and Bodensteiner JB (1995) Clobazam for intractable pediatric epilepsy. *J Child Neurol* **10**:205–208.

Sieradzan K, and Hodgson H (2014) Efficacy and tolerability of perampanel in patients with refractory partial epilepsy in a tertiary epilepsy centre (abstract p137). *Epilepsia* **55 (Suppl. 2)**:50.

Specchio N, Balestri M, Striano P, Cilio MR, Nardello R, Patanè S, Margiotta ML, D’Orsi G, Striano S, Russo S, Specchio LM, Cusmai R, Fusco L, and Vigevano F (2010) Efficacy of levetiracetam in the treatment of drug-resistant Rett syndrome. *Epilepsy Res* **88**:112–117.

Steinhoff BJ, Ben-Menachem E, Ryvlin P, Shorvon S, Kramer L, Satlin A, Squillacote D, Yang H, Zhu J, and Laurenza A (2013) Efficacy and safety of adjunctive perampanel for the treatment of refractory partial seizures: a pooled analysis of three phase III studies. *Epilepsia* **54**:1481–1489.

Steinhoff BJ, Hamer H, Trinka E, Schulze-Bonhage A, Bien C, Mayer T, Baumgartner C, Lerche H, and Noachtar S (2014) A multicenter survey of clinical experiences with perampanel in real life in Germany and Austria. *Epilepsy Res* **108**:986–988.

Steinhoff BJ, Trinka E, Wieser H-G, and DACH-LEV Study Group (2005) Levetiracetam in patients with refractory epilepsy: results of the SKATE trial in Austria, Germany and Switzerland. *Seizure* **14**:490–496.

Stephen LJ, Kelly K, Parker P, and Brodie MJ (2011) Levetiracetam monotherapy--outcomes from an epilepsy clinic. *Seizure* **20**:554–557.

Stephen LJ, Sills GJ, and Brodie MJ (2000) Topiramate in refractory epilepsy: a prospective observational study. *Epilepsia* **41**:977–980.

Sveinbjornsdottir S, Sander JW, Patsalos PN, Upton D, Thompson PJ, and Duncan JS (1994) Neuropsychological effects of tiagabine, a potential new antiepileptic drug. *Seizure* **3**:29–35.

Tallian KB, Nahata MC, Lo W, and Tsao CY (1996) Gabapentin associated with aggressive behavior in pediatric patients with seizures. *Epilepsia* **37**:501–502.

Tartara A, Sartori I, Manni R, Galimberti CA, Di Fazio M, and Perucca E (1996) Efficacy and safety of topiramate in refractory epilepsy: a long-term prospective trial. *Ital J Neurol Sci* **17**:429–432.

Tay SK, Ong HT, and Low PS (2001) The use of vigabatrin in infantile spasms in Asian children. *Ann Acad Med Singapore* **30**:26–31.

Thomas L, Trimble M, Schmitz B, and Ring H (1996) Vigabatrin and behaviour disorders: a retrospective survey. *Epilepsy Res* **25**:21–27.

Valentin A, Moran N, Hadden R, Oakes A, Elwes R, Delamont R, Mullatti N, and Nashef L (2009) Pregabalin as adjunctive therapy for partial epilepsy: an audit study in 96 patients from the South East of England. *Seizure* **18**:450–452.

Van Paesschen W, Hirsch E, Johnson M, Falter U, and von Rosenstiel P (2013) Efficacy and tolerability of adjunctive brivaracetam in adults with uncontrolled partial-onset seizures: a phase IIb, randomized, controlled trial. *Epilepsia* **54**:89–97.

Vigevano F, and Cilio MR (1997) Vigabatrin versus ACTH as first-line treatment for infantile spasms: a randomized, prospective study. *Epilepsia* **38**:1270–1274.

Villanueva V, Garces M, and Lopez-Gonzalez FJ (2015) FYDATA study: retrospective analysis of perampanel in a real-life setting (abstract 1.332). *Epilepsy Curr* **15 (S1)**:156.

Voronkova KV, Pylaeva OA, and Petrukhin AS (2007) Efficacy of topiramate (Topamax) in epileptic patients of different ages. *Neurosci Behav Physiol* **37**:547–551.

Weber P, Dill P, and Datta AN (2012) Vigabatrin-induced forced normalization and psychosis--prolongated termination of behavioral symptoms but persistent antiepileptic effect after withdrawal. *Epilepsy Behav* **24**:138–140.

Weber S, and Beran RG (2004) A pilot study of compassionate use of Levetiracetam in patients with generalised epilepsy. *J Clin Neurosci* **11**:728–731.

Weintraub D, Buchsbaum R, Resor SR, and Hirsch LJ (2007) Psychiatric and behavioral side effects of the newer antiepileptic drugs in adults with epilepsy. *Epilepsy Behav* **10**:105–110.

Wheless JW, and Ng Y (2002) Levetiracetam in refractory pediatric epilepsy. *J Child Neurol* **17**:413–415.

White JR, Walczak TS, Leppik IE, Rarick J, Tran T, Beniak TE, Matchinsky DJ, and Gumnit RJ (2003) Discontinuation of levetiracetam because of behavioral side effects: a case-control study. *Neurology* **61**:1218–1221.

Wieshmann UC, and Baker GA (2013) Self-reported feelings of anger and aggression towards others in patients on levetiracetam: data from the UK antiepileptic drug register. *BMJ Open* **3**.

Wieshmann UC, Tan GM, and Baker G (2011) Self-reported symptoms in patients on antiepileptic drugs in monotherapy. *Acta Neurol Scand* **124**:355–358.

Willis J, Nelson A, Black FW, Borges A, An A, and Rice J (1997) Barbiturate anticonvulsants: a neuropsychological and quantitative electroencephalographic study. *J Child Neurol* **12**:169–171.

Wolf SM, Shinnar S, Kang H, Gil KB, and Moshé SL (1995) Gabapentin toxicity in children manifesting as behavioral changes. *Epilepsia* **36**:1203–1205.

Xiao Z, Li J-M, Wang X-F, Xiao F, Xi Z-Q, Lv Y, and Sun H-B (2009) Efficacy and safety of levetiracetam (3,000 mg/Day) as an adjunctive therapy in Chinese patients with refractory partial seizures. *Eur Neurol* **61**:233–239.

Yamamoto T, Pipo JR, Akaboshi S, and Narai S (2001) Forced normalization induced by ethosuximide therapy in a patient with intractable myoclonic epilepsy. *Brain Dev* **23**:62–64.

Zaccara G, Giovannelli F, Cincotta M, Verrotti A, and Grillo E (2013) The adverse event profile of perampanel: meta-analysis of randomized controlled trials. *Eur J Neurol* **20**:1204–1211.
